# Massive clonal expansion of polycytotoxic skin and blood CD8^+^ T cells in patients with toxic epidermal necrolysis patients

**DOI:** 10.1126/sciadv.abe0013

**Published:** 2021-03-19

**Authors:** Axel Patrice Villani, Aurore Rozieres, Benoît Bensaid, Klara Kristin Eriksson, Amandine Mosnier, Floriane Albert, Virginie Mutez, Océane Brassard, Tugba Baysal, Mathilde Tardieu, Omran Allatif, Floriane Fusil, Thibault Andrieu, Denis Jullien, Valérie Dubois, Catherine Giannoli, Henri Gruffat, Marc Pallardy, François-Loïc Cosset, Audrey Nosbaum, Osami Kanagawa, Janet L. Maryanski, Daniel Yerly, Jean-François Nicolas, Marc Vocanson

**Affiliations:** 1 Centre International de Recherche en Infectiologie (CIRI); INSERM, U1111; Université de Lyon 1; Ecole Normale Supérieure de Lyon; and CNRS, UMR 5308, Lyon, France.; 2 Drug Allergy Reference Center, Hospices Civils de Lyon, Hôpital Edouard Herriot, Service de Dermatologie, Lyon, France.; 3 Department of Rheumatology, Immunology and Allergology, Drug Allergy Research Laboratory, University Hospital of Bern, 3010 Bern, Switzerland.; 4 SFR Biosciences Gerland, US8, UMS3444, Lyon, France.; 5 Etablissement Français du Sang (EFS), Décines, France.; 6 INSERM, UMR 996, Châtenay-Mallabry, France.; 7 Département d’Allergologie et d’immunologie Clinique, Hôpital Lyon Sud, Pierre-Bénite, France.; 8 Unité de Thérapie Cellulaire et Génique (UTCG), Centre Hospitalier Universitaire de Nice, 06101 Nice, France.; 9 ADR-AC GmbH, Holligenstrasse 91, 3008 Bern, Switzerland.

## Abstract

Toxic epidermal necrolysis (TEN) is a life-threatening cutaneous adverse drug reaction. To better understand why skin symptoms are so severe, we conducted a prospective immunophenotyping study on skin and blood. Mass cytometry results confirmed that effector memory polycytotoxic CD8^+^ T cells (CTLs) are the main leucocytes in TEN blisters at the acute phase. Deep T cell receptor (TCR) repertoire sequencing identified massive expansion of unique CDR3 clonotypes in blister cells. The same clones were highly expanded in patient’s blood, and the degree of their expansion showed significant correlation with disease severity. By transducing α and β chains of the expanded clonotypes into a TCR-defective cell line, we confirmed that those cells were drug specific. Collectively, these results suggest that the relative clonal expansion and phenotype of skin-recruited CTLs condition the clinical presentation of cutaneous adverse drug reactions.

## Teaser

This study highlights the key role of polycytotoxic CD8^+^ T cells in the severity of toxic epidermal necrolysis.

## INTRODUCTION

Toxic epidermal necrolysis (TEN) is characterized as a rapidly progressing blistering eruption accompanied by an important mucosal involvement and skin detachment. Hence, TEN is associated with a high mortality rate of approximately 25 to 40% and nearly constant and invalidating sequelae, which are responsible for profound loss of quality of life in surviving patients (*1*–*3*).

The etiopathogenesis of TEN, similar to other cutaneous adverse drug reactions (cADRs), involves the activation of drug-specific T cells, which have been isolated and cloned from the blood and the skin lesions of patients with TEN (*4*–*7*). Similar to chemical haptens, the majorities of the drugs responsible for TEN are protein reactive and generate new drug-peptide epitopes that trigger a hypersensitivity/allergic reaction (*8*–*10*). Notably, recent works suggest that T cell stimulation could also be consecutive to a direct and noncovalent interaction of the drug with the T cell receptor (TCR) or the major histocompatibility complex (MHC)–binding groove (a process referred to as “p-i concept”) (*11*), as well as via the presentation of an altered repertoire of self-peptides (*12*, *13*).

The current paradigm for TEN onset states is that, once they have been primed in lymphoid organs, drug-specific cytotoxic CD8^+^ T cells (CTLs) are recruited at the dermo-epidermal junction where they kill keratinocytes presenting drug epitopes at their surface, through mechanisms involving perforin/granzyme B and MHC class I–restricted pathways (*6*, *10*). To explain the extensive blister formation and subsequent skin detachment, several investigators have reported that specific T cells produce massive amounts of soluble mediators such as granulysin (*14*), interferon-γ (IFN-γ), or tumor necrosis factor–α (TNF-α) that further amplify and extend keratinocyte cell death. IFN-γ and TNF-α promote Fas ligand (FasL) expression on keratinocytes, followed by cell-cell suicide (via Fas-FasL presentation), which may explain disseminated epidermal apoptosis in some patients (*15*). Alternatively, other studies have suggested that natural killer (NK) cells and inflammatory monocytes exert an additional contribution to epidermal necrolysis, notably via granulysin-dependent, TNF-like weak inducer of apoptosis (TWEAK/CD255)–dependent, TNF-related apoptosis inducing ligand (TRAIL/CD253)–dependent, or annexin A1–dependent mechanisms (*16*–*18*).

These immunological features are now well established, including the skin infiltration by CTLs (*19*). Yet, most of them have also been detected in patients suffering from less severe cADRs, such as maculopapular exanthema (MPE) (*20*, *21*). Patients with MPE harbor limited spots of epidermal apoptosis/necrolysis (*22*, *23*), but no blisters, and fast healing upon drug discontinuation. Hence, to date, it is still largely unknown why some patients, who sometimes take the same drugs (*24*, *25*), develop a severe and life-threatening disease (TEN) or a mild reaction (MPE). The fact that drug-specific CTLs are involved in diverse types of cADRs questions whether their number, their functions, or their activation parameters are specific to TEN disease. Moreover, the differential recruitment of unconventional cytotoxic leucocytes could also precipitate the severity of this disease.

To gain further insight on TEN pathogenesis, we conducted a comprehensive immunophenotyping study to characterize the immune cells infiltrating the skin or circulating in the blood of patients suffering from TEN or MPE at the time of disease diagnosis. Our results revealed a marked clonal expansion of polycytotoxic CD8^+^ T cells in the blood and skin of patients with TEN, which may affect final clinical severity.

## RESULTS

Skin and blood samples were collected from 18 TEN and 14 MPE hospitalized patients at the acute phase of their disease. Samples were recovered within 0 to 2 days after their hospital admission and diagnosis and within 0 to 5 days after the first symptoms (mainly fever and/or skin rash). Hence, most of the samples were collected before the peak of the disease, characterized for patients with TEN by the maximal percentage of skin detachment (Table 1 and table S1). Noteworthy, most of the patients displayed very diverse human leukocyte antigen (HLA) genotypes. A*02 and B*44 were the most represented loci (Table 1). A careful investigation of causative drug(s) associated to skin symptoms revealed a large variability in terms of drug nature or mode of action. The same molecule was reported as the culprit drug only for pairs of patients with TEN (allopurinol for patients TEN-1 and TEN-3, sulfamethoxazole/trimethoprim for TEN-2 and TEN-5, and ceftriaxone for TEN-10 and TEN-11; Table 1).

**Table 1:** Patient demographics, clinical features, and HLA genotype. ALDEN (algorithm for drug causality for epidermal necrolysis) was used to determine culprit drugs for patients with TEN. For patients with MPE, the main putative drugs are also indicated. Disease severity for patients with TEN was evaluated by the SCORTEN (score of TEN) at day 0 (arrival at hospital and diagnosis). The SCORTEN predicts the risk of death. The SCORTEN scale consists of seven independent factors for high mortality and varies from 0 or 1 (low mortality rate) to 5 or more (very high mortality rate). Disease severity was appreciated by calculating percentages of skin detachment (using E-Burn application). The peak of disease was appreciated as the date at which patients with TEN displayed maximal percentage of skin detachment. Disease severity for patients with MPE was estimated on the basis of the extent of skin rash and the presence of systemic and/or visceral symptoms. None of the patients with MPE exhibit symptoms suggestive of drug reaction and eosinophilia systemic symptoms/drug-induced hypersensitivity syndrome, and the Kardaun score was <3 for all the patients. M, male; F, female; ENT, ear nose throat; SLE, systemic lupus erythematosus; HIV^+^, HIV positive; HCV^+^, hepatitis C virus positive; na, not applicable; G-CSF, granulocyte colony-stimulating factor. *No culprit drug was identified for patient TEN-9, using ALDEN. The patient received ibuprofen, doxycycline, sulfamethoxazole/trimethoprim, tetracycline, isoniazid, and rifampicin in the days before TEN onset.

**Demographics**			**Clinical characteristics**									**HLA genotype**	
**Patient** **ID**	**Sex/** **age**	**Ethnicity**	**Underlying** **diseases**	**Comorbidities**	**Culprit drug**	**Drug** **exposure** **before** **onset** **(days)**	**Date and** **nature of** **first** **symptoms**	**SCORTEN** **(TEN)/** **severity** **(MPE)**	**% of skin** **detachment** **at day 0**	**% and date** **of maximal** **skin** **detachment**	**Treatment**	**Locus A**	**Locus B**
TEN-1	M/48	East Asian	Hyperuricemia	None	Allopurinol	8	Day 2/fever	3	2%	100% at day 2		A*02; A*33	B*38; B*58
TEN-2	M/39	European	Urine tract infection	None	Sulfamethoxazole/ trimethoprim	7	Day 2/fever	1	6%	20% at day 5	Systematic corticosteroid + G-CSF	A*30; A*30	B*13; B*18
TEN-3	F/40	European	Hyperuricemia	None	Allopurinol	15	Day 3/fever + skin rash	2	20%	80% at day 2		A*02; A*03	B*27; B*58
TEN-4	M/74	European	Melanoma	None	Vemurafenib	22	Day 4/skin rash	5	30%	100% at day 1	Maintenance of existing corticosteroid therapy + G-CSF	A*03; A*23	B*44; B*51
TEN-5	M/32	North African	Pneumocystis prophylaxis	HIV^+^	Sulfamethoxazole/ trimethoprim	15	Day 2/fever	3	10%	80% at day 2	Systematic corticosteroid + G-CSF	A*02; A*24	B*44; B*45
TEN-6	F/83	European	Urine tract infection	Cardiac insufficiency	Norfloxacin	8	Day 2/fever/ skin rash	3	20%	50% at day 5	G-CSF	A*03; A*-	B*18; B*73:01
TEN-7	M/50	European	Gastritis	Cirrhosis	Pantoprazole	10	Day 1/fever + skin rash	3	20%	100% at day 2	G-CSF	A*02; A*11	B*15; B*44
TEN-8	F/33	European	Bipolar disease	None	Lamotrigine	12	Day 3/fever + eye stinging	2	10%	40% at day 5		A*02; A*30	B*08; B*44
TEN-9	F/34	African American	Chronic pain	None	*	2	Day 2/fever	3	10%	50% at day 3	G-CSF	A*02; A*02	B*15; B*53
TEN-10	F/63	American	Severe angina	None	Ceftriaxone, ciprofloxacin	8	Day 4/skin rash	2	15%	30% at day 3		A*01:03; A*68	B*08; B*73:01
TEN-11	M/58	European	Infectious osteoarthritis	Diabetes, renal insufficiency	Ceftriaxone	15	Day 1/skin rash	4	10%	60% at day 2	G-CSF	A*02; A*29	B*44; B*45
TEN-12	F/27	European	Cirrhosis	Autoimmune hepatitis	Furosemide	21	Day 3/fever + skin rash	3	40%	40% at day 3	Maintenance of existing corticosteroid therapy + G-CSF	A*01; A*-	B*08; B*51
TEN-13	F/75	European	Postsurgery infection	Bladder adenocarcinoma	Cefixime	4	Day 1/fever + skin rash	4	30%	30% at day 2	G-CSF	A*02; A*-	B*44; B*57
TEN-14	H/41	European	Myeloma	None	Revlimid	15	Day 1 + fever + skin rash	2	5%	25% at day 3	Systematic corticosteroid	A*02; A*02	B*15; B*27
TEN-15	F/69	European	Lung infection	Ischemic stroke, SLE	Levofloxacin, metronidazole	5	Day 2 + fever + skin rash	3	10%	50% at day 3		A*03; A*30	B*18; B*40
TEN-16	F/69	European	Lung infection	None	Pristinamycain	1	Day 0/fever + skin rash	2	10%	38% at day 2		A*02; A*03	B*35; B*51
TEN-17	M/50	European	Infection	None	Azithromycin, paracetamol	5	Day 2/fever + skin rash	4	20%	80% at day 5	G-CSF	A*02; A*03	B*07; B*51
TEN-18	M/58	European	Liver cancer	HCV^+^	Sorafenib	10	Day 3/skin rash	5	5%	48% at day7	G-CSF	A*03; A*11	B*35; B*40
MPE-1	H/18	European	ENT infection	None	Amoxicillin	2	Day 1/skin rash	Mild	na	na	Topical corticosteroid	A*01; A*02	B*40; B*51
MPE-2	H/61	European	ENT infection	None	Amoxicillin	3	Day 1/skin rash	Mild	na	na	Topical corticosteroid	A*02; A*-	B*08; B*40
MPE-3	F/68	European	Breast infection	None	Vancomycin	28	Day 4/skin rash	Severe	na	na	Topical corticosteroid	A*24; A*25	B*15; B*18
MPE-4	F/78	European	Myeloma	None	Bortezomib	5	Day 4/skin rash	Severe	na	na	Topical corticosteroid	A*29; A*31	B*38; B*44
MPE-5	F/71	European	Cardiac insufficiency	None	Diltiazem	15	Day 3/skin rash	Severe	na	na	Topical corticosteroid	A*02; A*-	B*51; B*-
MPE-6	F/62	European	Infectious osteoarthritis	None	Vancomycin	2	Day 1/skin rash	Severe	na	na	Topical corticosteroid	A*01; A*02	B*40; B*57
MPE-7	H/61	North African	Pulmonary infection	None	Vancomycin	42	Day 4/skin rash	Mild	na	na	Topical corticosteroid	A*02; A*32	B*49; B*51
MPE-8	F/24	East Asian	Chronic pain	None	Ibuprofen	9	Day 3/skin rash	Severe	na	na	Topical corticosteroid	A*24; A*-	B*15; B*38
MPE-9	F/94	European	Urine tract infection	None	Clindamycin	3	Day 3/skin rash	Mild	na	na	Topical corticosteroid	A*23; A*31	B*39; B49
MPE-10	F/62	European	Graft-versus-host disease	Bone marrow transplant	Piperacillin/ tazobactam, contrast material	2	Day 1/skin rash	Mild	na	na	Topical corticosteroid	A*02; A*03	B*15:16; B*39
MPE-11	F/39	North African	Hypertension	SLE	Macrogol, urapidil, amlodipine	14	Day 3/skin rash	Moderate	na	na	Topical corticosteroid	A*32; A*34	B*39; B*44
MPE-12	F/62	European	Hypertension, gout	None	Allopurinol, fibrate	28	Day 2/skin rash	Mild	na	na	Topical corticosteroid	A*23; A*68	B*44; B*53
MPE-13	M/52	North African	Myeloma	None	Revlimid, bortezomid	15	Day 2/skin rash	Severe	na	na	Systemic corticosteroid	A*01; A*02	B*07; B*51
MPE-14	F/67	European	Dermatomyositis	None	Hydroxychloroquine	15	Day 4/skin rash	Mild	na	na	Topical corticosteroid	A*01; A*29	B*08; B*44

### Immunophenotype of leukocytes infiltrating the skin of patients with TEN

**Fig. 1:**
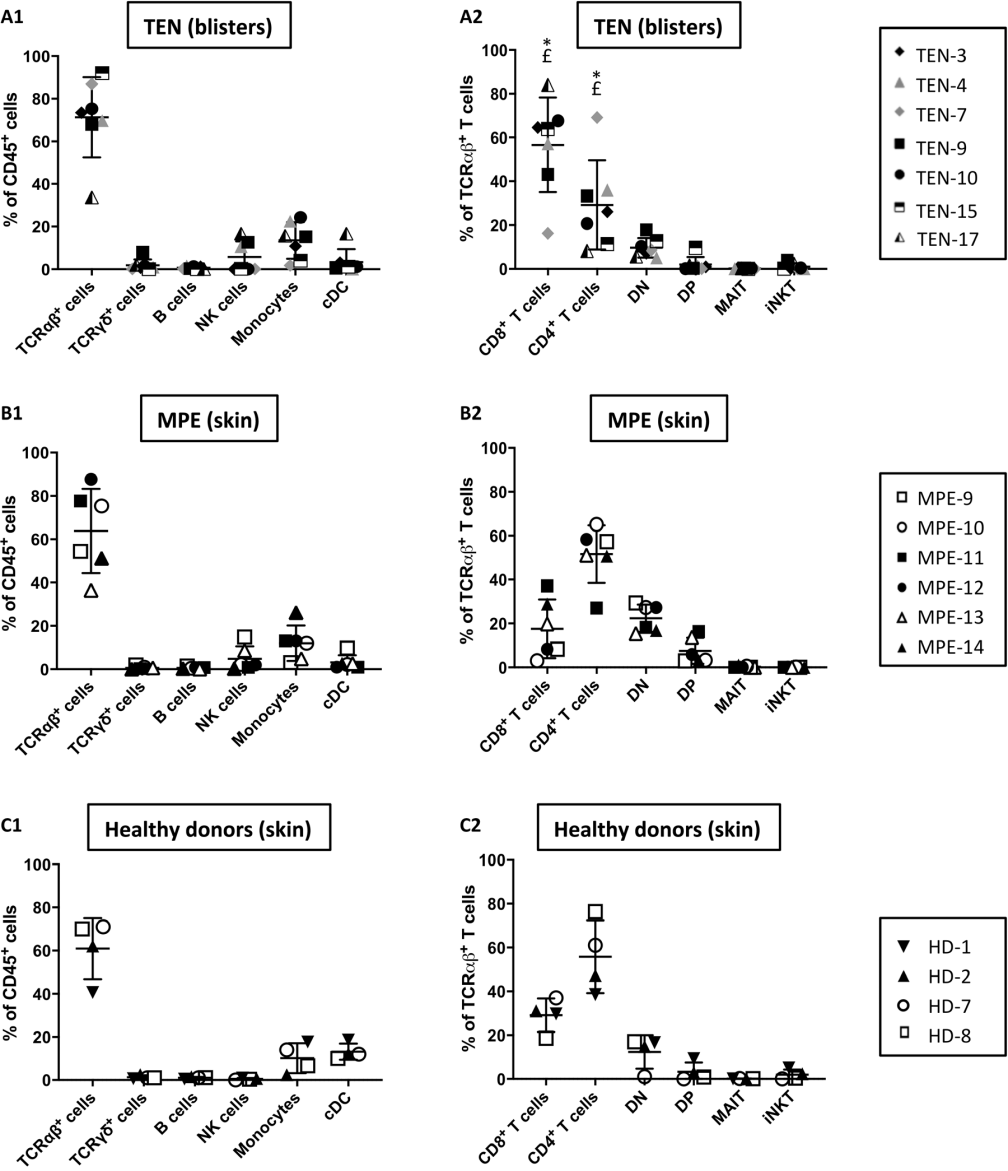
Immunophenotyping of leucocytes present in skin samples from TEN, MPE, or healthy donors. The leucocytes isolated from the blisters of seven patients with TEN (**A**) and the skin of six patients with MPE (**B**) and four healthy donors (**C**) were analyzed by mass cytometry. Scatterplots depict percentages of conventional TCRαβ^+^ lymphocytes, gamma delta T cells, B lymphocytes, NK cells, monocytes, or conventional dendritic cells (cDC) in CD45^+^ hematopoietic cells (**A1** to **C1**) and percentages of CD8^+^, CD4^+^, double-negative (DN), and double-positive (DP) T cell subsets, as well as iNKT and MAIT cells in gated TCRαβ^+^ population (**A2** to **C2**). Mean frequencies ± SD are also shown. Statistics compared frequencies of each subset in TEN versus MPE (*) or healthy (^£^) donor samples. *^,£^*P* < 0.05, Mann-Whitney test.

We first examined the immunophenotype of cells infiltrating the skin of patients with TEN by mass cytometry [cytometry by time of flight (CyTOF)] and subsequent computational data analysis. Blister cell samples obtained from seven patients with TEN were analyzed by CyTOF using a panel of 29 antibodies (table S2), enabling mapping of all major peripheral blood mononuclear cell (PBMC) subsets (lineage gating strategy is represented in fig. S1). We detected a large predominance of conventional T lymphocytes (TCRαβ^+^; mean ± SD = 71.3 ± 18.8%) among hematopoietic CD45^+^ cells, along with a minor infiltration of monocytes (CD14^+^ subset, 13.47 ± 8.6%); NK cells (TCRαβ^−^CD56^+^, 5.8 ± 7.2%); and very few gamma delta T (TCRγδ^+^, 1.9 ± 2.8%), B (CD19^+^, 0.6 ± 0.6%), or dendritic cells (CD11c^+^, 3.4 ± 5.9%) (Fig. 1A1). Conventional T lymphocytes were CD8^+^ (56.64 ± 21.6%), CD4^+^ (29.24 ± 20.4%), or double negative (9.6 ± 4.4%) T cells (Fig. 1A2), and rare double-positive (2.0 ± 3.4%), mucosal-associated invariant T (MAIT) (CD4^−^CD8β^−^TCRVα7.2^+^, 0.2 ± 0.1%) cells, or invariant NKT (iNKT; TCRαβ^int^TCRVα24^+^, 1.0 ± 1.5%) cells were recorded for all the patients (Fig. 1A2). When adjacent skin biopsies were collected instead of blister fluids, similar results were found, except for an increased representation of CD4^+^ versus CD8^+^ T cell fraction (fig. S2).

Similar to TEN, the inflamed skin of patients with MPE was infiltrated by conventional T lymphocytes (63.8 ± 19.5% of hematopoietic CD45^+^ cells) and, to a lesser extent, by CD14^+^ monocytes (12.3 ± 8.1%) and NK cells (4.8 ± 5.8%) (Fig. 1B1). In contrast to TEN, the CD4^+^ fraction (51.58 ± 13.2%) was greater than the CD8^+^ counterpart (17.6% ± 13.4) (Fig. 1B2). These frequencies were comparable to those found in the skin of healthy donors (Fig. 1C).

Last, we detected no major difference in the immunophenotype of cells circulating in the blood of patients with TEN, patients with MPE, and healthy donors, with CD8^+^ T cells representing approximately a quarter of total TCRαβ^+^ cells in all the tested samples (fig. S3). Collectively, these results thus confirm that the blistering and inflamed skin of patients with TEN is extensively infiltrated by CD8^+^ T cells (*14*, *26*, *20*). By contrast, no major skewing was recorded for unconventional lymphocytes, NK cells, or monocytes.

### Clustering of skin CD8^+^ T cells into seven phenotypic FlowSOM subsets

**Fig. 2:**
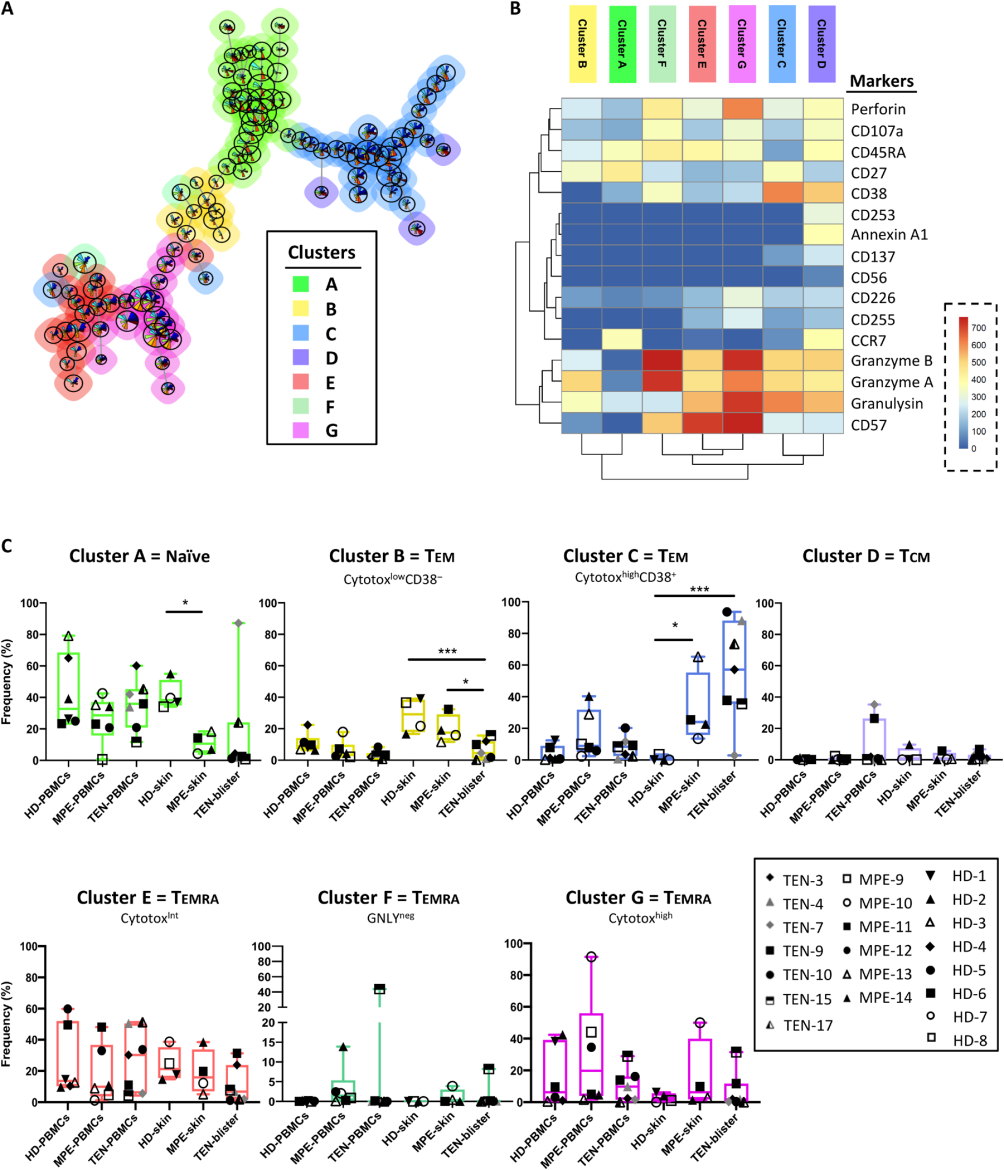
High-dimensional cell analysis of CD8^+^ T cells identifies TEN-enriched immunophenotypes. FlowSOM analysis with automatic consensus clustering was performed on concatenated CD8^+^ T cell data (300 cells per sample) from both lesion (blisters/skin) and PBMC samples from patients with TEN, patients with MPE, and healthy donors (HD). (**A**) Results were presented as minimal spanning tree (MST) of 100 nodes gathered in seven background colored clusters (A to G). Each node includes phenotypically similar cells, and the size of the node indicates the number of cell events. See MST magnification in fig. S4 to understand marker expression in respective SOM nodes. (**B**) Heatmap of the integrated MFI of 16 markers across the seven FlowSOM clusters identified in (A). The color in the heatmap represents the median of the arcsinh for each cluster (centroid) with 0 to 1 transformed marker expression. Clusters (columns) and markers (rows) were hierarchically metaclustered using Ward’s method to group subpopulations with similar phenotype. (**C**) Cluster frequencies were determined for each sample from each individual to understand tissue abundance. Statistics compared frequencies of each cluster in PBMC or skin samples versus the frequency of the respective cluster in healthy donor samples. *P < 0.05 and ***P < 0.01, Mann-Whitney test (two tailed). GNLY, granulysin.

As CD8^+^ T cell–mediated cytotoxicity is key in the initiation and formation of drug-induced lesions, we investigated in detail the molecular cytotoxic expression patterns of CD8^+^ T cells in TEN blisters. We performed high-dimensional profiling and investigated the (co)expression of several cell death–associated molecules [not only granulysin, granzyme B, granzyme A, and perforin but also TRAIL (CD253), TWEAK (CD255), annexin A1, and CD107a], as well as different activation markers (CD27, CD38, CD56, CD57, CD137, and CD226). Using concatenated CyTOF data from different samples (blisters, skin, and PBMCs from not only TEN and MPE but also healthy donors), we ran FlowSOM, a self-organizing map (SOM) clustering algorithm, to assess the heterogeneity of the CD8^+^ T cell population present in the different patients. FlowSOM first stratified the CD8^+^ T cell population into 100 nodes. Projected as minimal spanning tree in Fig. 2A and fig. S4, each SOM node groups cells with similar phenotypes, with the node size representing the number of cells within that node. Illustrations of minimal spanning tree obtained for each tissue sample are also shown in fig. S5. SOM nodes were next gathered in four main clusters, as automatically calculated using *K*-finder tree-level approach algorithm. Because *K*-finder approach did not capture the full diversity of the concatenated population, we decided to increase the FlowSOM clustering to seven distinct clusters (clusters A to G). To define the phenotype identity of each cluster, we generated a heatmap showing the integrated median fluorescence intensity (MFI) values of each marker in each FlowSOM cluster (Fig. 2B). Figure S6 shows FACS (fluorescence-activated cell sorting) analysis of the seven FlowSOM CD8^+^ T cell clusters.

Cluster A displayed a phenotypic identity coincident with naïve T cells (characterized by high levels of CD45RA, CCR7, or CD27 and by the lack of classical cytotoxic markers such as granulysin, granzyme B, granzyme A, or perforin), while clusters E, F, and G recapitulated the main features of T_EMRA_ (effector memory T cell reexpressing CD45RA) cells, i.e., high levels of CD45RA and CD57 and low levels of CCR7 and with granulysin, granzyme B, granzyme A, or perforin as the main variables between clusters (low, moderate, and high cytotoxicity, respectively, for clusters E, F, and G, but with no granulysin expression in cluster F) (Fig. 2B and fig. S6). Alternatively, clusters B and C both displayed a phenotype of effector memory lymphocytes (TEM; CCR7^−^ and CD45RA^−^), but conversely to the former, cluster C was characterized by a phenotype of activated cytotoxic cells, as illustrated by their high level of CD38, granzyme B, and granulysin expression (Fig. 2B and fig. S12). The cluster D subpopulation bore some of the hallmarks of central memory T cells (TCM; CCR7^+^ and CD45RA^−^), as well as an elevated expression of CD38, annexin A1, and CD253 markers (Fig. 2B and fig. S6).

### A polycytotoxic signature typifies lesional TEN CD8^+^ T cells

The in-depth FlowSOM analysis allowed a comparison of the frequency of the CD8^+^ T cell clusters in lesion (blisters and skin) and blood samples from patients with TEN, patients with MPE, and healthy donors (Fig. 2C). Most of the clusters were present in all patient samples, except for clusters D and F found only in two and three patients, respectively. A degree of interindividual variation was found for most clusters. Notably, the activated polycytotoxic effector memory subset (cluster C) was consistently elevated in TEN (mean, 55% of infiltrating CD8^+^ T cells) and, to a lesser extent, in MPE (mean, 30%) skin samples, relative to healthy donor (mean, 1%) samples (Fig. 2C). Unlike the other clusters, cluster C expressed high levels of the cell surface activation marker CD38. These results thus establish that the major subset of TEN blister CD8^+^ T cells displays a hallmark CD38^+^ polycytotoxic effector memory cell phenotype (cluster C).

### Restricted TCRVβ repertoire among TEN blister and blood CD8^+^ T cells

**Fig. 3:**
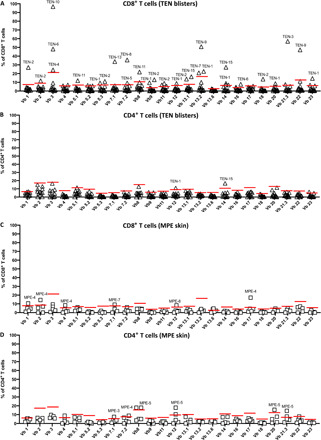
TCRVβ repertoire usage in T cell subsets isolated from the lesional skin of patients with TEN and MPE. The leucocytes isolated from the blisters of 13 individuals with TEN (**A** and **B**) and the lesional skin of 5 individuals with MPE (**C** and **D**) were analyzed by flow cytometry. Histograms depict percentages of the 24 TCRVβ chains in gated CD8^+^ (A and C) and CD4^+^ (B and D) T cell subsets, using the IOTest Beta Mark TCR Vβ Repertoire Kit (TCRVβ 1 to 4, 5.1, 5.2, 5.3, 7.1, 7.2, 8, 9, 11, 12, 13.1, 13.2, 13.6, 14, 16 to 18, 20, 21.3, and 22, 23). Each symbol (triangles for TEN and squares for MPE) represents a different individual. The red bar illustrates the threshold value from which TCRVβ chains were considered as highly expanded [using Tukey’s rule for the detection of outliers, i.e., Q3 + 1.5 × interquartile range (IQR)].

Parallel to these studies, we also addressed TCR usage of T cells present in TEN blisters. FACS analysis conducted on 24 of the most common Vbeta (Vβ) chains found a highly restricted TCRVβ repertoire usage in the 13 patients with TEN tested, with single Vβ expansions ranging from as much as 20 to 80% of total TCRVβ chains expression, when compared to healthy donors (Fig. 3 and fig. S7). This preferential usage, detectable at the CD3^+^ population level (fig. S8), concerned almost exclusively CD8^+^ (Fig. 3A) and rarely CD4^+^ T cells (Fig. 3B). It concerned quasi all the 24 Vβ chains (with the exception of Vβ4, Vβ5.2, Vβ13.6, and Vβ17, using antibody Vβ nomenclature). Vβ3 and Vβ13.2 were the most overrepresented Vβ chains, each found in 3 of 13 patients with TEN. Patients TEN-1 and TEN-2 showed overexpression of at least six TCRVβ chains, and TEN-9 exhibited two dominant Vβ13.2^+^ and Vβ22^+^ chains, each representing approximately 45% of total TCRVβ repertoire for this patient (Fig. 3A).

Although less marked than in TEN blisters, TCRVβ expansions were observed in CD8^+^ T cells (but not CD4^+^ T cells) from TEN PBMCs, with notable biases in patients TEN-3 to TEN-6, TEN-10, TEN-11, TEN-13, and TEN-15 (fig. S9, A and B). In contrast, a limited number of TCRVβ expansions were detected in CD8^+^ and CD4^+^ T cells isolated from MPE skin (Fig. 3, C and D) and PBMC samples (fig. S9, C and D), when compared to healthy donors (fig. S7).

### Massive oligoclonal expansion of the TCR complementarity-determining region 3 (CDR3, the antigen recognition domains) to evaluate sample clonality

As FACS cannot catch the full spectrum of the TCR repertoire, we next used high-throughput sequencing (HTS) of the TCR CDR3 regions (the antigen recognition domains) to evaluate sample clonality. HTS was performed on total blister, skin, and PBMC samples from patients with TEN and MPE.

**Fig. 4:**
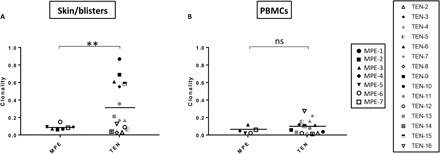
Increased clonality indices in TEN blister but not TEN PBMC samples. TCR repertoire diversity was evaluated by HTS on total blister and skin (**A**) and PBMC (**B**) samples from 15 individuals with TEN and 7 individuals with MPE. Scatterplots depict Shannon entropy–based clonality indices for total productive TCR rearrangements. Exact dates of sample collection are reported in table S1. Values approaching 1 indicate a highly clonal repertoire in which a small number of rearrangements comprise a large portion of all immune cells. Conversely, values approaching 0 indicate a polyclonal repertoire where all rearrangements are present at an identical frequency. **P < 0.01. Student’s *t* test (two tailed). ns, not significant.

Investigations of TCR repertoire diversity, measured using Shannon entropy–based clonality index metric, first revealed the presence of a highly clonal repertoire in the blisters of approximately half of patients with TEN (Fig. 4A and table S3A). By contrast, no difference was detected among PBMCs from patients with TEN and MPE (Fig. 4B and table S3B) compared to healthy donors [data not shown table S4; for healthy donor comparison, data were retrieved from Adaptive Biotechnologies project on normal human PBMCs at www.adaptivebiotech.com/products-services/immunoseq/immunoseq-analyzer and from (*27*), thus including data from 44 healthy donors].

**Fig. 5:**
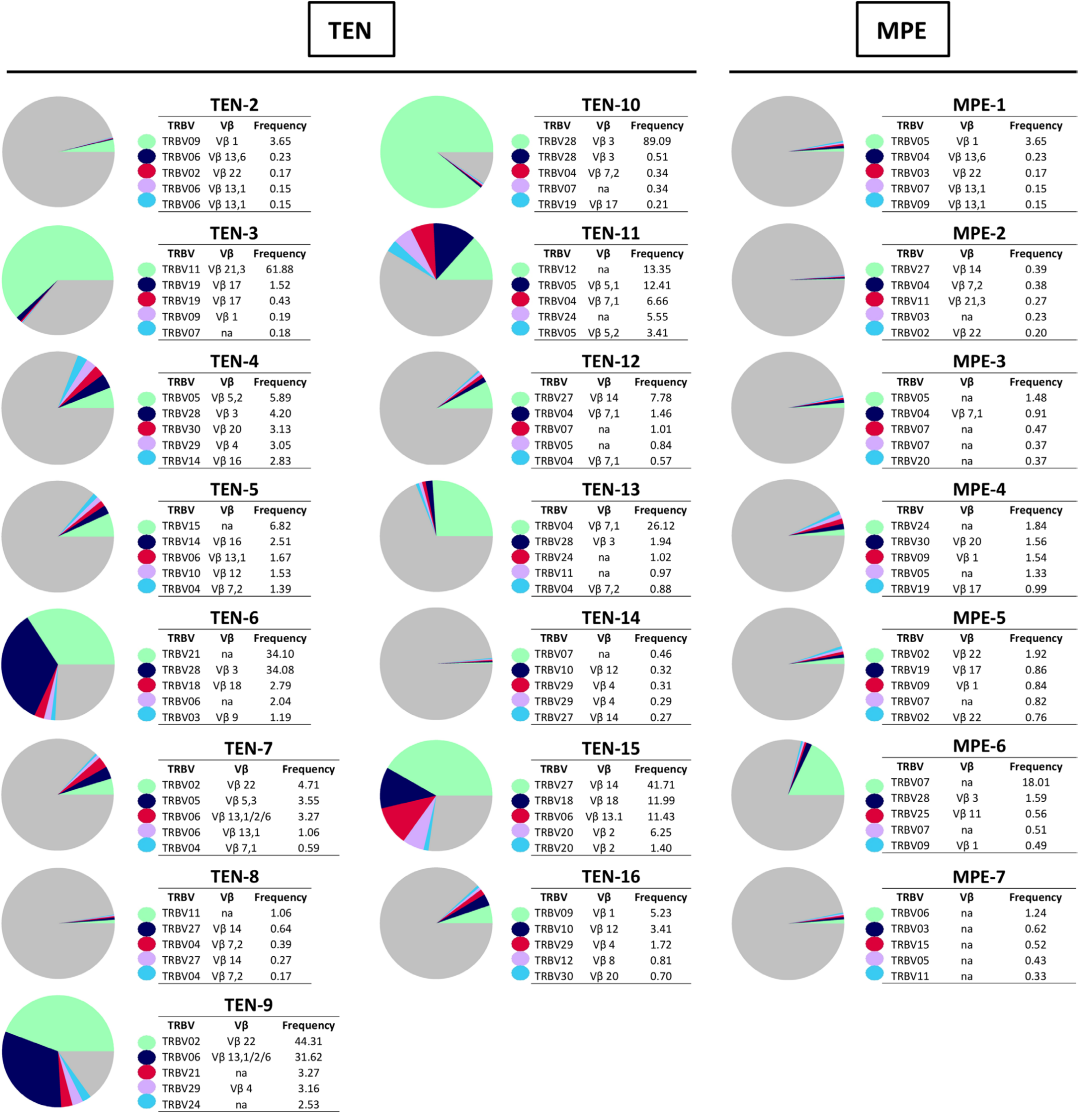
Frequency and TRBV usage of the highly expanded TCRβ clonotypes. The leucocytes isolated from the blisters of 15 individuals with TEN and the lesional skin of 7 individuals with MPE were evaluated using HTS of the TCR. Pie charts illustrate frequencies of the five most expanded TCRβ clonotypes (measured as percentage of unique CDR3 sequence among all productive rearrangements within a sample). Colors indicate the respective TRBV usage of each TCRβ clonotype. Gray indicates the remaining clonotypes found in the same sample. Cross-reference for the corresponding anti-Vβ monoclonal antibody (mAb) nomenclature is also provided.

In-depth analysis of the T-cell receptor beta variable region (TRBV) repertoire next confirmed the existence of preferential TCR biases in the blisters of 12 of 15 patients with TEN. These were the result of very limited numbers of CDR3 clonotype expansions, ranging from >10 to 90% of total TCR sequences for combined top five clones, except for TEN-2, TEN-8, and TEN-14 (Fig. 5 and tables S5). Notably, clone-tracking analyses revealed (i) that expanded clones expressed the same Vβ chain cells as those observed by FACS (table S5) and (ii) no sharing of identical TCR CDR3 nucleotide (fig. S10) or amino acid (fig. S11) sequences among the 15 patients with TEN. An interesting exception was noted for one clone from patients TEN-6 and TEN-10, which shared amino acid but not nucleotide sequence (figs. S10 and S11). As these patients were exposed to norfloxacin and ciprofloxacin quinolones, respectively, and both expressed HLA-B*73:03 (Table 1), potential epitope cross-reactivity could have led to the expansion of clones sharing identical TCRβ chains. Another interesting observation was noted for the two dominant clones from patient TEN-9 (Fig. 3). Parallel TRBV and TRAV (T-cell receptor alpha variable region) investigations performed on FACS-sorted CD8^+^TCRVβ13.2^+^ and CD8^+^TCRVβ22^+^ cells of this patient revealed that they represent, in fact, a unique T cell clone, which has rearranged two functional TCRβ genes (TRBV02-01*01 and TRBV06 sequences, respectively), as well as two functional TCRα genes (with the same TCRVA19-01*01 sequence, but distinct TRAJ segments, TRAJ30-01*01 and TRAJ29-01*01, respectively) (table S6).

Conversely to TEN, similar TRBV repertoire analysis revealed that clonal expansions were rare for patients with MPE and were usually lower than 5% (Fig. 5 and table S7). Clonotypes that were massively expanded in the TEN blisters were also found elevated in the blood of respective patients, at least for the top five clones (figs. S12 and S13 and tables S8 and S9). This result then indicates that the massive infiltration of unique clonotypes in TEN blisters was likely to be consecutive to a previous clonal expansion in the lymphoid organs. Only for patient TEN-15, and, to a lesser extent, for patients TEN-6 and TEN-11, were some of the highly expanded skin clones not represented in the blood (fig. S13).

### T cell repertoire diversity and clonal expansion of blister clones circulating in blood correlates with TEN severity

**Fig. 6:**
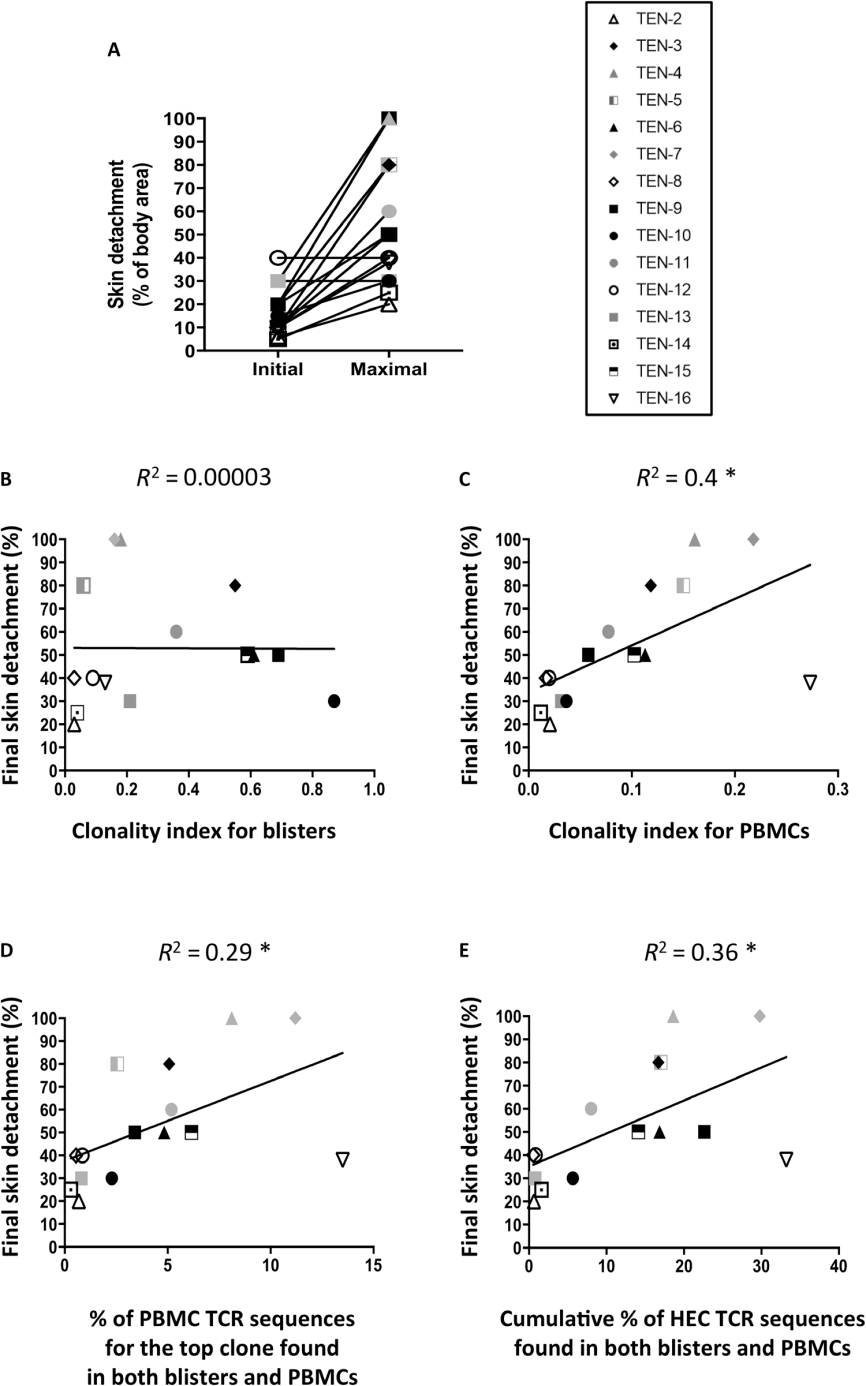
The percentage of maximal skin detachment in patients with TEN correlates with clonality indices and clonal expansion of skin clones in PBMCs. Quantification of skin detachment (expressed as percentage of total body area) in 15 patients with TEN was appreciated at their arrival at the hospital (initial) and at the peak of the skin reaction (maximal) (**A**). The latter was compared with the Shannon entropy–based clonality indices determined in blisters (**B**) and PBMCs (**C**). Correlations between clinical severity and the percentages of the top clone (**C**) or the cumulative percentages of the highly expanded clones (HEC) are also provided (**D**). Respective correlation factors were calculated using the Pearson correlation method. The coefficient of determination, *R*^2^, and statistical significance are indicated for each correlation. *P < 0.05, Student’s *t* test (two tailed).

TEN severity, assessed here as the percentage of final skin detachment, varied significantly after hospital admission (Fig. 6A) and was maximal between 1 and 7 days (mean ± SD = 3.2 ± 1.6 days; Table 1). We then investigated potential correlations between clonal expansions in the blisters or the blood of patients with TEN (measured at days 0 to 2 after hospital admission) and final skin detachment. While no association was detected with blister clonality indices (*R*^2^ = 0.00003, *P* is not significant; Fig. 6B), by contrast, we observed that patients with the highest PBMC clonality indices presented the highest percentage of final skin detachment (*R*^2^ = 0.4, *P* = 0.01; Fig. 6C). Besides, substantial correlations were also found between the percentage of top blister clones circulating in blood and the percentage of final skin detachment, as shown for the top one (*R*^2^ = 0.29, *P* = 0.04; Fig. 6D) and for the highly expanded clones, i.e., clones represented at a frequency >0.05% of TRBV repertoire in each patient (*R*^2^ = 0.36, *P* = 0.02; table S3 and Fig. 6E). Combined with the lack of major TCR CDR3 biases found in MPE samples (skin or blood) (Figs. 4 and 5), our results thus demonstrate that the massive expansion of unique clonotypes is a major feature of TEN pathology and that the level of expansion of those unique clonotypes among PBMCs at the acute phase is directly related to clinical severity.

### Vβ-expanded CD8^+^ T cells display the polycytotoxic phenotype overrepresented in TEN samples

**Fig. 7:**
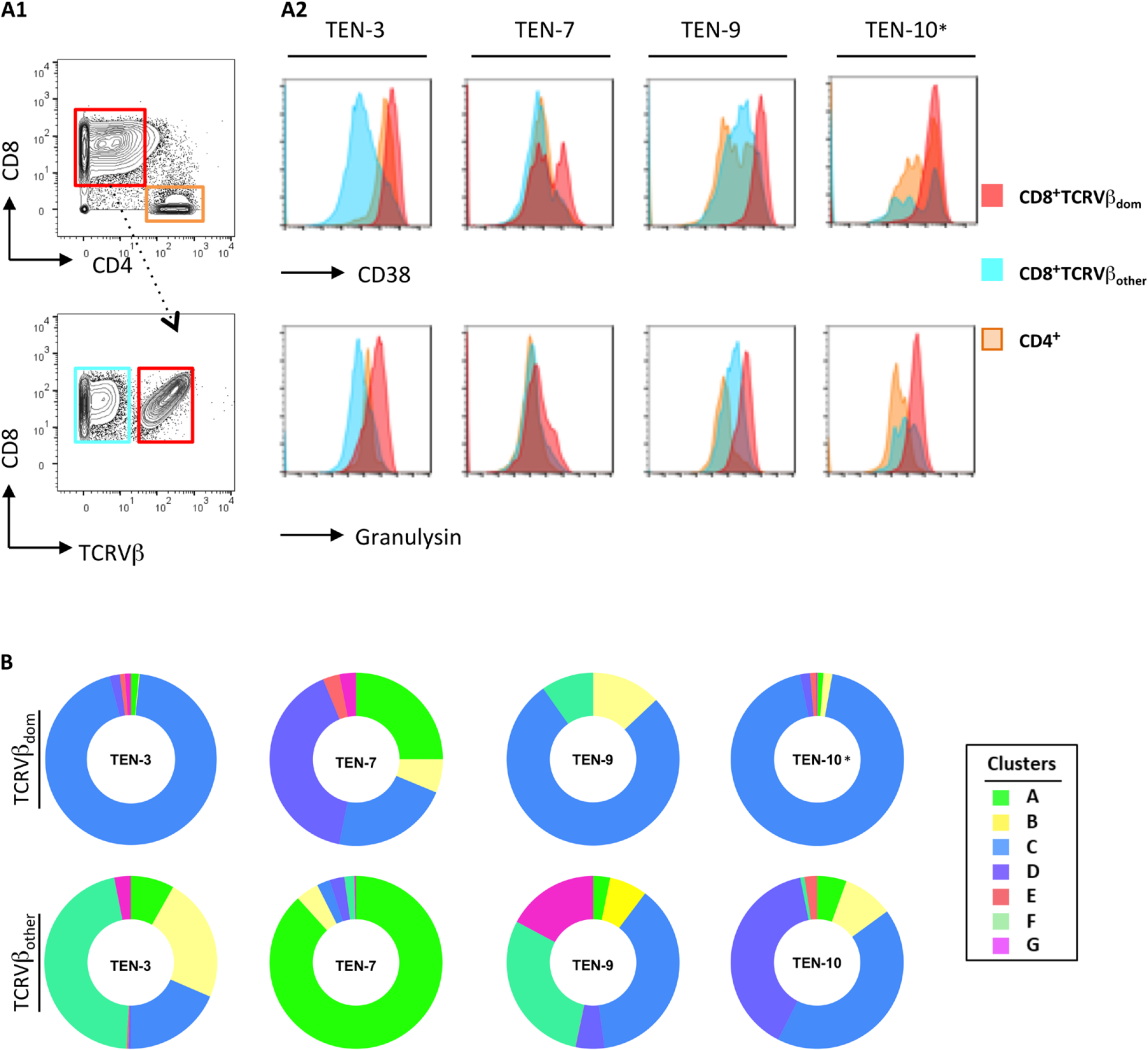
Immunophenotype of dominant TCRVβ^+^ cells. The dominant CD8^+^TCRVβ^+^ cell subset isolated from the blister fluids of four individuals with TEN (TEN-3, TEN-7, TEN-9, and TEN-10) was analyzed for the expression of CD38 and granulysin by mass cytometry (**A**). Pictures depict representative gating strategy to select the dominant CD8^+^TCRVβ^+^ cell subset [TCRVβ21.3^+^ for TEN-3, TCRVβ13.2^+^ for TEN-9 or TEN-7, and see * for TEN-10 (**A1**)] and histogram overlays of CD38 and granulysin expression, when compared with nondominant CD8^+^TCRVβ (others) or CD4^+^ T cell counterparts (**A2**). To characterize the phenotypic identity of respective subsets, CyTOF data were superimposed on concatenated CD8^+^ T cell clusters identified in Fig. 2. Donut representations depict the frequency of each cluster in dominant and nondominant CD8^+^TCRVβ^+^ cell subsets (**B**). *Notably, as no anti-Vβ3 mAb exists for CyTOF, dominant TCRVβ3^+^ cells in patient TEN-10 (which represent 90% of total CD8^+^ T cells in skin) were gated by negative selection. We gated cells negative for TCRVβ21.3^+^, TCRVβ13.2^+^, and TCRVβ7.2^+^ expression.

Thereafter, by taking advantage of mass cytometry, we were able to track back highly Vβ-expanded CD8^+^ T cells in the blisters and blood of patients with TEN to analyze their phenotype. We first demonstrated that CD8^+^ T cells expressing dominant Vβ chains (FACS analysis) displayed very high levels of granulysin and CD38 markers, when compared to their nondominant CD8^+^Vβ^+^ T cell counterparts (Fig. 7A). By superimposing dominant and nondominant TCRVβ^+^ markers on our concatenated CD8^+^ T cell clusters, we next demonstrated that dominant TCRVβ^+^ cells mainly expressed the cytotoxic cluster C phenotype (Fig. 7B). Conversely, the nondominant TCRVβ^+^ cells were detected in all the different clusters. This analysis confirms that major Vβ-expanded CD8^+^ T cells display the polycytotoxic phenotype that is overrepresented in TEN samples.

### Expanded clones in blisters and blood are drug specific

Ultimately, we sought to determine whether highly expanded and activated clones were drug specific. To this end, we FACS-sorted dominant CD8^+^TCRVβ^+^ T cells present in the blister fluids or the blood of four patients with TEN (TEN-3, TEN-7, TEN-10, and TEN-15) and sequenced their TRAV repertoire. For most dominant clones, a productive rearrangement (table S10) encoding a functional TCRα chain and a second nonproductive TCRα locus rearrangement (table S11) were identified. Then, the productive rearranged TCRα and TCRβ chains were transduced into Skw3 cells, a TCR-defective lymphoma line (table S12) (*28*). After verification of sustained and stable TCR expression (fig. S14), transduced Skw3 cells were stimulated with the culprit (or control) drug in presence of autologous Epstein-Barr virus (EBV)–transformed B cells generated from patient’s PBMCs. The following day, we measured CD69 expression at the surface of Skw3 cells as a marker for TCR stimulation.

Results showed a positive dose response for patient TEN-3 with oxypurinol (the metabolite of allopurinol, the culprit drug for TEN-3) but not with the parent drug or an irrelevant drug (sulfamethoxazole) (Table 2 and fig. S14). A positive response was also found for patient TEN-7 with the culprit pantoprazole (Table 2). In contrast, we failed to detect robust CD69^+^ expression in transductants generated from patients TEN-10 and TEN-15, stimulated respectively with ceftriaxone and ciprofloxacin or levofloxacin and metronidazole (Table 2).

**Table 2:** Drug-induced activation of TCRα Skw3 transductants. Skw3 cell lines engineered for the expression of TCRs bearing Vα and Vβ chains from top clones found in patients TEN-3, TEN-7, TEN-10, and TEN-15 were stimulated in vitro with EBV-transformed B cells in the presence of graded doses of different drugs or left unpulsed. This depicts the percentage of CD69 expression in CD3^+^ transductants measured by FACS after 24 hours of stimulation. Results from control transductants generated from abacavir-allergic (17D), allopurinol-allergic (AnWe A1), or sulfamethoxazole-allergic (UNO H13) donors (*7*, *52*, *54*) are also shown. Bold and underlined values indicate >2 or >1.5 CD69 expression fold increase versus unpulsed cultures. Transductant IDs are from table S12. Autologous EBV-transformed B cells were used for all the patients, except for patient TEN-7, for whom we did not have any autologous PBMCs available; hence, we performed the same analysis with heterologous PBMCs from different healthy donors. Heterologous EBV-transformed B cells were also used to stimulate control transductants.

			**% of CD69 expression in CD3^+^ transfectants**			
**Patient ID**	**Skw3 transfectant ID**	**Drug concentrations** **(μg/ml)**	**No drug**	**Concentration 1**	**Concentration 2**	**Concentration 3**
TEN-3	C1	Allopurinol (62.5/250)	2.3		2.2	3.05
		Oxypurinol (62.5/250)	2.3		**22.9**	**39.6**
		Sulfamethoxazole (100/200)	2.3		1.3	1.4
TEN-7	C2	Pantoprazole (10/50)	31.7		40.1	47.4
TEN-10	C3	Ceftriaxone (50/100/200)	12.2	11.9	12	13.8
		Ciprofloxacin (12.5/25/50)	10.9	10.1	11.8	10.6
TEN-15	C4	Levofloxacin (25/50/100)	6	5.6	5.4	4.4
		Metronidazole (25/50/100)	6.3	5.7	5.4	6
Control-1	17D	Abacavir (1/10/20)	1.4	**93.2**	**88.9**	**93.1**
		Pantoprazole (12.5/25/50)	1.4	1.8	1.8	1.7
Control-2	AnWe A1	Allopurinol (62.5/250)	4.3		**17.1**	**26.4**
		Oxypurinol (62.5/250)	4.3		5	5.75
Control-3	UNO H13	Ibuprofen (20/100/200)	5.2	4.6	**10.5**	**12.4**

## DISCUSSION

The main objective of our study was to gain further insights into TEN pathophysiology by tracking immune cells that are present in the skin and the blood of patients at disease onset. Our results confirm that CTLs are the main leukocyte subset found in TEN blisters, followed by a minor infiltration of CD14^+^ monocytes and NK cells, but we failed to repeatedly detect unconventional cytotoxic lymphocytes such as NK T, MAIT, or gamma delta T cells. Notably, deep sequencing of the TCR CDR3 repertoire revealed that there was a massive expansion of unique CD8^+^ T cell clones in patients with TEN (both in skin and blood), which express an effector memory phenotype and an elevated level of cytotoxic or inflammatory/activation markers such as granulysin, granzymes A and B, or CD38. By transducing α and β chains of the expanded clones into immortalized T cells, we demonstrate that some of these clones were drug specific. T cell repertoire diversity analysis revealed that clonal expansion of blister clones circulating in the blood of patients with TEN at the onset of the disease correlated with the final clinical severity, as defined by the maximal percentage of skin detachment.

The most marked observation of our study is certainly the demonstration that there is a marked expansion of unique polycytotoxic CD8^+^ T cell clones in patients with TEN, which largely outnumbers the frequency of clonotypes expanding in patients with less severe MPE. A few studies have already described oligoclonal expansion in TEN [or in the less severe Stevens-Johnson syndrome (SJS)]. These studies focused on in vitro T cell (re)activation experiments or used samples that were isolated from individuals with restricted HLA genotype, for instance, HLA-B*15.02 (*4*, *29*), and reactive to a limited number of compounds, mainly allopurinol and carbamazepine (*4*, *29*–*31*). They showed preferential usage of TRBV subtypes, clonal expansion of specific CDR3, and less TCR diversity in comparison to data obtained from healthy or drug-tolerant donors. Similarly, the infiltration of predominant T cell clones has already been reported in many benign inflammatory skin diseases such as psoriasis, atopic dermatitis, and contact dermatitis (and in MPE, as shown in our study; Fig. 5) (*32*, *33*). Here, novelty then resides in the demonstration that the strength of clonal expansions reached levels, in both blisters and blood, that have only been described in skin neoplasic disorders, such as cutaneous T cell lymphomas (*33*). In addition, the fact that our results can be generalized to patients expressing highly diverse HLA genotypes and reactive to very different drugs (Table 1) reinforces the idea that a massive clonal bias is a major immunological hallmark of TEN disease. Notably, as expected, we failed to detect any shared TCR sequences in our HLA diverse cohort, except for patients TEN-6 and TEN-10, exposed respectively to norfloxacin and ciprofloxacin quinolones and who both expressed HLA-B*73:03. Unfortunately, because of low sampling, it was not possible to compare TCR sequences from patients TEN-1 and TEN-3, harboring the HLA-B*58 risk allele and exposed to allopurinol.

It will be crucial to determine in the future the reasons for such clonal expansion in TEN disease compared to less severe MPE. (i) The massive production of inflammatory mediators noticed in the sera and the blister fluid of patients with TEN (*14*, *34*) certainly participates to enlarge the proliferation of drug-specific cells, but whether it is a consequence, a cause, or both remains to be clarified. (ii) T regulatory cells (T_regs_) are critical regulators of CTLs causing TEN in mouse models (*35*). In this context, the reported defective functions of TEN circulating T_regs_ and their decreased ability to infiltrate the skin (*36*, *37*) may explain the uncontrolled expansion and skin migration of drug-specific CTLs. Our data showed a differing CD4/CD8 ratio between TEN (ratio, 0.5) and MPE skin (ratio, 2) with MPE having a ratio similar to healthy skin (Fig. 1). This suggests that the skin CD4^+^ T_regs_/CD8^+^ CTL ratio may be a major parameter to control CTL activation in situ and, therefore, disease progression in TEN versus MPE. Future studies are needed to confirm the quantitative and qualitative defects of skin T_regs_ in TEN compared to MPE. (iii) Alternatively, it could be hypothesized that patients with TEN have a drug-specific preimmune repertoire that is prone to considerable enlargement. Several preclinical studies have shown that the breadth of immune response strongly depends on the number of specific T cell precursors (*38*), and a recent study from Pan *et al.* (*29*) showed an expansion of public TCRβ clonotypes in single HLA-restricted carbamazepine-allergic patients with SJS/TEN, questioning the possibility that patients with TEN with similar HLA and exposed to the same drug develop/amplify the same pathogenic T cell repertoire. (iv) Another assumption addresses heterologous immunity and a possible accumulation of pathogenic clones due to cross-reactivity with a reservoir of virus-specific memory T cells (*39*). (v) Last, it is still completely unknown whether drug accumulation [due to defective drug detoxification mechanisms (*40*)] predominates within TEN, fostering continuous T cell stimulation.

Another important point of the present study is the extended characterization of the expanded clonotypes, which mostly comprise CD8^+^ T cells endowed with a polycytotoxic phenotype. We observed that the dominant skin TCRVβ^+^ CTLs mainly expressed the cluster C phenotype, which was assigned to T_EM_ cells. As expected (*26*, *40*), this subset expressed high levels of granzyme A, granzyme B, and especially granulysin markers, and it was the only subset (with cluster D, poorly represented in skin samples) to express the CD38 protein, which is classically associated with T cell activation and/or diapedesis (*41*). By contrast, it lacked the expression of the senescence marker CD57 (classically assigned to T_EMRA_ subsets), indicating that the expanded CTL clones correspond to recently activated T cells.

By comparison, CD8^+^ T cells infiltrating the skin of MPE or healthy donors displayed a distinct functional phenotype, as shown both at the total population level (fig. S5) and after multidimensional analysis (Fig. 2). We notably detected less (MPE) or no (healthy donors) cluster C subset but more nonactivated T_EM_ cells (cluster B) and a T_EMRA_ subset (cluster E) endowed with moderate expression of cytotoxic markers (when compared to other T_EMRA_ subsets). It is probable that the main differences recorded between TEN and MPE (notably the differing CD4:CD8 ratio; Fig. 1) are due to the strong clonotype expansions and not the different type of tissues that we collected (TEN blisters versus MPE skin) because comparative analysis of adjacent blister skin in patients with TEN exhibited similar Vβ expansion and phenotype (figs. S15 to S17). It will be interesting to determine in future studies whether drug-specific skin MPE T cells are also found in cluster C, as for TEN (*20*, *26*). Besides, it will be important to uncover whether drug-specific T cells from patients with TEN have a unique ability to expand and/or to differentiate into potent killer cells when compared to MPE T cells. This challenging task might become feasible with T cell clones generated in vitro from precursors collected in patients with TEN and MPE allergic to the same molecules.

A major finding of our study is the antigenic specificity of the highly expanded clones found in patients with TEN. We were able to demonstrate that some of our engineered transductants (produced from TEN-3 and TEN-7) responded to their putative culprit drugs in vitro. Potential drug reactivity was also recorded with transductants, generated from the rearranged pairs of TCRβ and TCRα genes detected in the unusual dominant clone found in patient TEN-9, who was exposed to multiple drugs (Table 1). Nevertheless, as no clear culprit drug was identified for this patient, it was not possible to validate the relevance of our findings. In contrast, transductants generated from sequences identified in patients TEN-10 and TEN-15 failed to respond to the tested drugs (ceftriaxone, ciprofloxacin, levofloxacin, and metronidazole; Table 2). Various reasons might explain these TEN-10 and TEN-15 results. The simplest hypothesis is that we did not transfect the appropriate pathogenic TCR sequences. Alternatively, in keeping with the results obtained with TEN-3 transductants, which confirmed that T cells from allopurinol-allergic patients are reactive to its metabolite (oxypurinol) but not to the parent molecule (*4*), it is possible that our in vitro drug exposure conditions did not generate enough metabolites or drug-induced epitopes necessary to activate the transductants. Similarly, we cannot exclude that a specific mode of drug-epitope presentation, using peculiar nonconventional HLA presentation (*42*), or the involvement of an altered peptide repertoire (*12*, *13*) governs T cell expansion from patient TEN-10 or TEN-15.

The identification of early biomarkers, which predict final severity, is a highly desirable goal to improve clinical management of patients with TEN. Our data confirm and extend the recent study reported by Xiong *et al.* (*31*), which compared TCR repertoire diversity in patients suffering from SJS or TEN and showed that TCR repertoire metrics correlate with disease severity. So far, it is still debated whether SJS is an early stage of TEN (SJS is a bullous cADR characterized by <10% of skin detachment) or a different pathology, both at the etiological and mechanistic levels. Here, we enrolled patients with progressing but established TEN phenotype only, with 40 to 100% of skin detachment at the peak of disease, except for patient TEN-2 who displayed an SJS/TEN intermediate phenotype with 20% of skin detachment. Despite extensive clonal expansion in TEN blisters at disease onset, we failed to detect any correlation between blister TCR repertoire diversity (or the percentage of top skin clones, *R*^2^ = 0.00003, *P* = 0.9, and final skin severity; Fig. 6B). However, the same clones were also highly expanded in blood of patients with TEN, and the degree of their expansion in blood at the early phases of the disease showed significant correlation with the final disease severity (Fig. 6, C to E), thus expanding the findings reported by Xiong *et al.* (*31*). This suggests that the progression and severity of the disease is directly linked to the quantity of pathogenic clones that circulate in the blood and are able to be recruited in the epidermis a few hours/days after. Hence, to track clonal expansions, or TCR repertoire diversity, at disease onset could prove to be of paramount value for clinicians who want to anticipate the evolution of TEN and develop adequate care measures. However, because of the low number of patients (*n* = 15) tested in our TCR repertoire study, it will be crucial to validate our results with an extended cohort. Besides, it will be important to understand why there is no correlation with TCR repertoire metrics in the skin. The fact that we conducted this study on a prospective cohort with diverse HLA, reactive to different drugs of different half-lives and different pharmacological properties, suffering from different degrees of liver/kidney dysfunction, transferred for intensive care at different intervals after symptom onset, and withdrawn with culprit drug at different times and treated with different molecules (Table 1) certainly explains the discrepancies between the extent of final skin detachment and clonal expansion in the blisters at the beginning of the disease. It will be therefore crucial to conduct future studies on a more controlled cohort to decipher the reasons for the strong blood but not skin correlation.

In conclusion, our results demonstrate that the quantity and quality of skin-recruited CTLs condition the clinical presentation of cADRs. They open major opportunities for the development of new prognostic markers in TEN.

## MATERIALS AND METHODS

### Study design

Patients were prospectively recruited by the drug allergy reference center at the Hospices Civils de Lyon (France) between 2014 and 2018. TEN or MPE diagnoses were based on the definition published by the RegiSCAR study group (*43*, *44*). Only patients with a probable or a definite diagnosis of TEN or MPE were enrolled in this study. Culprit drugs in patients with TEN were determined according to the algorithm for drug causality for epidermal necrolysis (ALDEN) (*45*). For patients with MPE, the main putative drug was also determined. We collected demographic and clinical information, including sex and age, as well as underlying diseases (i.e., the disease that the culprit drug was prescribed for), comorbidities, duration of drug exposure before TEN/MPE onset, and HLA-A/B genotyping results. HLA-A/B genotypes were determined by reverse polymerase chain reaction sequence-specific oligonucleotide hybridization (LABType SSO, One Lambda, Eurobio-Ingen, Chilly-Mazarin, France). Additional information were also collected for patients with TEN: SCORTEN (score of TEN) at diagnosis, which aims to predict the severity of the disease (*46*) and percentage of skin detachment assessed by the E-Burn smartphone application (Android Play Store, Saint Joseph Saint Luc Hospital, Lyon, France). The latter was determined when the patient was first diagnosed with TEN (“initial”) and when maximum involvement was observed (“final”). We enrolled 20 healthy donors as controls.

A local ethical committee approved the study, and written informed consent was obtained from each participant. Given the observational nature of the translational study, there was no randomization or formal blinding process for the investigators.

### Sample collection and processing

#### Skin samples

Skin samples for TEN consisted of blister fluids and, for three patients, blister fluids and skin biopsies. Supernatant was collected, and cells were repeatedly washed in complete RPMI before subsequent processing. In cases of MPE and patients TEN-15, TEN-17, and TEN-18, 6-mm^2^ biopsies were performed directly into lesional erythematous skin. Abdominal skin leftovers, from healthy donors undergoing elective plastic surgery, were used as control biopsies. Skin cells were extracted by mechanical dissociation and enzymatic digestion [2 hours at 37°C in RPMI supplemented with collagenase type 1 (1.25 U/ml; Sigma-Aldrich, Saint Quentin Fallavier, France), deoxyribonuclease (4 kU/ml; Sigma-Aldrich), and Hepes buffer (5%)], before being passed through a 100-μm cell strainer (Thermo Fisher Scientific, Courtaboeuf, France) to obtain single-cell suspensions. Cell viability was determined by trypan blue exclusion.

#### Blood samples

PBMCs from healthy donors and patients were isolated from whole-blood samples (in lithium-heparin–coated tubes) using Ficoll Histopaque (Ficoll-Paque PLUS, Thermo Fisher Scientific) density gradient centrifugation, and cell viability was assessed as described above. Details about sampling days for each patient and corresponding investigations are listed in table S1. Depending on experiments, samples were either frozen in liquid nitrogen according to standard procedures or immediately stained for immunophenotyping analysis.

### Flow cytometry analysis

Flow cytometry was carried out using fluorescently labeled monoclonal antibodies (mAbs), recognizing human CD3 (7D6; Thermo Fisher Scientific), CD4 (VIT4; Miltenyi Biotec, Bergisch Gladbach, Germany), and CD8 (SK1; BioLegend, San Diego, CA, USA) proteins. Vβ chain repertoire expression was assessed using a kit of 24 TCRVβ mAbs (IOTest Beta Mark, Beckman Coulter, Roissy, France; which includes approximately 70% of the expressed human TCRVβ domains: TCRVβ 1, 2, 3, 4, 5.1, 5.2, 5.3, 7.1, 7.2, 8, 9, 11, 12, 13.1, 13.2, 13.6, 14, 16, 17, 18, 20, 21.3, 22, and 23), and viability discrimination was performed by incubating cells with live/dead eFluor 506 (eBioscience, San Diego, CA, USA).

Cells were analyzed on a LSRFortessa flow cytometer (BD Biosciences, Franklin Lakes, NJ, USA), and data were analyzed using FlowJo software (version 10; FlowJo, Ashland, OR, USA). For TCR sequencing experiments, some dominant CD8^+^ TCRVβ^+^ cells were sorted on a FACSAria IIu device (BD Biosciences).

### Mass cytometry analysis

Mass cytometry antibodies were obtained as preconjugated metal-tagged antibodies from Fluidigm (South San Francisco, CA, USA) or generated in-house by conjugating unlabeled purified antibodies (from Miltenyi or Beckman Coulter) to isotope-loaded polymers using Maxpar X8 Multimetal Labeling Kit (Fluidigm). After titration on NanoDrop ND 1000 (Thermo Fisher Scientific), antibodies were diluted in antibody stabilization buffer (CANDOR Biosciences, Wangen im Allgäu, Germany) with 0.5% sodium azide (Sigma-Aldrich). A detailed list of the antibodies used in this study is provided in the Supplementary Materials (table S2). Cell identification was performed using an iridium intercalator (Fluidigm), and viability discrimination was assessed by staining cells with Cisplatin (194Pt, Fluidigm). In some experiments, cells were fixed and permeabilized using Cytofix/Cytoperm solution (Cytofix/Cytoperm, BD Biosciences, Le Pont-de-Claix, France) and then stained with human anti-granulysin, anti–granzyme A, anti–granzyme B, and anti-perforin mAbs.

Before acquisition on Helios mass cytometer (Fluidigm), cells were resuspended in half-diluted Four Element Calibration Beads (Fluidigm), and the dataset was normalized with CyTOF software using Finck algorithm (*47*). Flow Cytometry Standard 3.0 files were imported into FlowJo software version 10, and analyses included standard gating to remove beads, aggregates, or dead cells and further identify main leukocyte subsets (fig. S1).

### High-dimensional mass cytometry data analysis

An inverse hyperbolic sine transformation was applied to analyze TCRαβ^+^ CD8^+^ T cells [*n* = 300 per sample; all CyTOF samples were used (table S1), except skin samples from MPE-9 and MPE-12, which were excluded from the analysis because of very low CD8^+^ T cell number, and TEN-18 samples because of technical problem]. Data were next clustered using FlowSOM algorithm (with FlowSOM R plugin downloaded for FlowJo version 10) (*48*). A SOM was first trained to gather all cells into 100 distinct nodes based on their similarities in high-dimensional space (i.e., considering the relative MFI of 16 markers simultaneously: CCR7, CD45RA, CD27, CD38, CD56, CD57, CD107a, CD137, CD226, CD253, CD255, granzyme A, granzyme B, granulysin, perforin, and annexin A1 and excluding cell lineage (CD45, CD14, CD19, TCRαβ, TCRγδ, CD8α, CD8β, CD4, CD38, CD56, NKP46, CD11b, CD11c, TCRVα14-Jα18, and TCRVα7.2). SOM nodes were subsequently grouped in different clusters (each representing different CD8^+^ T cell subsets) using *K*-parameter and/or *K*-Finder R package (https://arxiv.org/pdf/1811.07356.pdf; based on the Tracy-Widom algorithm to approximate “*K*” in sparse data matrices, *K* being the number of relevant clusters in a population) (*49*). FlowSOM clusters were visualized as integrated (i.e., including all samples) or disease phenotype minimal spanning trees, and heatmaps showing the integrated or individual MFI of each marker per cluster were generated with FlowJo or Excel. Additional hierarchical metaclusterings were performed, using the gplots R package based on the Euclidean distance and Ward linkage (*50*), to determine the immunophenotype or the frequency of each cluster per samples.

### DNA isolation and HTS of TCRα/β CD3R regions

DNA was isolated from frozen total blister, skin, and PBMC samples using the QIAamp DNA Micro Kit (QIAGEN, Courtaboeuf, France), according to the manufacturer’s instructions. Then, TCRβ and TCRα CDR3 regions were amplified and sequenced using immunoSEQ assay (Adaptive Biotechnologies, Seattle, WA, USA). Briefly, bias-controlled V and J gene primers were used to amplify rearranged V(D)J segments spanning each unique CDR3β/α, and amplicons were next sequenced (at approximately 20× coverage) using the Illumina HiSeq platform. The assay was performed at survey level (detection sensitivity, 1 cell in 40,000). After correcting sequencing errors via a clustering algorithm, TCRβ/α V, D, and J genes were annotated according to the ImMunoGeneTics information system (IMGT, www.imgt.org).

Sequencing data were analyzed according to the immunoSEQ Analyzer version 3.0 (www.immunoseq.com). Diverse TCR repertoire metrics were explored: frequency and overlap of highly expanded clones; respective nucleotide or amino acid CDR3 sequences; usage and pairing of TCRB/AV, TCRBD, and TCRB/AJ families; or diversity of the TCR repertoire (clonality index based on Shannon’s entropy).

### Transduction of the Vα and Vβ chains of the TCR into Skw3 cell lines

Skw3 cell lines (Leibniz Institute DSMZ, Brunswick, Germany) (*51*) were transduced as described previously (*52*). Briefly, rearranged human variable TCRα and TCRβ genes identified by TCR sequencing were synthesized by custom gene synthesis (Gene Universal, Newark, DE, USA) and cloned into retroviral pMSCV vector (Takara Bio USA Inc., Mountain View, CA, USA) containing puromycin and neomycin resistance genes, respectively. The resulting retroviruses were used to transduce the TCR-defective Skw3 cell line, which also expresses the human CD8 co-receptor. The TCR-transduced cells outgrowing in selective medium were picked, and the expression of the correct TCRα and TCRβ was further assessed by flow cytometry, using a FACSCanto I device (BD Biosciences, San Jose, CA, USA). The transduced cells with stable TCR expression were selected for assessment of reactivity and specificity, which was measured by TCR-induced CD69 expression.

### TCR-transductant stimulation assay

Skw3 cell lines expressing the cognate TCRα and TCRβ chains were cocultured with autologous EBV-transformed B-lymphoblastoid cell lines (*53*) at 1:2 ratio at 37°C. Tested drugs were added to the cocultures with the indicated concentrations. After 24 hours, cells were stained with anti-human CD3 (BioLegend) and anti-human CD69 (BioLegend) and analyzed by flow cytometry. Levels of CD69 expression was monitored in 10,000 CD3^+^ events. Experiments were repeated at least two times.

### Statistical analysis

*P* values were calculated with two-tailed independent Student’s *t* tests or one-way analysis of variance (ANOVA) using GraphPad Prism software (version 8; San Diego, CA, USA). *P* values <0.05 were considered significant.

The Tukey’s rule for the detection of outliers [75th percentile (Q3) + 1.5 × interquartile range (IQR)] was used to identify highly expanded TCRVβ chains. Notably, all TEN, MPE, and healthy donor data for each Vβ chain were compiled to calculate percentiles and IQR.

## Supplementary Material

http://advances.sciencemag.org/cgi/content/full/7/12/eabe0013/DC1

Adobe PDF - abe0013_SM.pdf

Masive clonal expansion of polycytotoxic skin and blood CD8+ T cells in patients with toxic epidermal necrolysis

## SUPPLEMENTARY MATERIALS

Supplementary material for this article is available at http://advances.sciencemag.org/cgi/content/full/7/12/eabe0013/DC1

## Appendix

View/request a protocol for this paper from *Bio-protocol*.
