# Supplementary material for: Massive clonal expansion of polycytotoxic skin and blood CD8+ T cells in patients with toxic epidermal necrolysis patients
Source: Sci Adv. 2021 Mar 19;7(12):eabe0013. doi: 10.1126/sciadv.abe0013 (PMC7978430; doi:10.1126/sciadv.abe0013)
Supplement: http://advances.sciencemag.org/cgi/content/full/7/12/eabe0013/DC1 [file supp_7_12_eabe0013__7.12.eabe0013.DC1.html]

Science Advances | Science AdvancesAAASSearchScience AdvancesMenu

## Supplementary Materials

# Massive clonal expansion of polycytotoxic skin and blood CD8+ T cells in patients with toxic epidermal necrolysis

Axel Patrice Villani, Aurore Rozieres, Benoît Bensaid, Klara Kristin Eriksson, Amandine Mosnier, Floriane Albert, Virginie Mutez, Océane Brassard, Tugba Baysal, Mathilde Tardieu, Omran Allatif, Floriane Fusil, Thibault Andrieu, Denis Jullien, Valérie Dubois, Catherine Giannoli, Henri Gruffat, Marc Pallardy, François-Loïc Cosset, Audrey Nosbaum, Osami Kanagawa, Janet L. Maryanski, Daniel Yerly, Jean-François Nicolas, Marc Vocanson

Download Supplement

**This PDF file includes:**

- Tables S1 to S12
- Figs. S1 to S17

**Files in this Data Supplement:**

- Adobe PDF - abe0013\_SM.pdf
