# Supplementary material for: Massive clonal expansion of polycytotoxic skin and blood CD8+ T cells in patients with toxic epidermal necrolysis patients
Source: Sci Adv. 2021 Mar 19;7(12):eabe0013. doi: 10.1126/sciadv.abe0013 (PMC7978430; doi:10.1126/sciadv.abe0013)
Supplement: Adobe PDF - abe0013_SM.pdf [file abe0013_SM.pdf]

Science Advances
AAAS

advances.sciencemag.org/cgi/content/full/7/12/eabe0013/DC1

Supplementary Materials for

Massive clonal expansion of polycytotoxic skin and blood CD8$^+$ T cells in patients with toxic epidermal necrolysis patients

Axel Patrice Villani, Aurore Rozieres, Benoît Bensaid, Klara Kristin Eriksson, Amandine Mosnier, Floriane Albert, Virginie Mutez, Océane Brassard, Tugba Baysal, Mathilde Tardieu, Omran Allatif, Floriane Fusil, Thibault Andrieu, Denis Jullien, Valérie Dubois, Catherine Giannoli, Henri Gruffat, Marc Pallardy, François-Loïc Cosset, Audrey Nosbaum, Osami Kanagawa, Janet L. Maryanski, Daniel Yerly, Jean-François Nicolas, Marc Vocanson*

*Corresponding author. Email: marc.vocanson@inserm.fr

Published 19 March 2021, *Sci. Adv.* **7**, eabe0013 (2021)
DOI: 10.1126/sciadv.abe0013

This PDF file includes:

Tables S1 to S12
Figs. S1 to S17

	Biological investigations			
	CyTOF	TCRVβ	TCR sequencing	TCR transfectant stimulation assay
TEN-1		D0	-	-
TEN-2	-	D0	D0*	-
TEN-3	D0	D0	D0	D2
TEN-4	D0	D0	D0	-
TEN-5		D0	D0	-
TEN-6	-	D0	D0	-
TEN-7	D1	D0	D1	D2
TEN-8	-	D0	D1	-
TEN-9	D1	D0	D1	-
TEN-10	D0	D2	D0	D0 & D2
TEN-11	-	D0	D2	
TEN-12	-	-	D0	-
TEN-13	-	D0	D2	-
TEN-14	-	-	D1*	-
TEN-15	D1	D1	D1	D1
TEN-16	-	-	D1	-
TEN-17	D0	-	-	-
TEN-18	D0£	-	-	
MPE-1	-	-	D1	-
MPE-2	-	-	D1	-
MPE-3	-	D1	D1	-
MPE-4	-	D1	D1	-
MPE-5	-	D1	D1	-
MPE-6	-	-	D1	-
MPE-7	-	D1	D1	-
MPE-8	-	D1	-	-
MPE-9	D1	-	-	-
MPE-10	D1	-	-	-
MPE-11	D1	-	-	-
MPE-12	D1	-	-	-
MPE-13	D2	-	-	-
MPE-14	D1	-	-	-

Table S1. Sampling days and subsequent biological analysis.

Table describes the days at which samples were collected after patient's arrival to the hospital (all from day 0 to day 2), as well as the corresponding biological investigations performed on these samples.

*Blister and PBMC samples for TCR sequencing were collected as indicated in the table, except for TEN-2 and TEN-14, for which PBMC samples were performed at 1 day of interval.

£For patient TEN-18, the cytotoxic but not the lineage phenotype was excluded from the analysis due to a technical problem.

Antibodies and panel information				
**Isotope Channel**	**Antibody/reagent name**	**Clone**	**Source**	**Category**
191/193 Ir	DNA		Miltenyi	Cells isolation
194Pt	Viability		Miltenyi	Viability
89Y	CD45	HI30	Fluidigm	Lineage
142Nd	CD19	LT19	Miltenyi	Lineage
144Nd	TCR Vα14-Jα18	6B11	Miltenyi	Lineage
145Nd	CD11c	M54-27G12	Miltenyi	Lineage
148Nd	CD14	TUK4	Miltenyi	Lineage
150Nd	CD11b	M1/70.15.11.5	Miltenyi	Lineage
153Eu	CD45RA	T6D11	Miltenyi	Lineage
155Gd	CD8β	SIDI8BEE	Miltenyi	Lineage
158Gd	CCR7(CD197)	FR11-11EB	Miltenyi	Lineage
159Tb	TCR Vα7.2	REA179	Miltenyi	Lineage
164Dy	CD4	VIT4	Miltenyi	Lineage
166Er	NKp46	9 E2	Miltenyi	Lineage
168Er	TCR αβ	BW242/412	Miltenyi	Lineage
169Tm	CD8α	BW135/80	Miltenyi	Lineage
175Lu	TCRγδ	11F2	Miltenyi	Lineage
141Pr	CD56	HCD56	Miltenyi	Lineage/Activation
146Nd	CD107a	H4A3	Miltenyi	Activation
152Sm	CD27	M-T271	Miltenyi	Activation
163Di	CD57	HCD57	Miltenyi	Activation
167Er	CD38	REA572	Miltenyi	Activation
170Er	CD137	4B4-1	Miltenyi	Activation
171Yb	Annexin A1	74/3	Miltenyi	Activation
172Yb	CD253	RIK2.1	Miltenyi	Activation
174Yb	CD226	DX11	Miltenyi	Activation
147Sm	PERF	delta G9	Miltenyi	Cytotoxicity
149Sm	GzmB	REA226	Miltenyi	Cytotoxicity
151Eu	GzmA	REA162	Miltenyi	Cytotoxicity
161Dy	CD255	CARL-1	Miltenyi	Cytotoxicity
162Dy	GNLY	AF3138	Miltenyi	Cytotoxicity
154Sm	V-beta 13.2	H132	Beckman Coulter	TCR
156Gd	V-beta 7.2	ZIZOU4	Beckman Coulter	TCR
160Gd	V-beta 21.3	IG125	Beckman Coulter	TCR

Table S2. Antibodies and panel information.

A

Patient ID	Total templates	Productive templates	Productive fraction	Productive rearrangements	Productive clonality	TRB accounting for>0.5% total	
						N TRB sequences	Cum% of TRB repertoire
TEN-2	15156	12345	0.81	10051	0.03	1	3.65
TEN-3	9911	9083	0.92	2612	0.55	2	63.40
TEN-4	7602	5904	0.78	2261	0.17	16	29.97
TEN-5	951	718	0.76	452	0.06	18	22.98
TEN-6	27148	24951	0.92	2568	0.61	9	77.04
TEN-7	135218	109695	0.81	32357	0.17	7	14.26
TEN-8	91287	73280	0.80	56653	0.03	2	1.70
TEN-9	24344	23321	0.96	1745	0.69	6	85.44
TEN-10	115687	111243	0.96	4319	0.87	2	89.60
TEN-11	7408	5691	0.77	836	0.36	24	70.64
TEN-12	16159	12809	0.79	8121	0.09	5	11.66
TEN-13	2564	2163	0.84	1134	0.21	9	33.24
TEN-14	37,685	30113	0.80	18898	0.04	0	0
TEN-15	51687	30309	0.58	2111	0.59	11	69.94
TEN-16	15801	12384	0.78	4962	0.13	10	13.64
MPE-1	64906	53041	0.81	19223	0.08	2	1.70
MPE-2	211797	170222	0.80	68346	0.07	0	0
MPE-3	45946	36899	0.80	14225	0.09	2	2.39
MPE-4	7087	5775	0.81	2757	0.09	17	14.61
MPE-5	10537	8457	0.80	5139	0.06	8	6.83
MPE-6	5114	4280	0.84	2364	0.15	4	20.68
MPE-7	48944	39474	0.81	20772	0.07	3	2.38

B

Patient ID	Total templates	Productive templates	Productive fraction	Productive rearrangements	Productive clonality	TRB accounting for>0.5% total	
						N TRB sequences	Cum% of TCB repertoire
TEN-2	42212	33656	0.80	26806	0.02	1	0.65
TEN-3	25349	21559	0.85	12802	0.12	11	16.70
TEN-4	22380	16933	0.76	6559	0.16	9	18.68
TEN-5	97423	77785	0.80	38437	0.15	16	17.01
TEN-6	45198	36165	0.80	24135	0.11	11	16.86
TEN-7	68825	50224	0.73	21623	0.22	10	29.79
TEN-8	18785	15003	0.80	13198	0.01	1	0.55
TEN-9	857	676	0.79	471	0.06	14	22.63
TEN-10	5087	4021	0.79	3170	0.04	4	5.65
TEN-11	19359	15443	0.80	9465	0.08	4	8.01
TEN-12	108801	87610	0.81	71003	0.02	1	0.86
TEN-13	97529	80175	0.82	57810	0.03	1	0.81
TEN-14	20670	17286	0.84	11424	0.01	4	22.5
TEN-15	29410	24394	0.83	15989	0,1	6	15.21
TEN-16	61236	47672	0.78	19237	0.27	11	33.27
MPE-3	23185	18627	0.80	10726	0.12	11	15.74
MPE-4	6152	4815	0.78	4176	0.02	3	4.38
MPE-5	11093	8560	0.77	6762	0.06	5	10.68
MPE-6	80269	65498	0.82	50441	0.02	2	1.31
MPE-7	31855	25294	0.79	17336	0.04	2	1.54

Table S3: Raw parameters of TRBV repertoire analysis.

Genomic DNA extracted from blister and skin (**A**) or PBMC (**B**) samples from 15 TEN and 7 MPE patients were used for survey level deep sequencing of the TCRβ-chain, using ImmunoSEQ™ platform. Data were analyzed using ImmunoSEQ™ analyser toolset. Table describes raw parameters of TCR repertoire analysis.

Total template: the sum of templates for all rearrangements in the sample. Productive templates: the sum of templates for all productive rearrangements in the sample. Fraction productive: the fraction of productive templates among all templates. Productive rearrangement: the count of unique rearrangements in the sample that are in-frame and do not contain a stop codon. Productive rearrangements can produce a functional protein receptor. Productive clonality: a statistic for how much of the repertoire is made of up of expanded clones. Number and cumulative percentage of TRBV sequences, representing > 0.5% of total TRBV repertoire.

	MPE (n=7)	TEN (n=15)	Healthy donors (n=44)
**Mean productive clonality**	0.053	0.092	0.099
**Standard Deviation**	0.039	0.065	0.056
**Standard Error of Mean**	0.018	0.017	0.008
**t-test (vs healthy donors)**	ns	ns	

Table S4: Comparison of productive clonality in PBMC samples from TEN, MPE patients and healthy donors.

ns. Student t test (two-tailed).

Patient	V family	TRBV	TRBD	TRBJ	Templates	Amino-acid	CDR3 rearrangement	Productive frequency	Respective anti-Vβ mAb	% Vβ+ cells among CD3+CD8+ T cells as determined by FACs analysis
	TRBV09	TRBV09-01	TRBD02-01*01	TRBJ02-01*01	450	CASSVDLSGNEQFF	CTGAGCTCTCTGGAGCTGGGGGACTCAGCTTTGTATTTCTGTGCCAGCAGCGTAGATCTGTCGGGGAATGAGCAGTTCTTCGGGCCA	3.64	Vb 1	27.00
	TRBV06	TRBV06-06	unresolved	TRBJ02-01*01	28	CASSYSIHEQFF	CTCAGGCTGGAGTTGGCTGCTCCCTCCCAGACATCTGTGTACTTCTGTGCCAGCAGTTACTCAATCCATGAGCAGTTCTTCGGGCCA	0.23	Vb 13.6	2.55
TEN-2	TRBV02	TRBV02-01*01	TRBD02-01*01	TRBJ02-07*01	21	CASSEQLAGLSYEQYF	TCCACAAAGCTGGAGGACTCAGCCATGTACTTCTGTGCCAGCAGTGAACAACTAGCGGGGTTGTCCTACGAGCAGTACTTCGGGCCG	0.17	Vb 22	5.75
	TRBV06	TRBV06-05*01	unresolved	TRBJ01-02*01	19	CASSQRDGYGYTF	AGGCTGCTGTCGGCTGCTCCCTCCCAGACATCTGTGTACTTCTGTGCCAGCAGCCAAAGGGACGGATATGGCTACACCTTCGGTTCG	0.15	Vb 13.1	13.7
	TRBV06	TRBV06-05*01	TRBD01-01*01	TRBJ02-05*01	19	CASSYANTGWETQYF	CTGTCGGCTGCTCCCTCCCAGACATCTGTGTACTTCTGTGCCAGCAGTTACGCCAATACAGGGTGGGAGACCCAGTACTTCGGGCCA	0.15	Vb 13.1	
	TRBV11	TRBV11-02*02	unresolved	TRBJ02-01*01	5621	CASSPFRDSYNEQFF	CAGCCTGCAAAGCTTGAGGACTCGGCCGTGTATCTCTGTGCCAGCAGCCCCTTCCGTGACTCCTACAATGAGCAGTTCTTCGGGCCA	61.88	Vb 21.3	57.00
	TRBV19	TRBV19-01	TRBD01-01*01	TRBJ01-05*01	138	CATLDRYSNQPQHF	GTGACATCGGCCCAAAAGAACCCGACAGCTTTCTATCTCTGTGCCACCTTGGACAGATATAGCAATCAGCCCCAGCATTTTGGTGAT	1.52	Vb 17	1.02
TEN-3	TRBV19	TRBV19-01	TRBD02-01*02	TRBJ01-02*01	39	CASSEGSYGYTF	CTCACTGTGACATCGGCCCAAAAGAACCCGACAGCTTTCTATCTCTGTGCCAGTAGTGAGGGCAGCTATGGCTACACCTTCGGTTCG	0.43	Vb 17	
	TRBV09	TRBV09-01	unresolved	TRBJ02-01*01	17	CASSVSGQTSNEQFF	AGCTCTCTGGAGCTGGGGGACTCAGCTTTGTATTTCTGTGCCAGCAGTGTCAGCGGACAGACTAGCAATGAGCAGTTCTTCGGGCCA	0.19	Vb 1	3.61
	TRBV06	TRBV06-01*01	unresolved	TRBJ02-02*01	16	CASSEARGGSGELFF	GAGTCGGCTGCTCCCTCCCAGACATCTGTGTACTTCTGTGCCAGCAGTGAAGCCAGAGGGGGGTCCGGGGAGCTGTTTTTTGGAGAA	0.18	na	na
	TRBV05	TRBV05-06*01	TRBD02-01	TRBJ02-01*01	348	CASSFPSENEQFF	AATGTGAACGCCTTGTTGCTGGGGGACTCGGCCCTCTATCTCTGTGCCAGCAGCTTTCCTAGCGAAAATGAGCAGTTCTTCGGGCCA	5.89	Vb 5.2	2.75
	TRBV28	TRBV28-01*01	TRBD02-01	TRBJ02-07*01	248	CASLGTSSYEQYF	ATTCTGGAGTCCGCCAGCACCAACCAGACATCTATGTACCTCTGTGCCAGCCTGGGGACTAGCTCCTACGAGCAGTACTTCGGGCCG	4.20	Vb 3	24.2
TEN-4	TRBV30	TRBV30-01*01	TRBD01-01*01	TRBJ02-07*01	185	CAQGAREQYF	TTCATCCTGAGTTCTAAGAAGCTCCTTCTCAGTGACTCTGGCTTCTATCTCTGTGCCCAGGGGGCGCGGGAGCAGTACTTCGGGCCG	3.13	Vb 20	1.56
	TRBV29	TRBV29-01*01	unresolved	TRBJ02-03*01	180	CSVLQLTDTQYF	CTGACTGTGAGCAACATGAGCCCTGAAGACAGCAGCATATATCTCTGCAGCGTTCTACAACTCACAGATACGCAGTATTTTGGCCCA	3.05	Vb 4	3.33
	TRBV14	TRBV14-01*01	TRBD01-01*01	TRBJ02-07*01	167	CASSPSLTGSGEQYF	CAGCCTGCAGAACTGGAGGATTCTGGAGTTTATTTCTGTGCCAGCAGCCCATCTTTGACAGGGAGCGGCGAGCAGTACTTCGGGCCG	2.83	Vb 16	3.62
	TRBV15	TRBV15-01*01	TRBD02-01*01	TRBJ02-05*01	49	CATSRDVAGGGTQYF	CGCTCACCAGGCCTGGGGGACGCAGCCATGTACCTGTGTGCCACCAGCAGAGATGTAGCGGGGGGCGGGACCCAGTACTTCGGGCCA	6.82	na	na
	TRBV14	TRBV14-01*01	na	TRBJ02-07*01	18	CASSHLAQGGGEQYF	CAGCCTGCAGAACTGGAGGATTCTGGAGTTTATTTCTGTGCCAGCAGCCACTTGGCCCAGGGTGGGGGCGAGCAGTACTTCGGGCCG	2.51	Vb 16	1.88
TEN-5	TRBV06	TRBV06-05*01	TRBD02-01*01	TRBJ01-02*01	12	CASSPTQNGGGGYTF	CTGTCGGCTGCTCCCTCCCAGACATCTGTGTACTTCTGTGCCAGCAGTCCGACGCAAAACGGGGGGGGTGGCTACACCTTCGGTTCG	1.67	Vb 13.1	0.94
	TRBV10	TRBV10-03*01	na	TRBJ01-02*01	11	CAISGAAANYGYTF	CTGGAGTCCGCTACCAGCTCCCAGACATCTGTGTACTTCTGTGCCATCAGTGGAGCTGCAGCCAACTATGGCTACACCTTCGGTTCG	1.53	Vb 12	1.7
	TRBV04	TRBV04-03*01	TRBD02-01	TRBJ01-04*01	10	CASSQDLSDYEKLFF	CACACCCTGCAGCCAGAAGACTCGGCCCTGTATCTCTGCGCCAGCAGCCAAGATCTCTCGGACTATGAAAAACTGTTTTTTGGCAGT	1.39	Vb 7.2	9.15
	TRBV21	TRBV21-01*01	TRBD01-01*01	TRBJ01-04*01	8509	CASSDRGRGTNEKLFF	TCCACGGAGTCAGGGGACACAGCACTGTATTTCTGTGCCAGCAGCGACAGGGGAAGAGGTACTAATGAAAAACTGTTTTTTGGCAGT	34.10	na	na
	TRBV28	TRBV28-01*01	unresolved	TRBJ01-04*01	8503	CASSFGGPNEKLFF	CTGGAGTCCGCCAGCACCAACCAGACATCTATGTACCTCTGTGCCAGCAGTTTGGGGGGCCTAATGAAAAACTGTTTTTTGGCAGT	34.08	Vb 3	48.1
TEN-6	TRBV18	TRBV18-01*01	TRBD01-01*01	TRBJ01-02*01	695	CASSPGQGRSYGYTF	CAGCAGGTAGTGCGAGGAGATTCGGCAGCTTATTTCTGTGCCAGCTCACCCGGACAGGGAAGAAGCTATGGCTACACCTTCGGTTCG	2.79	Vb 18	3.26
	TRBV06	TRBV06-04	TRBD02-01	TRBJ01-02*01	508	CASSLEPGDSVYGYTF	TCTGCTGTACCCTCTCAGACATCTGTGTACTTCTGTGCCAGCAGCTTGGAACCTGGGGACTCGGTCTATGGCTACACCTTCGGTTCG	2.04	na	na
	TRBV03	unresolved	TRBD01-01*01	TRBJ01-03*01	297	CASSQERGRDTIYF	ATCAATTCCCTGGAGCTTGGTGACTCTGCTGTGTATTTCTGTGCCAGCAGCCAAGAAAGGGGAAGGGACACCATATATTTTGGAGAG	1.19	Vb 9	3.56
	TRBV02	TRBV02-01*01	TRBD02-01*01	TRBJ02-07*01	5168	CASSEVVAGASSYEQYF	ACAAAGCTGGAGGACTCAGCCATGTACTTCTGTGCCAGCAGTGAAGTGGTAGCGGGGGCTAGCTCCTACGAGCAGTACTTCGGGCCG	4.71	Vb 22	2.46
	TRBV05	TRBV05-05*01	TRBD01-01*01	TRBJ01-01*01	3889	CASSLNRDRSNTEAFF	GCCTTGTTGCTGGGGGACTCGGCCCTGTATCTCTGTGCCAGCAGCTTGAACAGGGACCGATCGAACACTGAAGCTTTCTTTGGACAA	3.55	Vb 5.3	3.6
TEN-7	TRBV06	unresolved	TRBD02-01*02	TRBJ02-01*01	3588	CASSPTSGSSNEQFF	GAGTCGGCTGCTCCCTCCCAAACATCTGTGTACTTCTGTGCCAGCAGCCCGACTAGCGGGAGTAGTAATGAGCAGTTCTTCGGGCCA	3.27	Vb 13.1; Vb 13.2; Vb 13.6	9.25
	TRBV06	TRBV06-05*01	TRBD02-01*01	TRBJ01-02*01	1168	CASSLGGEGYTF	CTCAGGCTGCTGTCGGCTGCTCCCTCCCAGACATCTGTGTACTTCTGTGCCAGCAGTCTCGGGGGAGAAGGCTACACCTTCGGTTCG	1.06	Vb 13.1	4.39
	TRBV04	TRBV04-01*01	TRBD02-01	TRBJ02-07*01	652	CASSQDELEQYF	CTTCACCTACACGCCCTGCAGCCAGAAGACTCAGCCCTGTATCTCTGCGCCAGCAGCCAAGATGAACTCGAGCAGTACTTCGGGCCG	0.59	Vb 7.1	1.25
	TRBV11	TRBV11-03*01	TRBD01-01*01	TRBJ01-05*01	775	CASSNPTGQGHSNQPQHF	GAGCTTGGGGACTCGGCCGTGTATCTCTGTGCCAGCAGCAACCCGACGGGACAGGGGCATAGCAATCAGCCCCAGCATTTTGGTGAT	1.06	na	na
	TRBV27	TRBV27-01*01	TRBD01-01*01	TRBJ02-03*01	471	CASRTGPDTQYF	CTGATCCTGGAGTCGCCCAGCCCCAACCAGACCTCTCTGTACTTCTGTGCCAGCAGGACAGGGCCAGATACGCAGTATTTTGGCCCA	0.64	Vb 14	1.2
TEN-8	TRBV04	TRBV04-03*01	na	TRBJ02-02*01	289	CASSQEGGGNTGELFF	ACCCTGCAGCCAGAAGACTCGGCCCTGTATCTCTGCGCCAGCAGCCAAGAAGGAGGGGGGAACACCGGGGAGCTGTTTTTTGGAGAA	0.39	Vb 7.2	5.76
	TRBV27	TRBV27-01*01	TRBD02-01	TRBJ02-07*01	197	CASFTSSYEQYF	CTGATCCTGGAGTCGCCCAGCCCCAACCAGACCTCTCTGTACTTCTGTGCCAGCTTTACTAGCTCCTACGAGCAGTACTTCGGGCCG	0.27	Vb 14	1.2
	TRBV04	TRBV04-03*01	TRBD01-01*01	TRBJ01-05*01	123	CASSQGAGSTNQPQHF	ACCCTGCAGCCAGAAGACTCGGCCCTGTATCTCTGCGCCAGCAGCCAAGGTGCAGGATCGACGAATCAGCCCCAGCATTTTGGTGAT	0.17	Vb 7.2	5.76
	TRBV02	TRBV02-01*01	TRBD02-01*02	TRBJ01-05*01	10334	CASSDVGRTSNQPQHF	TCCACAAAGCTGGAGGACTCAGCCATGTACTTCTGTGCCAGCAGTGATGTGGGGAGAACTAGCAATCAGCCCCAGCATTTTGGTGAT	44.31	Vb 22	35.9
	TRBV06	TRBV06	TRBD01-01*01	TRBJ01-01*01	7373	CASSYSDPRDMNTEAFF	GCTGCTCCCTCCCAAACATCTGTGTACTTCTGTGCCAGCAGTTACTCCGATCCGAGGGACATGAACACTGAAGCTTTCTTTGGACAA	31.62	Vb 13.1; Vb 13.2; Vb 13.6	42.26
TEN-9	TRBV21	TRBV21-01*01	TRBD01-01*01	TRBJ01-01*01	763	CASSKHPGLNTEAFF	CAGTCCACGGAGTCAGGGGACACAGCACTGTATCTCTGTGCCAGCAGCAAACACCCAGGGTTAAACACTGAAGCTTTCTTTGGACAA	3.27	na	na
	TRBV29	TRBV29-01*01	TRBD02-01*01	TRBJ01-02*01	737	CSVHSFGGASGYTF	GTGAGCAACATGAGCCCTGAAGACAGCAGCATATATCTCTGCAGCGTTCATTCCTTTGGGGGGGCAAGTGGCTACACCTTCGGTTCG	3.16	Vb 4	5.86
	TRBV24	TRBV24	na	TRBJ02-01*01	590	CATSPDPPADEQFF	CTAGAGTCTGCCATCCCCAACCAGACAGCTCTTTACTTCTGTGCCACCAGCCCGGACCCCCCGGCTGATGAGCAGTTCTTCGGGCCA	2.53	na	na
	TRBV28	TRBV28-01*01	TRBD02-01*01	TRBJ01-04*01	99110	CASSFGGPNEKLFF	CTGGAGTCCGCCAGCACCAACCAGACATCTATGTACCTCTGTGCCAGCAGTTTCGGGGGGCCTAATGAAAAACTGTTTTTTGGCAGT	89.09	Vb 3	93.9
	TRBV28	TRBV28-01*01	na	TRBJ02-02*01	566	CASSPGSNTGELFF	CTGGAGTCCGCCAGCACCAACCAGACATCTATGTACCTCTGTGCCAGCAGCCCTGGAAGCAACACCGGGGAGCTGTTTTTTGGAGAA	0.51	Vb 3	
TEN-10	TRBV04	TRBV04-03*01	TRBD01-01*01	TRBJ02-07*01	376	CASSQDLGPVEYEQYF	ACCCTGCAGCCAGAAGACTCGGCCCTGTATCTCTGCGCCAGCAGCCAAGATCTGGGGCCAGTAGAGTACGAGCAGTACTTCGGGCCG	0.34	Vb 7.2	0.88
	TRBV07	TRBV07-03*03	TRBD02-01	TRBJ02-02*01	374	RASSFTGTGASGELFF	CGCACAGAGCAGGGGGACTCAGCCGCGTATCTCCGTGCCAGCAGCTTCACCGGGACTGGGGCGTCTGGGGAGCTGTTTTTTGGAGAA	0.34	na	na
	TRBV19	TRBV19-01	na	TRBJ02-05*01	238	CASSIRGGETQYF	ACTGTGACATCGGCCCAAAAGAACCCGACAGCTTTCTATCTCTGTGCCAGTAGTATTAGGGGCGGGGAGACCCAGTACTTCGGGCCA	0.21	Vb 17	3.18
	TRBV12	TRBV12	TRBD01-01*01	TRBJ02-05*01	760	CASSLQEETQYF	CTGAAGATCCAGCCCTCAGAACCCAGGGACTCAGCTGTGTACTTCTGTGCCAGCAGTTTGCAGGAAGAGACCCAGTACTTCGGGCCA	13.35	na	na
	TRBV05	TRBV05-01*01	unknown	TRBJ01-02*01	706	CASSLDDSGFYGYTF	AGCACCTTGGAGCTGGGGGACTCGGCCCTTTATCTTTGCGCCAGCAGCTTGGACGACAGCGGATTTTATGGCTACACCTTCGGTTCG	12.41	Vb 5.1	11.9
TEN-11	TRBV04	TRBV04-01*01	unknown	TRBJ01-01*01	379	CASSQGDTEAFF	CTTCACCTACACGCCCTGCAGCCAGAAGACTCAGCCCTGTATCTCTGCGCCAGCAGCCAAGGGGACACTGAAGCTTTCTTTGGACAA	6.66	Vb 7.1	6.04
	TRBV24	TRBV24	TRBD02-01*02	TRBJ02-01*01	316	CATEREWGNEQFF	TCCCTAGAGTCTGCCATCCCCAACCAGACAGCTCTTTACTTCTGTGCCACGGAGCGGGAGTGGGGAAATGAGCAGTTCTTCGGGCCA	5.55	na	na
	TRBV05	TRBV05-06*01	unknown	TRBJ01-05*01	194	CASSLGYQPQHF	CTGAATGTGAACGCCTTGTTGCTGGGGGACTCGGCCCTCTATCTCTGTGCCAGCAGCTTGGGCTATCAGCCCCAGCATTTTGGTGAT	3.41	Vb 5.2	0.086
	TRBV27	TRBV27-01*01	na	TRBJ01-02*01	997	CASSYHGAADGYTF	CTGGAGTCGCCCAGCCCCAACCAGACCTCTCTGTACTTCTGTGCCAGCAGTTACCACGGGGCTGCGGATGGCTACACCTTCGGTTCG	7.78	Vb 14	nd
	TRBV04	TRBV04-01*01	TRBD02-01*01	TRBJ02-02*01	187	CASTLSGGSTGELFF	CACGCCCTGCAGCCAGAAGACTCAGCCCTGTATCTCTGCGCCAGCACCCTTAGCGGGGGGTCCACCGGGGAGCTGTTTTTTGGAGAA	1.46	Vb 7.1	nd
TEN-12	TRBV07	TRBV07-08*01	TRBD01-01*01	TRBJ02-07*01	130	CASSLGQAYEQYF	AAGATCCAGCGCACACAGCAGGAGGACTCCGCCGTGTATCTCTGTGCCAGCAGCTTAGGCCAGGCCTACGAGCAGTACTTCGGGCCG	1.01	na	nd
	TRBV05	TRBV05-04*01	TRBD02-01*01	TRBJ02-07*01	107	CASSLGRGGSYEQYF	AACGCCTTGGAGCTGGACGACTCGGCCCTGTATCTCTGTGCCAGCAGCTTGGGTCGGGGGGGCTCCTACGAGCAGTACTTCGGGCCG	0.84	na	nd
	TRBV04	TRBV04-01*01	TRBD01-01*01	TRBJ01-02*01	73	CASSPRHQGINYGYTF	GCCCTGCAGCCAGAAGACTCAGCCCTGTATCTCTGCGCCAGCAGCCCCAGGCACCAGGGGATTAACTATGGCTACACCTTCGGTTCG	0.57	Vb 7.1	nd
	TRBV04	TRBV04-01*01	TRBD01-01*01	TRBJ01-02*01	565	CASSQDSLGTGSGYTF	GCCCTGCAGCCAGAAGACTCAGCCCTGTATCTCTGCGCCAGCAGCCAAGATAGTTTAGGTACAGGGTCTGGCTACACCTTCGGTTCG	26.11	Vb 7.1	18.1
	TRBV28	TRBV28-01*01	TRBD02-01*02	TRBJ02-01*01	42	CASSHFPGLAGEEQFF	TCCGCCAGCACCAACCAGACATCTATGTACCTCTGTGCCAGCAGTCACTTCCCGGGACTAGCGGGAGAGGAGCAGTTCTTCGGGCCA	1.94	Vb 3	8.84
TEN-13	TRBV24	TRBV24	TRBD02-01	TRBJ01-02*01	22	CATSDIDNYGYTF	TCCCTAGAGTCTGCCATCCCCAACCAGACAGCTCTTTACTTCTGTGCCACCAGTGATATAGATAACTATGGCTACACCTTCGGTTCG	1.02	na	na
	TRBV11	TRBV11-03*01	TRBD02-01	TRBJ01-01*01	21	CASSVSLATEAFF	AAGATCCAGCCTGCAGAGCTTGGGGACTCGGCCGTGTATCTCTGTGCCAGCAGCGTAAGTTTAGCAACGGAAGCTTTCTTTGGACAA	0.97	na	na
	TRBV04	TRBV04-03*01	TRBD01-01*01	TRBJ01-02*01	19	CASSPNTGVSGYTF	CTACACACCCTGCAGCCAGAAGACTCGGCCCTGTATCTCTGCGCCAGCAGTCCTAACACAGGGGTCTCTGGCTACACCTTCGGTTCG	0.88	Vb 7.2	0.59
	TRBV07	TRBV07-03*01	TRBD01-01*01	TRBJ01-01*01	139	CASSWTENTEAFF	AAGATCCAGCGCACAGAGCGGGGGGACTCAGCCGTGTATCTCTGTGCCAGCAGCTGGACAGAGAACACTGAAGCTTTCTTTGGACAA	0.46	na	na
	TRBV10	TRBV10-03*01	unknown	TRBJ01-01*01	95	CAISESGAGLNTEAFF	TCCGCTACCAGCTCCCAGACATCTGTGTACTTCTGTGCCATCAGTGAGTCCGGGGCAGGGCTGAACACTGAAGCTTTCTTTGGACAA	0.32	Vb 12	na
TEN-14	TRBV29	TRBV29-01*01	unknown	TRBJ02-02*01	94	CSVGGANTGELFF	ACTGTGAGCAACATGAGCCCTGAAGACAGCAGCATATATCTCTGCAGCGTTGGTGGGGCGAACACCGGGGAGCTGTTTTTTGGAGAA	0.31	Vb 4	na
	TRBV29	TRBV29-01*01	TRBD01-01*01	TRBJ02-02*01	86	CSVGATNTGELFF	ACTGTGAGCAACATGAGCCCTGAAGACAGCAGCATATATCTCTGCAGCGTTGGGGCCACGAACACCGGGGAGCTGTTTTTTGGAGAA	0.29	Vb 4	na
	TRBV27	TRBV27-01*01	TRBD01-01*01	TRBJ01-05*01	80	CASSLRTGHYQPQHF	GAGTCGCCCAGCCCCAACCAGACCTCTCTGTACTTCTGTGCCAGCAGTTTAAGGACGGGACACTATCAGCCCCAGCATTTTGGTGAT	0.27	Vb 14	na
	TRBV27	TRBV27-01*01	TRBD02-01*01	TRBJ02-01*01	12643	CASSLAGGLGEQFF	CTGGAGTCGCCCAGCCCCAACCAGACCTCTCTGTACTTCTGTGCCAGCAGTCTAGCGGGGGGGCTGGGTGAGCAGTTCTTCGGGCCA	41.71	Vb 14	27.2
	TRBV18	TRBV18-01*01	TRBD01-01*01	TRBJ01-05*01	3635	CASSGGRDQPQHF	AGGATCCAGCAGGTAGTGCGAGGAGATTCGGCAGCTTATTTCTGTGCCAGCTCAGGGGGCAGGGATCAGCCCCAGCATTTTGGTGAT	11.99	Vb 18	0.65
TEN-15	TRBV06	TRBV06-05*01	unknown	TRBJ02-03*01	3463	CASSFSGSDTQYF	AGGCTGCTGTCGGCTGCTCCCTCCCAGACATCTGTGTACTTCTGTGCCAGCAGTTTCTCGGGTTCGGATACGCAGTATTTTGGCCCA	11.43	Vb 13.1	16.4
	TRBV20	TRBV20-01*01	TRBD02-01	TRBJ01-02*01	1893	CSALGPYGYTF	ACTCTGACAGTGACCAGTGCCCATCCTGAAGACAGCAGCTTCTACATCTGCAGTGCTCTCGGTCCCTATGGCTACACCTTCGGTTCG	6.25	Vb 2	4.72
	TRBV20	TRBV20	TRBD01-01*01	TRBJ01-05*01	648	CSAGRGRDQPQHF	ACAGTGACCAGTGCCCATCCTGAAGACAGCAGCTTCTACATCTGCAGTGCAGGCAGGGGAAGGGATCAGCCCCAGCATTTTGGTGAT	1.40	Vb 2	4.72
	TRBV09	TRBV09-01	TRBD01-01*01	TRBJ01-02*01	648	CASSGRTASYGYTF	CTGAGCTCTCTGGAGCTGGGGGACTCAGCTTTGTATTTCTGTGCCAGCAGCGGACGGACAGCAAGTTATGGCTACACCTTCGGTTCG	5.23	Vb 1	na
	TRBV10	TRBV10-03*01	TRBD01-01*01	TRBJ01-05*01	422	CAIGTGDSNQPQHF	CTGGAGTCCGCTACCAGCTCCCAGACATCTGTGTACTTCTGTGCCATCGGGACAGGGGATAGCAATCAGCCCCAGCATTTTGGTGAT	3.41	Vb 12	na
TEN-16	TRBV29	TRBV29-01*01	TRBD02-01*02	TRBJ02-01*01	213	CSATYLAGVHNEQFF	AGCAACATGAGCCCTGAAGACAGCAGCATATATCTCTGCAGCGCCACCTATTTAGCGGGAGTTCACAATGAGCAGTTCTTCGGGCCA	1.72	Vb 4	na
	TRBV12	TRBV12	TRBD01-01*01	TRBJ01-01*01	100	CASSPNDGQGDEAFF	CAGCCCTCAGAACCCAGGGACTCAGCTGTGTACTTCTGTGCCAGCAGTCCGAATGACGGACAGGGGGATGAAGCTTTCTTTGGACAA	0.81	Vb 8	na
	TRBV30	TRBV30-01	TRBD01-01*01	TRBJ01-02*01	87	CAWMPLISEKYRGLNYGYTF	AGTGACTCTGGCTTCTATCTCTGTGCCTGGATGCCTCTAATTTCCGAGAAATACAGGGGGCTTAACTATGGCTACACCTTCGGTTCG	0.70	Vb 20	na

Table S5: Productive frequency of the 5 most common TCR-β clonotypes found in blister TEN samples, and comparison to the frequency of respective TCR Vβ chains detected by FACS.

Genomic DNA extracted from blister TEN samples were used for survey level deep sequencing of the TCRβ chain, using ImmunoSEQ™ platform. TRBV repertoire data were compared to the frequency of respective TCR Vβ+ cells detected among CD3+CD8+ T cells by FACS. Of note, the anti-Vβ mAb nomenclature is distinct from the corresponding TRBV nomenclature. Information for Vb family, TRBV, TRBD, TRBJ, template number, amino acid and CDR3 rearrangement are also provided.

Vβ family: the identified V Gene Family that contributed to a specific rearrangement. *TRBV:* a concise string identifying the most specific V Gene family, gene or allele identified during annotation. *TRBD:* a concise string identifying the most specific D Gene family, gene or allele identified during annotation. *TRBJ:* a concise string identifying the most specific J Gene family, gene or allele identified during annotation. *Templates:* the total number of templates for a specific rearrangement in the sample. *CDR3 rearrangement:* a particular nucleotide sequence generated through V(D)J recombination. Only productive rearrangements are shown. Productive rearrangements are in-frame, do not contain a stop codon and can produce a functional protein receptor. *Productive frequency:* the frequency of a specific « productive rearrangement » among all productive rearrangements within a sample, calculated as the templates for a specific rearrangement divided by the sum of productive templates for a sample.

Patient	Locus	Sorting Strategy	V family	TRAV/TRBV	TRAJ/TRBJ	Templates	Amino-acid	CDR3 rearrangement	Productive Frequency
			TRAV19	TRAV19-01*01	TRAJ30-01*01	1022	CALSVYMNRDDKIIF	CTCACAAGTCGTGGACTCAGCAGTATACTTCTGTGCTCTGAGTGTGTATATGAACAGAGATGACAAGATCATCTTTGGAAAA	51,93
		BLISTERS	TRAV19	TRAV19-01*01	TRAJ29-01*01	951	CALSEAWGNTPLVF	AGCCTCACAAGTCGTGGACTCAGCAGTATACTTCTGTGCTCTGAGTGAGGCGTGGGGAAACACACCTCTTGTCTTTGGAAAG	47,88
TEN-9	TRA	-	TRAV16	TRAV12-02	TRAJ54-01*01	2	CALRGPAAGNKLTF	ATTTGCTCAAGAGGAAGACTCAGCCATGTATTACTGTGCTCTAAGGGGCCCCGCTGCAGGCAACAAGCTAACTTTTGGAGGA	0,08
		CD8+Vβ22+	TRAV12	TRAV17-01*01	TRAJ06-01*01	1	CAVTSQGAQKLVF	CAGAGACTCCCAGCCCAGTGATTCAGCCACCTACCTCTGTGCCGTGACTAGCCAGGGAGCCCAGAAGCTGGTATTTGGCCAA	0,05
			TRAV17	TRAV16-01*01	TRAJ17-01*01	1	CATDAGAGGSYIPTF	TTCCCGGGCAGCAGACACTGCTTCTTACTTCTGTGCTACGGACGCGGGGGCAGGAGGAAGCTACATACCTACATTTGGAAGA	0,03
			TRAV19	TRAV19-01*01	TRAJ30-01*01	128	CALSVYMNRDDKIIF	CTCACAAGTCGTGGACTCAGCAGTATACTTCTGTGCTCTGAGTGTGTATATGAACAGAGATGACAAGATCATCTTTGGAAAA	49,41
		BLISTERS	TRAV19	TRAV19-01*01	TRAJ29-01*01	112	CALSEAWGNTPLVF	AGCCTCACAAGTCGTGGACTCAGCAGTATACTTCTGTGCTCTGAGTGAGGCGTGGGGAAACACACCTCTTGTCTTTGGAAAG	42,88
TEN-9	TRA	-	TRAV26	TRAV26-02	TRAJ34-01*01	4	CILLNTDKLIF	GATCCTGCACCGTGCTACCTTGAGAGATGCTGCTGTGTACTACTGCATCCTCCTCAACACCGACAAGCTCATCTTTGGGACT	1,7
		CD8+Vβ13.2+	TRAV17	TRAV17-01*01	TRAJ06-01*01	3	CATDAGAGGSYIPTF	TTCCCGGGCAGCAGACACTGCTTCTTACTTCTGTGCTACGGACGCGGGGGCAGGAGGAAGCTACATACCTACATTTGGAAGA	1,25
			TRAV19	TRAV19-01*01	TRAJ54-01*01	2	CALRPVTPQGAQKLVF	ACAAGTCGTGGACTCAGCAGTATACTTCTGTGCTCTGCGCCCCGTTACCCCTCAGGGAGCCCAGAAGCTGGTATTTGGCCAA	0,57
			TRBV02	TRBV02-01*01	TRBJ01-05*01	136	CASSDVGRTSNQPQHF	TCCACAAAGCTGGAGGACTCAGCCATGTACTTCTGTGCCAGCAGTGATGTGGGGAGAACTAGCAATCAGCCCCAGCATTTTGGTGAT	56,43
		BLISTERS	TRBV06	TRBV06	TRBJ01-01*01	77	CASSYSDPRDMNTEAFF	GCTGCTCCCTCCCAAACATCTGTGTACTTCTGTGCCAGCAGTTACTCCGATCCGAGGGACATGAACACTGAAGCTTTCTTTGGACAA	31,9
TEN-9	TRB	-	TRBV03	TRBV03	TRBJ02-02*01	1	CASSTRRSGLTGELFF	TCCCTGGAGCTTGGTGACTCTGCTGTGTATTTCTGTGCCAGCAGCACGAGACGTTCAGGCTTGACCGGGGAGCTGTTTTTTGGAGAA	0,0041
		CD8+Vβ22+	TRBV09	TRBV09-01	TRBJ01-04*01	1	CASSPRQATNEKLFF	AGCTCTCTGGAGCTGGGGGACTCAGCTTTGTATTTCTGTGCCAGCAGCCCGAGACAGGCAACTAATGAAAAACTGTTTTTTGGCAGT	0,0041
			TRBV25	TRBV25-01*01	TRBJ02-06*01	1	CASSYTGQSRANVLTF	TCTGCCAGGCCCTCACATACCTCTCAGTACCTCTGTGCCAGCAGTTACACGGGACAAAGTCGGGCCAACGTCCTGACTTTCGGGGCC	0,0041
			TRBV02	TRBV02-01*01	TRBJ01-05*01	1231	CASSDVGRTSNQPQHF	TCCACAAAGCTGGAGGACTCAGCCATGTACTTCTGTGCCAGCAGTGATGTGGGGAGAACTAGCAATCAGCCCCAGCATTTTGGTGAT	59,4
		BLISTERS	TRBV06	TRBV06	TRBJ01-01*01	810	CASSYSDPRDMNTEAFF	GCTGCTCCCTCCCAAACATCTGTGTACTTCTGTGCCAGCAGTTACTCCGATCCGAGGGACATGAACACTGAAGCTTTCTTTGGACAA	39,1
TEN-9	TRB	-	TRBV02	TRBV02-01*01	TRBJ01-05*01	2	CASSDVGRTSNQPQHF	GTCCACAAAGCTGGAGGACTCAGCCATGTACTTCTGTGCCAGCAGTGATGTGGGGAGAACTAGCAATCAGCCCCAGCATTTTGGTGAT	9,65E-04
		CD8+Vβ13.2+	TRBV06	TRBV06	TRBJ01-01*01	2	CASSYSDPRDMNTEAFF	GGCTGCTCCCTCCAAACATCTGTGTACTTCTGTGCCAGCAGTTACTCCGATCCGAGGGACATGAACACTGAAGCTTTCTTTGGACAA	9,65E-04
			TRBV22	TRBV22-01*01	TRBJ01-02*01	2	CPGRALGKRKTYGYTF	TTGGCCCACACCAGCCAAACAGCTTTGTACTTCTGTCCTGGGAGGGCCCTCGGAAAGCGAAAGACCTATGGCTACACCTTCGGTTCG	9,65E-04

Table S6. The two dominant TCRVβ cells isolated from TEN-9 blisters represent a single clone, which has rearranged 2 functional TRBV genes, as well as 2 functional TRAV genes.

Dominant CD8+TCRVβ13.2+ and CD8+TCRVβ22+ cells were isolated from blister cells of patient TEN-9. Genomic DNA was extracted and used for survey level deep sequencing of the TCRα-chain and TCRβ-chain, using ImmunoSEQ™ platform. Information for Vβ/Vα family, TRB/AV, TRB/AD, TRB/AJ, amino acid and CDR3 rearrangement and productive frequency are provided.

CD8+TCRVβ13.2+ and CD8+TCRVβ22+ cells (expressed at similar percentages in skin lesions (**Figure 3**)) were associated with the same pair of overexpressed Vα chains (TCRVA19-01*01) but with distinct TRAJ segments (respectively TRAJ30-01*01 and TRAJ29-01*01). In parallel to the 2 TRAV genes, 2 functional TRBV genes (TRBV02-01*01 and TRBV06) were also detected, indicating that CD8+TCRVβ13.2+ and CD8+TCRVβ22+ cells were derived from the same unusual T cell clone.

Patient	V family	TRBV	TRBD	TRBJ	Templates	Amino-acid	CDR3 rearrangement	Productive frequency	Respective anti-Vβ mAb	% Vβ+ cells among CD3+CD8+ T cells as determined by FACs analysis
	TRBV05	TRBV05-06*01	unresolved	TRBJ01-01*01	499	CASSWVQLMNTEAFF	AACGCCTTGTTGCTGGGGGACTCGGCCCTCTATCTCTGTGCCAGCAGTTGGGTCCAGCTAATGAACACTGAAGCTTTCTTTGGACAA	3.65	Vb 1	na
	TRBV04	TRBV04-01*01	unresolved	TRBJ01-02*01	404	CASSQDRGSYYGYTF	CACGCCCTGCAGCCAGAAGACTCAGCCCTGTATCTCTGCGCCAGCAGCCAAGATCGGGGCAGTTACTATGGCTACACCTTCGGTTCG	0.23	Vb 13.6	na
MPE-1	TRBV03	unresolved	TRBD01-01*01	TRBJ01-04*01	253	CASSQLSGQINEKLFF	TCCCTGGAGCTTGGTGACTCTGCTGTGTATTTCTGTGCCAGCAGCCAATTATCTGGTCAGATTAATGAAAAACTGTTTTTTGGCAGT	0.17	Vb 22	na
	TRBV07	TRBV07-06*01	TRBD01-01*01	TRBJ02-02*01	246	CASSLVQGAGELFF	ATCCAGCGCACAGAGCAGCGGGACTCGGCCATGTATCGCTGTGCCAGCAGCTTAGTACAGGGGGCCGGGGAGCTGTTTTTTGGAGAA	0.15	Vb 13.1	na
	TRBV09	TRBV09-01	TRBD01-01*01	TRBJ02-07*01	232	CASSVGHSTSYEQYF	AGCTCTCTGGAGCTGGGGGACTCAGCTTTGTATTTCTGTGCCAGCAGCGTAGGGCACAGCACCTCCTACGAGCAGTACTTCGGGCCG	0.15	Vb 13.1	na
	TRBV27	TRBV27-01*01	TRBD02-01	TRBJ01-06*01	660	CASTPAGDYNSPLHF	GAGTCGCCCAGCCCCAACCAGACCTCTCTGTACTTCTGTGCCAGCACCCCGGCGGGCGACTATAATTCACCCCTCCACTTTGGGAAC	0.39	Vb 14	na
	TRBV04	TRBV04-03*01	TRBD01-01*01	TRBJ02-02*01	654	CASSPTRGYAGELFF	CACACCCTGCAGCCAGAAGACTCGGCCCTGTATCTCTGCGCCAGCAGCCCGACCAGGGGATATGCCGGGGAGCTGTTTTTTGGAGAA	0.38	Vb 7.2	na
MPE-2	TRBV11	TRBV11-02*02	TRBD01-01*01	TRBJ01-02*01	452	CASSTAGTGYGYTF	ATCCAGCCTGCAAAGCTTGAGGACTCGGCCGTGTATCTCTGTGCCAGCAGCACAGCCGGGACAGGGTATGGCTACACCTTCGGTTCG	0.27	Vb 21.3	na
	TRBV03	unresolved	TRBD01-01*01	TRBJ01-02*01	399	CASSQGLKVGATNYGYTF	GAGCTTGGTGACTCTGCTGTGTATTTCTGTGCCAGCAGCCAAGGTTTAAAAGTAGGGGCAACTAACTATGGCTACACCTTCGGTTCG	0.23	unknown	na
	TRBV02	TRBV02-01*01	TRBD02-01*01	TRBJ01-02*01	334	CASSEARGSYGYTF	ATCCGGTCCACAAAGCTGGAGGACTCAGCCATGTACTTCTGTGCCAGCAGTGAAGCGCGGGGAAGCTATGGCTACACCTTCGGTTCG	0.20	Vb 22	na
	TRBV05	TRBV05-04*01	TRBD01-01*01	TRBJ01-02*01	547	CASSLGWADYGYTF	GTGAACGCCTTGGAGCTGGACGACTCGGCCCTGTATCTCTGTGCCAGCAGCTTGGGGTGGGCTGACTATGGCTACACCTTCGGTTCG	1.48	unknown	na
	TRBV04	TRBV04-01*01	unknown	TRBJ01-02*01	334	CASRSEPAPNYGYTF	CACGCCCTGCAGCCAGAAGACTCAGCCCTGTATCTCTGCGCCAGCAGGTCAGAGCCGGCCCCTAACTATGGCTACACCTTCGGTTCG	0.90	Vb 7.1	6.36
MPE-3	TRBV07	TRBV07-02*01	TRBD02-01	TRBJ01-02*01	175	CASSLSLGYTF	ACTCTGACGATCCAGCGCACACAGCAGGAGGACTCGGCCGTGTATCTCTGTGCCAGCAGCTTAAGCCTTGGCTACACCTTCGGTTCG	0.47	unknown	na
	TRBV07	TRBV07-09	TRBD01-01*01	TRBJ01-02*01	137	CASSPPQGARGIYGYTF	ACAGAGCAGGGGGACTCGGCCATGTATCTCTGTGCCAGCAGCCCACCACAGGGGGCGCGGGGGATCTATGGCTACACCTTCGGTTCG	0.37	unknown	na
	TRBV20	TRBV20	unknown	TRBJ02-02*01	136	CSARPGQNTGELFF	GTGACCAGTGCCCATCCTGAAGACAGCAGCTTCTACATCTGCAGTGCTAGACCGGGCCAGAACACCGGGGAGCTGTTTTTTGGAGAA	0.37	unknown	na
	TRBV24	TRBV24	unknown	TRBJ02-04*01	106	CATSGPMLIAKNIQYF	TCTGCCATCCCCAACCAGACAGCTCTTTACTTCTGTGCCACCAGTGGCCCAATGTTAATAGCCAAAAACATTCAGTACTTCGGCGCC	1.84	unknown	na
	TRBV30	TRBV30-01*01	unknown	TRBJ01-01*01	90	CAWSVLRRRPDAFF	TCTAAGAAGCTCCTTCTCAGTGACTCTGGCTTCTATCTCTGTGCCTGGAGTGTACTGAGACGCAGACCTGATGCTTTCTTTGGACAA	1.56	Vb 20	6.07
MPE-4	TRBV09	TRBV09-01	unknown	TRBJ01-01*01	89	CASSEWTVSEAFF	AACCTGAGCTCTCTGGAGCTGGGGGACTCAGCTTTGTATTTCTGTGCCAGCAGCGAGTGGACCGTAAGCGAAGCTTTCTTTGGACAA	1.54	Vb 1	12.5
	TRBV05	TRBV05-04*01	TRBD02-01	TRBJ02-03*01	77	CASSPQGLAIPGDTQYF	TTGGAGCTGGACGACTCGGCCCTGTATCTCTGTGCCAGCAGCCCCCAGGGCCTAGCGATACCAGGGGATACGCAGTATTTTGGCCCA	1.33	unknown	na
	TRBV19	TRBV19-01	TRBD02-01	TRBJ02-07*01	57	CATHASSSYEQYF	ACTGTGACATCGGCCCAAAAGAACCCGACAGCTTTCTATCTCTGTGCCACCCATGCGTCAAGTTCCTACGAGCAGTACTTCGGGCCG	0.99	Vb 17	12.2
	TRBV02	TRBV02-01*01	TRBD01-01*01	TRBJ01-05*01	162	CASSPLRQGIASSNQPQHF	CTGGAGGACTCAGCCATGTACTTCTGTGCCAGCAGTCCCCTTAGACAGGGCATCGCCTCTAGCAATCAGCCCCAGCATTTTGGTGAT	1.92	Vb 22	5.68
	TRBV19	TRBV19-01	TRBD02-01*02	TRBJ02-03*01	73	CASSIGLAGLTDTQYF	TCGGCCCAAAAGAACCCGACAGCTTTCTATCTCTGTGCCAGTAGTATAGGCCTAGCGGGACTCACAGATACGCAGTATTTTGGCCCA	0.86	Vb 17	5.07
MPE-5	TRBV09	TRBV09-01	TRBD02-01	TRBJ02-01*01	71	CASSVDPGPSGRNEQFF	CTGGAGCTGGGGGACTCAGCTTTGTATTTCTGTGCCAGCAGCGTAGATCCAGGGCCTAGCGGGCGTAATGAGCAGTTCTTCGGGCCA	0.84	Vb 1	2.1
	TRBV07	TRBV07-06*01	TRBD02-01	TRBJ01-02*01	69	CASSLGEYYGYTF	ACGATCCAGCGCACAGAGCAGCGGGACTCGGCCATGTATCGCTGTGCCAGCAGCTTAGGCGAGTACTATGGCTACACCTTCGGTTCG	0.82	unknown	na
	TRBV02	TRBV02-01*01	unknown	TRBJ01-03*01	64	CASSPMGISGNTIYF	CGGTCCACAAAGCTGGAGGACTCAGCCATGTACTTCTGTGCCAGCAGTCCCATGGGGATCTCTGGAAACACCATATATTTTGGAGAG	0.76	Vb 22	5.68
	TRBV07	TRBV07-03*01	TRBD02-01*02	TRBJ01-03*01	771	CASTLRKAGGSGNTIYF	ACAGAGCGGGGGGACTCAGCCGTGTATCTCTGTGCCAGCACCCTCCGAAAGGCGGGAGGGTCTGGAAACACCATATATTTTGGAGAG	18.01	unknown	na
	TRBV28	TRBV28-01*01	TRBD01-01*01	TRBJ01-01*01	68	CASSSVFRTGATEAFF	TCCGCCAGCACCAACCAGACATCTATGTACCTCTGTGCCAGCAGTTCGGTGTTCAGGACAGGGGCCACTGAAGCTTTCTTTGGACAA	1.59	Vb 3	na
MPE-6	TRBV25	TRBV25-01*01	TRBD02-01*01	TRBJ01-01*01	24	CASTRPGSNTEAFF	CTGGAGTCTGCCAGGCCCTCACATACCTCTCAGTACCTCTGTGCCAGCACGAGACCGGGGTCGAACACTGAAGCTTTCTTTGGACAA	0.56	Vb 11	na
	TRBV07	TRBV07-09	TRBD02-01*02	TRBJ02-01*01	22	CASSLALLRRDVYNEQFF	GAGCAGGGGGACTCGGCCATGTATCTCTGTGCCAGCAGCTTAGCTCTTTTGCGGCGGGATGTCTACAATGAGCAGTTCTTCGGGCCA	0.51	unknown	na
	TRBV09	TRBV09-01	TRBD02-01	TRBJ01-05*01	21	CASSEDGGLNQPQHF	AGCTCTCTGGAGCTGGGGGACTCAGCTTTGTATTTCTGTGCCAGCAGCGAGGACGGGGGACTGAATCAGCCCCAGCATTTTGGTGAT	0.49	Vb 1	na
	TRBV06	TRBV06-01*01	TRBD02-01*01	TRBJ01-03*01	489	CASSAFLTGSIYF	AGGCTGGAGTCGGCTGCTCCCTCCCAGACATCTGTGTACTTCTGTGCCAGCAGTGCATTTCTTACGGGGTCCATATATTTTGGAGAG	1.24	unknown	na
	TRBV03	TRBV03	TRBD02-01	TRBJ02-02*01	243	CASNQGTRTGELFF	ATCAATTCCCTGGAGCTTGGTGACTCTGCTGTGTATTTCTGTGCCAGCAACCAAGGGACTCGCACCGGGGAGCTGTTTTTTGGAGAA	0.62	unknown	na
MPE-7	TRBV15	TRBV15-01*01	TRBD01-01*01	TRBJ01-01*01	207	CATSRDRQEAFF	CTTGACATCCGCTCACCAGGCCTGGGGGACGCAGCCATGTACCTGTGTGCCACCAGCAGAGATCGACAGGAAGCTTTCTTTGGACAA	0.52	unknown	na
	TRBV05	TRBV05-03	TRBD01-01*01	TRBJ01-03*01	171	CARSRGIRSGNTIYF	AGTGCCTTGGAGCTGGGGGACTCGGCCCTGTATCTCTGTGCCAGAAGCAGGGGAATTAGGTCTGGAAACACCATATATTTTGGAGAG	0.43	unknown	na
	TRBV11	TRBV11-03*01	TRBD02-01*02	TRBJ02-07*01	132	CASSLTEAYEQYF	AAGATCCAGCCTGCAGAGCTTGGGGACTCGGCCGTGTATCTCTGTGCCAGCAGCTTAACGGAGGCCTACGAGCAGTACTTCGGGCCG	0.33	unknown	na

Table S7: Comparison of the productive frequency of the 5 most common TCR-β clonotypes found in skin MPE samples, and the frequency of respective TCR-Vβ chains detected by FACS.

Genomic DNA extracted from skin MPE samples was used for survey level deep sequencing of the TCRβ-chain, using ImmunoSEQ^{TM} platform. The TRBV repertoire and TCR-Vβ FACS analysis is described in the footnotes of Table S5.

Patient	V family	TRBV	TRBD	TRBJ	Templates	Amino-acid	CDR3 rearrangement	Productive frequency	Respective anti-Vβ mAb	% Vβ+ cells among CD3+CD8+ T cells as determined by FACs analysis
	TRBV06	TRBV06-06	na	TRBJ02-01*01	218	CASSYSIHEQFF	CTCAGGCTGGAGTTGGCTGCTCCCTCCCAGACATCTGTGTACTTCTGTGCCAGCAGTTACTCAATCCATGAGCAGTTCTTCGGGCCA	0.65	Vb 13.6	na
	TRBV09	TRBV09-01	TRBD02-01*01	TRBJ02-01*01	144	CASSVDLSGNEQFF	CTGAGCTCTCTGGAGCTGGGGGACTCAGCTTTGTATTTCTGTGCCAGCAGCGTAGATCTGTCGGGGAATGAGCAGTTCTTCGGGCCA	0.43	Vb 1	na
TEN-2	TRBV06	TRBV06-05*01	TRBD01-01*01	TRBJ02-05*01	117	CASSYANTGWETQYF	CTGTCGGCTGCTCCCTCCCAGACATCTGTGTACTTCTGTGCCAGCAGTTACGCCAATACAGGGTGGGAGACCCAGTACTTCGGGCCA	0.35	Vb 13.1	na
	TRBV05	TRBV05-01*01	na	TRBJ02-02*01	99	CASSLYTGELFF	ATGAATGTGAGCACCTTGGAGCTGGGGGACTCGGCCCTTTATCTTTGCGCCAGCAGCTTGTACACCGGGGAGCTGTTTTTTGGAGAA	0.29	Vb 5.1	na
	TRBV06	TRBV06-05*01	TRBD01-01*01	TRBJ01-02*01	94	CASSYSGQGIYGYTF	CTGTCGGCTGCTCCCTCCCAGACATCTGTGTACTTCTGTGCCAGCAGTTACTCTGGGCAGGGGATCTATGGCTACACCTTCGGTTCG	0.28	Vb 13.1	na
	TRBV11	TRBV11-02*02	na	TRBJ02-01*01	1094	CASSPFRDSYNEQFF	CAGCCTGCAAAGCTTGAGGACTCGGCCGTGTATCTCTGTGCCAGCAGCCCCTTCCGTGACTCCTACAATGAGCAGTTCTTCGGGCCA	5.07	Vb 21.3	6.54
	TRBV27	TRBV27-01*01	TRBD01-01*01	TRBJ02-07*01	553	CASRSLQGARTTFLYEQYF	CCCAACCAGACCTCTCTGTACTTCTGTGCCAGCAGATCCTTACAGGGGGCGCGGACGACATTTCTCTACGAGCAGTACTTCGGGCCG	2.57	Vb 14	7.88
TEN-3	TRBV09	TRBV09-01	na	TRBJ02-01*01	420	CASSVSGQTSNEQFF	AGCTCTCTGGAGCTGGGGGACTCAGCTTTGTATTTCTGTGCCAGCAGTGTCAGCGGACAGACTAGCAATGAGCAGTTCTTCGGGCCA	1.95	Vb 1	3.89
	TRBV28	TRBV28-01*01	TRBD02-01	TRBJ01-01*01	243	CASRDSGGTEAFF	ATTCTGGAGTCCGCCAGCACCAACCAGACATCTATGTACCTCTGTGCCAGCCGGGACTCCGGAGGAACTGAAGCTTTCTTTGGACAA	1.12	Vb 3	4
	TRBV07	TRBV07-02*01	TRBD02-01*02	TRBJ02-01*01	233	CASSWVGGSNNNEQFF	CGCACACAGCAGGAGGACTCGGCCGTGTATCTCTGTGCCAGCAGCTGGGTGGGCGGGAGTAACAACAATGAGCAGTTCTTCGGGCCA	1.08	na	na
	TRBV19	TRBV19-01	TRBD02-01*02	TRBJ02-05*01	1374	CASSIVGGGETQYF	GTGACATCGGCCCAAAAGAACCCGACAGCTTTCTATCTCTGTGCCAGTAGTATAGTTGGAGGAGGGGAGACCCAGTACTTCGGGCCA	8.11	Vb 17	10.5
	TRBV27	TRBV27-01*01	TRBD01-01*01	TRBJ01-05*01	845	CASSLGARTAGPDGYQHF	AGCCCCAACCAGACCTCTCTGTACTTCTGTGCCAGCAGTTTAGGAGCCAGGACGGCAGGGCCTGACGGGTACCAGCATTTTGGTGAT	4.99	Vb 14	3.17
TEN-4	TRBV07	TRBV07-03*01	TRBD01-01*01	TRBJ02-01*01	265	CASSLLGQGDYNEQFF	CGCACAGAGCGGGGGGACTCAGCCGTGTATCTCTGTGCCAGCAGCTTGCTGGGACAGGGCGACTACAATGAGCAGTTCTTCGGGCCA	1.56	na	na
	TRBV07	TRBV07-02*01	TRBD01-01*01	TRBJ02-07*01	176	CASSFWHPYEQYF	ACGATCCAGCGCACACAGCAGGAGGACTCGGCCGTGTATCTCTGTGCCAGCAGCTTCTGGCACCCCTACGAGCAGTACTTCGGGCCG	1.04	na	na
	TRBV06	TRBV06-05*01	TRBD02-01*01	TRBJ02-01*01	123	CASSLAGPYNEQFF	CTGCTGTCGGCTGCTCCCTCCCAGACATCTGTGTACTTCTGTGCCAGCAGTTTAGCGGGGCCCTACAATGAGCAGTTCTTCGGGCCA	0.73	Vb 13.1	0.27
	TRBV13	TRBV13-01*01	TRBD01-01*01	TRBJ01-02*01	1951	CASSLSTGGWLDGYTF	TCCTTGGAGCTGGGGGACTCAGCCCTGTACTTCTGTGCCAGCAGCTTAAGTACAGGGGGCTGGTTAGATGGCTACACCTTCGGTTCG	2.51	Vb 23	3.6
	TRBV15	TRBV15-01*01	TRBD02-01*01	TRBJ02-05*01	1776	CATSRDVAGGGTQYF	CGCTCACCAGGCCTGGGGGACGCAGCCATGTACCTGTGTGCCACCAGCAGAGATGTAGCGGGGGGCGGGACCCAGTACTTCGGGCCA	2.28	na	na
TEN-5	TRBV28	TRBV28-01*01	TRBD01-01*01	TRBJ02-02*01	1701	CASSPPGDRNTGELFF	TCCGCCAGCACCAACCAGACATCTATGTACCTCTGTGCCAGCAGCCCGCCGGGGGACAGGAACACCGGGGAGCTGTTTTTTGGAGAA	2.19	Vb 3	5.43
	TRBV06	TRBV06-05*01	TRBD02-01*01	TRBJ02-01*01	916	CASSYLAGASYNEQFF	TCGGCTGCTCCCTCCCAGACATCTGTGTACTTCTGTGCCAGCAGTTACTTAGCGGGGGCTTCCTACAATGAGCAGTTCTTCGGGCCA	1.18	Vb 13.1	2.34
	TRBV27	TRBV27-01*01	na	TRBJ02-02*01	779	CASSFSPEGTGELFF	GAGTCGCCCAGCCCCAACCAGACCTCTCTGTACTTCTGTGCCAGCAGTTTCTCCCCAGAGGGGACCGGGGAGCTGTTTTTTGGAGAA	1.00	Vb 14	2.26
	TRBV07	TRBV07-02*01	na	TRBJ01-02*01	1748	CASSLRENYGYTF	ACGATCCAGCGCACACAGCAGGAGGACTCGGCCGTGTATCTCTGTGCCAGCAGCCTCAGGGAGAACTATGGCTACACCTTCGGTTCG	4.83	na	na
	TRBV18	TRBV18-01*01	TRBD01-01*01	TRBJ01-02*01	905	CASSPGQGRSYGYTF	CAGCAGGTAGTGCGAGGAGATTCGGCAGCTTATTTCTGTGCCAGCTCACCCGGACAGGGAAGAAGCTATGGCTACACCTTCGGTTCG	2.50	Vb 18	3.07
TEN-6	TRBV19	TRBV19-01	TRBD01-01*01	TRBJ02-01*01	769	CASRMPGSNEQFF	ACTGTGACATCGGCCCAAAAGAACCCGACAGCTTTCTATCTCTGTGCCAGTAGGATGCCAGGGTCGAATGAGCAGTTCTTCGGGCCA	2.13	Vb 17	6.78
	TRBV30	TRBV30-01*01	TRBD01-01*01	TRBJ01-02*01	754	CAWSGGTGASYGYTF	AAGAAGCTCCTTCTCAGTGACTCTGGCTTCTATCTCTGTGCCTGGAGCGGGGGGACAGGGGCCTCCTATGGCTACACCTTCGGTTCG	2.08	Vb 20	1.63
	TRBV27	TRBV27-01*01	na	TRBJ02-02*01	398	CASRRPGPNTGELFF	GAGTCGCCCAGCCCCAACCAGACCTCTCTGTACTTCTGTGCCAGCAGAAGGCCGGGACCGAACACCGGGGAGCTGTTTTTTGGAGAA	1.10	Vb 14	0.74
	TRBV06	TRBV06	TRBD02-01*02	TRBJ02-01*01	5623	CASSPTSGSSNEQFF	GAGTCGGCTGCTCCCTCCCAAACATCTGTGTACTTCTGTGCCAGCAGCCCGACTAGCGGGAGTAGTAATGAGCAGTTCTTCGGGCCA	11.20	Vb 13.1; Vb 13.2; Vb 13.6	na
	TRBV02	TRBV02-01*01	TRBD02-01*01	TRBJ02-07*01	4141	CASSEVVAGASSYEQYF	ACAAAGCTGGAGGACTCAGCCATGTACTTCTGTGCCAGCAGTGAAGTGGTAGCGGGGGCTAGCTCCTACGAGCAGTACTTCGGGCCG	8.25	Vb 22	4.83
TEN-7	TRBV20	TRBV20	na	TRBJ02-05*01	1525	CSAKQLNQETQYF	ACAGTGACCAGTGCCCATCCTGAAGACAGCAGCTTCTACATCTGCAGTGCTAAGCAGCTTAACCAAGAGACCCAGTACTTCGGGCCA	3.04	na	na
	TRBV06	TRBV06-01*01	TRBD01-01*01	TRBJ02-02*01	997	CASSELLWDRISGKELFF	GCTCCCTCCCAGACATCTGTGTACTTCTGTGCCAGCAGTGAACTTTTATGGGACAGAATATCCGGCAAGGAGCTGTTTTTTGGAGAA	1.99	na	na
	TRBV05	TRBV05-05*01	TRBD01-01*01	TRBJ01-01*01	646	CASSLNRDRSNTEAFF	GCCTTGTTGCTGGGGGACTCGGCCCTGTATCTCTGTGCCAGCAGCTTGAACAGGGACCGATCGAACACTGAAGCTTTCTTTGGACAA	1.29	Vb 5.3	2.74
	TRBV11	TRBV11-03*01	TRBD01-01*01	TRBJ01-05*01	82	CASSNPTGQGHSNQPQHF	GAGCTTGGGGACTCGGCCGTGTATCTCTGTGCCAGCAGCAACCCGACGGGACAGGGGCATAGCAATCAGCCCCAGCATTTTGGTGAT	0.55	na	na
	TRBV27	TRBV27-01*01	TRBD01-01*01	TRBJ02-03*01	47	CASFTSSYEQYF	CTGATCCTGGAGTCGCCCAGCCCCAACCAGACCTCTCTGTACTTCTGTGCCAGCAGGACAGGGCCAGATACGCAGTATTTTGGCCCA	0.31	Vb 14	0.97
TEN-8	TRBV04	TRBV04-03*01	na	TRBJ02-02*01	27	CASRYRNGHEQYF	ACCCTGCAGCCAGAAGACTCGGCCCTGTATCTCTGCGCCAGCAGCCAAGAAGGAGGGGGGAACACCGGGGAGCTGTTTTTTGGAGAA	0.18	Vb 7.2	1.29
	TRBV27	TRBV27-01*01	TRBD02-01	TRBJ02-07*01	25	CASSQGAGSTNQPQHF	CTGATCCTGGAGTCGCCCAGCCCCAACCAGACCTCTCTGTACTTCTGTGCCAGCTTTACTAGCTCCTACGAGCAGTACTTCGGGCCG	0.17	Vb 14	0.97
	TRBV04	TRBV04-03*01	TRBD01-01*01	TRBJ01-05*01	20	CASRTGPDTQYF	ACCCTGCAGCCAGAAGACTCGGCCCTGTATCTCTGCGCCAGCAGCCAAGGTGCAGGATCGACGAATCAGCCCCAGCATTTTGGTGAT	0.13	Vb 7.2	1.29
	TRBV29	TRBV29-01*01	TRBD02-01*01	TRBJ01-02*01	23	CSVHSFGGASGYTF	GTGAGCAACATGAGCCCTGAAGACAGCAGCATATATCTCTGCAGCGTTCATTCCTTTGGGGGGGCAAGTGGCTACACCTTCGGTTCG	3.40	Vb 4	0.14
	TRBV21	TRBV21-01*01	TRBD01-01*01	TRBJ01-01*01	22	CASSKHPGLNTEAFF	CAGTCCACGGAGTCAGGGGACACAGCACTGTATCTCTGTGCCAGCAGCAAACACCCAGGGTTAAACACTGAAGCTTTCTTTGGACAA	3.25	na	na
TEN-9	TRBV06	TRBV06	TRBD01-01*01	TRBJ01-01*01	18	CASSYSDPRDMNTEAFF	GCTGCTCCCTCCCAAACATCTGTGTACTTCTGTGCCAGCAGTTACTCCGATCCGAGGGACATGAACACTGAAGCTTTCTTTGGACAA	2.66	na	na
	TRBV24	TRBV24	na	TRBJ02-01*01	17	CATSPDPPADEQFF	CTAGAGTCTGCCATCCCCAACCAGACAGCTCTTTACTTCTGTGCCACCAGCCCGGACCCCCCGGCTGATGAGCAGTTCTTCGGGCCA	2.51	na	na
	TRBV02	TRBV02-01*01	TRBD02-01*02	TRBJ01-05*01	15	CASSDVGRTSNQPQHF	TCCACAAAGCTGGAGGACTCAGCCATGTACTTCTGTGCCAGCAGTGATGTGGGGAGAACTAGCAATCAGCCCCAGCATTTTGGTGAT	2.22	Vb 22	2.49
	TRBV28	TRBV28-01*01	TRBD02-01*01	TRBJ01-04*01	87	CASSFGGPNEKLFF	CTGGAGTCCGCCAGCACCAACCAGACATCTATGTACCTCTGTGCCAGCAGTTTCGGGGGGCCTAATGAAAAACTGTTTTTTGGCAGT	2.16	Vb 3	0.17
	TRBV06	TRBV06	TRBD01-01*01	TRBJ02-02*01	81	CASSYGQVATGELFF	GAGTCGGCTGCTCCCTCCCAAACATCTGTGTACTTCTGTGCCAGCAGTTATGGACAGGTCGCCACCGGGGAGCTGTTTTTTGGAGAA	2.01	na	na
TEN-10	TRBV27	TRBV27-01*01	TRBD02-01*02	TRBJ01-01*01	32	CASNLGGVNTEAFF	CTGGAGTCGCCCAGCCCCAACCAGACCTCTCTGTACTTCTGTGCCAGCAACCTAGGAGGGGTGAACACTGAAGCTTTCTTTGGACAA	0.80	Vb 14	0.11
	TRBV04	TRBV04-03*01	TRBD01-01*01	TRBJ02-07*01	27	CASSQDLGPVEYEQYF	ACCCTGCAGCCAGAAGACTCGGCCCTGTATCTCTGCGCCAGCAGCCAAGATCTGGGGCCAGTAGAGTACGAGCAGTACTTCGGGCCG	0.67	Vb 7.2	0.34
	TRBV24	TRBV24	TRBD01-01*01	TRBJ02-07*01	20	CATKDLRGPASEQYF	GAGTCTGCCATCCCCAACCAGACAGCTCTTTACTTCTGTGCCACCAAGGATTTAAGGGGCCCTGCTTCAGAGCAGTACTTCGGGCCG	0.50	na	na
	TRBV28	TRBV28-01*01	TRBD02-01	TRBJ02-01*01	800	CASSLTTSGSYNEQFF	TCCGCCAGCACCAACCAGACATCTATGTACCTCTGTGCCAGCAGTTTAACGACTAGCGGATCCTACAATGAGCAGTTCTTCGGGCCA	5.18	Vb 3	14.9
	TRBV07	TRBV07-06*01	na	TRBJ02-03*01	258	CASSLWVSTDTQYF	ATCCAGCGCACAGAGCAGCGGGACTCGGCCATGTATCGCTGTGCCAGCAGCTTATGGGTGAGCACAGATACGCAGTATTTTGGCCCA	1.67	na	na
TEN-11	TRBV27	TRBV27-01*01	TRBD02-01	TRBJ02-07*01	100	CASSLVPGLNYEQYF	GAGTCGCCCAGCCCCAACCAGACCTCTCTGTACTTCTGTGCCAGCAGTTTGGTCCCGGGACTCAACTACGAGCAGTACTTCGGGCCG	0.65	Vb 14	0.021
	TRBV10	TRBV10-02*01	na	TRBJ02-05*01	79	CASSGVYETQYF	CTCACTCTGGAGTCAGCTACCCGCTCCCAGACATCTGTGTATTTCTGCGCCAGCAGTGGTGTTTACGAGACCCAGTACTTCGGGCCA	0.51	Vb 12	0.51
	TRBV19	TRBV19-01	TRBD02-01	TRBJ01-01*01	74	CASSPIPWTLNTEAFF	TCGGCCCAAAAGAACCCGACAGCTTTCTATCTCTGTGCCAGTAGTCCCATTCCCTGGACTTTGAACACTGAAGCTTTCTTTGGACAA	0.48	Vb 17	3.55
	TRBV27	TRBV27-01*01	na	TRBJ01-02*01	755	CASSYHGAADGYTF	CTGGAGTCGCCCAGCCCCAACCAGACCTCTCTGTACTTCTGTGCCAGCAGTTACCACGGGGCTGCGGATGGCTACACCTTCGGTTCG	0.86	Vb 14	nd
	TRBV03	TRBV03	TRBD01-01*01	TRBJ01-01*01	186	CASSPHDKNTEAFF	ATCAATTCCCTGGAGCTTGGTGACTCTGCTGTGTATTTCTGTGCCAGCAGCCCGCATGACAAGAACACTGAAGCTTTCTTTGGACAA	0.21	na	nd
TEN-12	TRBV14	TRBV14-01*01	TRBD02-01*01	TRBJ02-01*01	166	CASSQLAGAYNEQFF	CAGCCTGCAGAACTGGAGGATTCTGGAGTTTATTTCTGTGCCAGCAGCCAACTAGCGGGGGCCTACAATGAGCAGTTCTTCGGGCCA	0.19	Vb 16	nd
	TRBV05	TRBV05-04*01	TRBD02-01*01	TRBJ02-07*01	154	CASSLGRGGSYEQYF	AACGCCTTGGAGCTGGACGACTCGGCCCTGTATCTCTGTGCCAGCAGCTTGGGTCGGGGGGGCTCCTACGAGCAGTACTTCGGGCCG	0.18	na	nd
	TRBV07	TRBV07-08*01	TRBD01-01*01	TRBJ02-07*01	151	CASSLGQAYEQYF	AAGATCCAGCGCACACAGCAGGAGGACTCCGCCGTGTATCTCTGTGCCAGCAGCTTAGGCCAGGCCTACGAGCAGTACTTCGGGCCG	0.17	na	nd
	TRBV28	TRBV28-01*01	TRBD02-01	TRBJ02-01*01	646	CASSLTTTRNEQFF	CTGGAGTCCGCCAGCACCAACCAGACATCTATGTACCTCTGTGCCAGCAGTTTGACTACAACACGGAATGAGCAGTTCTTCGGGCCA	0.81	Vb 3	8.17
	TRBV11	TRBV11-03*01	TRBD02-01	TRBJ01-01*01	349	CASSVSLATEAFF	AAGATCCAGCCTGCAGAGCTTGGGGACTCGGCCGTGTATCTCTGTGCCAGCAGCGTAAGTTTAGCAACGGAAGCTTTCTTTGGACAA	0.44	na	na
TEN-13	TRBV06	TRBV06-01*01	TRBD02-01*01	TRBJ01-05*01	201	CASSWGSGGNQPQHF	GAGTCGGCTGCTCCCTCCCAGACATCTGTGTACTTCTGTGCCAGCAGTTGGGGGTCGGGGGGCAATCAGCCCCAGCATTTTGGTGAT	0.25	na	na
	TRBV05	TRBV05-01*01	na	TRBJ02-02*01	172	CASSFDGGDGELFF	GTGAGCACCTTGGAGCTGGGGGACTCGGCCCTTTATCTTTGCGCCAGCAGCTTTGATGGGGGAGACGGGGAGCTGTTTTTTGGAGAA	0.21	Vb 5.1	5.03
	TRBV06	TRBV06-05*01	TRBD02-01*02	TRBJ02-01*01	146	CASSYTGGRAYNEQFF	TCGGCTGCTCCCTCCCAGACATCTGTGTACTTCTGTGCCAGCAGTTACACGGGCGGGAGGGCCTACAATGAGCAGTTCTTCGGGCCA	0.18	Vb 13.1	4.02
	TRBV06	TRBV06-05*01	TRBD02-01*01	TRBJ02-05*01	97	CASSFVGGGGRETQYF	TCGGCTGCTCCCTCCCAGACATCTGTGTACTTCTGTGCCAGCAGTTTCGTGGGCGGGGGGGGCCGAGAGACCCAGTACTTCGGGCCA	0.30	Vb 13.1	na
	TRBV07	TRBV07-03*01	TRBD01-01*01	TRBJ01-01*01	87	CASSWTENTEAFF	AAGATCCAGCGCACAGAGCGGGGGGACTCAGCCGTGTATCTCTGTGCCAGCAGCTGGACAGAGAACACTGAAGCTTTCTTTGGACAA	0.27	na	na
TEN-14	TRBV07	TRBV07-08*01	unknown	TRBJ01-05*01	79	CASPLWQPQHF	ACTCTGAAGATCCAGCGCACACAGCAGGAGGACTCCGCCGTGTATCTCTGTGCCAGCCCCTTGTGGCAGCCCCAGCATTTTGGTGAT	0.24	na	na
	TRBV05	TRBV05-06*01	unknown	TRBJ02-01*01	50	CASSWGINEQFF	CTGAATGTGAACGCCTTGTTGCTGGGGGACTCGGCCCTCTATCTCTGTGCCAGCAGCTGGGGGATCAATGAGCAGTTCTTCGGGCCA	0.15	Vb 5.2	na
	TRBV29	TRBV29-01*01	TRBD01-01*01	TRBJ02-02*01	47	CSVGATNTGELFF	ACTGTGAGCAACATGAGCCCTGAAGACAGCAGCATATATCTCTGCAGCGTTGGGGCCACGAACACCGGGGAGCTGTTTTTTGGAGAA	0.14	Vb 4	na
	TRBV06	TRBV06-05*01	unknown	TRBJ02-03*01	1504	CASSFSGSDTQYF	AGGCTGCTGTCGGCTGCTCCCTCCCAGACATCTGTGTACTTCTGTGCCAGCAGTTTCTCGGGTTCGGATACGCAGTATTTTGGCCCA	6.17	Vb 13.1	48
	TRBV21	TRBV21-01*01	unknown	TRBJ01-01*01	877	CASSWGNTEAFF	TTGGAGATCCAGTCCACGGAGTCAGGGGACACAGCACTGTATTTCTGTGCCAGCTCCTGGGGGAACACTGAAGCTTTCTTTGGACAA	3.60	na	na
TEN-15	TRBV12	TRBV12	unknown	TRBJ01-05*01	721	CASSVNYSNQPQHF	ATCCAGCCCTCAGAACCCAGGGACTCAGCTGTGTACTTCTGTGCCAGCAGTGTGAATTATAGCAATCAGCCCCAGCATTTTGGTGAT	2.96	Vb 8	13.8
	TRBV07	TRBV07-09	TRBD02-01*01	TRBJ02-07*01	250	CASTPGDRATYEQYF	CAGCGCACAGAGCAGGGGGACTCGGCCATGTATCTCTGTGCCAGCACCCCGGGGGATAGGGCAACCTACGAGCAGTACTTCGGGCCG	1.02	na	na
	TRBV12	TRBV12	unknown	TRBJ01-04*01	234	CASSPNYSNEKLFF	ATCCAGCCCTCAGAACCCAGGGACTCAGCTGTGTACTTCTGTGCCAGCAGTCCGAATTATTCCAATGAAAAACTGTTTTTTGGCAGT	0.96	Vb 8	13.8
	TRBV19	TRBV19-01	TRBD01-01*01	TRBJ02-02*01	6434	CASSKVQTPGELFF	GTGACATCGGCCCAAAAGAACCCGACAGCTTTCTATCTCTGTGCCAGTAGTAAGGTACAGACCCCGGGGGAGCTGTTTTTTGGAGAA	13.50	Vb 17	na
	TRBV12	TRBV12	TRBD01-01*01	TRBJ01-01*01	6380	CASSPNDGQGDEAFF	CAGCCCTCAGAACCCAGGGACTCAGCTGTGTACTTCTGTGCCAGCAGTCCGAATGACGGACAGGGGGATGAAGCTTTCTTTGGACAA	13.38	Vb 8	na
TEN-16	TRBV10	TRBV10-01	TRBD02-01*02	TRBJ02-03*01	1119	CASSDSGSPIPEYF	CTGGAGTCTGCTGCCTCCTCCCAGACATCTGTATATTTCTGCGCCAGCAGTGATAGCGGGAGCCCAATACCGGAGTATTTTGGCCCA	2.35	Vb 12	na
	TRBV02	TRBV02-01*01	TRBD01-01*01	TRBJ02-07*01	682	CASSRTGGIYEQYF	ATCCGGTCCACAAAGCTGGAGGACTCAGCCATGTACTTCTGTGCCAGCAGTCGGACAGGGGGGATTTACGAGCAGTACTTCGGGCCG	1.43	Vb 22	na
	TRBV07	TRBV07-06*01	TRBD02-01*01	TRBJ01-04*01	485	CASSLAPGSTNEKLFF	CGCACAGAGCAGCGGGACTCGGCCATGTATCGCTGTGCCAGCAGCTTAGCGCCGGGGTCAACTAATGAAAAACTGTTTTTTGGCAGT	1.02	na	na

Table S8: Comparison of the productive frequency of the 5 most common TCR-β clonotypes found in PBMC TEN samples, and the frequency of respective TCR-Vβ chains detected by FACS.

Genomic DNA extracted from PBMC TEN samples was used for survey level deep sequencing of the TCRβ-chain, using ImmunoSEQ^{TM} platform. The TRBV repertoire and TCR-Vβ FACS analysis is described in the footnotes of Table S5.

Patient	V family	TRBV	TRBD	TRBJ	Templates	Amino-acid	CDR3 rearrangement	Productive frequency	Respective anti-Vβ mAb	% Vβ+ cells among CD3+CD8+ T cells as determined by FACs analysis
	TRBV05	TRBV05-04*01	TRBD01-01*01	TRBJ01-02*01	621	CASSLGWADYGYTF	GTGAACGCCTTGGAGCTGGACGACTCGGCCCTGTATCTCTGTGCCAGCAGCTTGGGGTGGGCTGACTATGGCTACACCTTCGGTTCG	3.33	unknown	na
	TRBV05	TRBV05-06*01	TRBD01-01*01	TRBJ01-04*01	391	CASSDRYRAEKLFF	GTGAACGCCTTGTTGCTGGGGGACTCGGCCCTCTATCTCTGTGCCAGCAGCGACAGATACAGGGCCGAAAAACTGTTTTTTGGCAGT	2.10	Vb 5.2	0.81
MPE-3	TRBV04	TRBV04-03*01	TRBD02-01*02	TRBJ02-01*01	386	CASSPSGGGRLSNEQFF	CTGCAGCCAGAAGACTCGGCCCTGTATCTCTGCGCCAGCAGCCCATCTGGGGGAGGGCGACTCTCCAATGAGCAGTTCTTCGGGCCA	2.07	Vb 7.2	0
	TRBV15	TRBV15-01*01	TRBD02-01*02	TRBJ02-05*01	377	CATSRTGRWETQYF	ATCCGCTCACCAGGCCTGGGGGACGCAGCCATGTACCTGTGTGCCACCAGCCGAACGGGGAGGTGGGAGACCCAGTACTTCGGGCCA	2.02	unknown	na
	TRBV11	TRBV11-02*02	TRBD01-01*01	TRBJ02-07*01	354	CASSSRDRGYEQYF	ATCCAGCCTGCAAAGCTTGAGGACTCGGCCGTGTATCTCTGTGCCAGCAGCTCCCGGGACAGGGGCTACGAGCAGTACTTCGGGCCG	1.90	Vb 21.3	3.94
	TRBV07	TRBV07-09	TRBD01-01*01	TRBJ01-02*01	101	CASSLAGTGYGYTF	ATCCAGCGCACAGAGCAGGGGGACTCGGCCATGTATCTCTGTGCCAGCAGCTTAGCGGGGACAGGATATGGCTACACCTTCGGTTCG	2.10	unknown	na
	TRBV06	TRBV06	TRBD01-01*01	TRBJ01-02*01	59	CASSPGTGSLGYGYTF	TCGGCTGCTCCCTCCCAGACATCTGTGTACTTCTGTGCCAGCAGTCCCGGGACAGGCTCTCTTGGCTATGGCTACACCTTCGGTTCG	1.23	unknown	na
MPE-4	TRBV07	TRBV07-09	unknown	TRBJ01-03*01	51	CASSSLTVLLGGNTIYF	ACAGAGCAGGGGGACTCGGCCATGTATCTCTGTGCCAGCAGCTCACTTACAGTACTCCTCGGGGGAAACACCATATATTTTGGAGAG	1.06	unknown	na
	TRBV05	TRBV05-04*01	TRBD02-01	TRBJ02-01*01	19	CASSLGTLEQFF	CTGAATGTGAACGCCTTGGAGCTGGACGACTCGGCCCTGTATCTCTGTGCCAGCAGCTTGGGGACTTTGGAGCAGTTCTTCGGGCCA	0.39	unknown	na
	TRBV07	TRBV07-02*01	TRBD02-01*01	TRBJ01-02*01	13	CASSPTGGGNGYTF	ATCCAGCGCACACAGCAGGAGGACTCGGCCGTGTATCTCTGTGCCAGCAGCCCCACGGGGGGCGGGAATGGCTACACCTTCGGTTCG	0.27	unknown	na
	TRBV04	TRBV04-01*01	unknown	TRBJ02-01*01	283	CASSLLDGEQFF	CTTCACCTACACGCCCTGCAGCCAGAAGACTCAGCCCTGTATCTCTGCGCCAGCAGCCTACTGGACGGAGAGCAGTTCTTCGGGCCA	3.31	Vb 7.1	2.81
	TRBV10	TRBV10-03*01	TRBD01-01*01	TRBJ02-07*01	281	CAISEGGAGTGSEQYF	TCCGCTACCAGCTCCCAGACATCTGTGTACTTCTGTGCCATCAGTGAGGGGGGCGCCGGGACAGGCTCCGAGCAGTACTTCGGGCCG	3.28	Vb 12	7.74
MPE-5	TRBV20	TRBV20	TRBD02-01*02	TRBJ02-01*01	155	CSARDLEVGWNEQFF	ACCAGTGCCCATCCTGAAGACAGCAGCTTCTACATCTGCAGTGCTAGAGATCTCGAGGTTGGGTGGAATGAGCAGTTCTTCGGGCCA	1.81	unknown	na
	TRBV07	TRBV07-09	unknown	TRBJ01-01*01	127	CASVPLSGNTEAFF	ATCCAGCGCACAGAGCAGGGGGACTCGGCCATGTATCTCTGTGCCAGCGTACCCTTGTCGGGCAACACTGAAGCTTTCTTTGGACAA	1.48	unknown	na
	TRBV07	TRBV07-02*01	TRBD01-01*01	TRBJ02-07*01	68	CATRPGPTRGPSSYEQYF	CAGCAGGAGGACTCGGCCGTGTATCTCTGTGCCACCAGGCCCGGGCCGACGAGGGGGCCGAGCTCCTACGAGCAGTACTTCGGGCCG	0.79	unknown	na
	TRBV03	TRBV03	TRBD02-01	TRBJ02-01*01	447	CASRGSGSYNEQFF	ATCAATTCCCTGGAGCTTGGTGACTCTGCTGTGTATTTCTGTGCCAGCAGGGGGAGCGGCTCCTACAATGAGCAGTTCTTCGGGCCA	0.68	unknown	na
	TRBV28	TRBV28-01*01	TRBD01-01*01	TRBJ01-01*01	410	CASSSVFRTGATEAFF	TCCGCCAGCACCAACCAGACATCTATGTACCTCTGTGCCAGCAGTTCGGTGTTCAGGACAGGGGCCACTGAAGCTTTCTTTGGACAA	0.63	Vb 3	na
MPE-6	TRBV07	TRBV07-09	TRBD01-01*01	TRBJ01-04*01	75	CASSPASGTEGEKLFF	CGCACAGAGCAGGGGGACTCGGCCATGTATCTCTGTGCCAGCAGCCCCGCGTCCGGGACAGAGGGTGAAAAACTGTTTTTTGGCAGT	0.11	unknown	na
	TRBV28	TRBV28-01*01	TRBD02-01*02	TRBJ02-07*01	74	CASSLGGVAYEQYF	CTGGAGTCCGCCAGCACCAACCAGACATCTATGTACCTCTGTGCCAGCAGTTTAGGAGGGGTGGCCTACGAGCAGTACTTCGGGCCG	0.11	Vb 3	na
	TRBV05	TRBV05-01*01	TRBD01-01*01	TRBJ01-02*01	64	CASSRTGYYGYTF	AATGTGAGCACCTTGGAGCTGGGGGACTCGGCCCTTTATCTTTGCGCCAGCAGCCGGACAGGCTATTATGGCTACACCTTCGGTTCG	0.10	Vb 5.1	na
	TRBV30	TRBV30-01*01	unknown	TRBJ01-01*01	249	CAWSLENTEAFF	CTGAGTTCTAAGAAGCTCCTTCTCAGTGACTCTGGCTTCTATCTCTGTGCCTGGAGTTTGGAGAACACTGAAGCTTTCTTTGGACAA	0.98	Vb 20	6.42
	TRBV03	TRBV03	unknown	TRBJ01-06*01	140	CASSSGNGNSPLHF	ATCAATTCCCTGGAGCTTGGTGACTCTGCTGTGTATTTCTGTGCCAGCAGCTCCGGCAATGGGAATTCACCCCTCCACTTTGGGAAC	0.55	unknown	na
MPE-7	TRBV09	TRBV09-01	TRBD01-01*01	TRBJ02-02*01	125	CASSRRRRAGNPNTGELFF	CTGGGGGACTCAGCTTTGTATTTCTGTGCCAGCAGCCGCCGAAGACGTGCAGGGAATCCGAACACCGGGGAGCTGTTTTTTGGAGAA	0.49	Vb 1	2.72
	TRBV12	TRBV12	TRBD01-01*01	TRBJ02-07*01	121	CASSFGGTNYEQYF	ATCCAGCCCTCAGAACCCAGGGACTCAGCTGTGTACTTCTGTGCCAGCAGTTTTGGTGGGACAAATTACGAGCAGTACTTCGGGCCG	0.48	unknown	na
	TRBV15	TRBV15-01*01	TRBD01-01*01	TRBJ01-01*01	99	CATSRDRQEAFF	CTTGACATCCGCTCACCAGGCCTGGGGGACGCAGCCATGTACCTGTGTGCCACCAGCAGAGATCGACAGGAAGCTTTCTTTGGACAA	0.39	unknown	na

Table S9: Comparison of the productive frequency of the 5 most common TCR-β clonotypes found in PBMC MPE samples, and the frequency of respective TCR-Vβ chains detected by FACS.

Genomic DNA extracted from PBMC MPE samples were used for survey level deep sequencing of the TCRβ-chain, using ImmunoSEQ™ platform. The TRBV repertoire and TCR-Vβ FACS analysis is described in the footnotes of Table S5.

Patient	Sorted Vβ	V family	TRAV	TRAJ	Templates	Amino-acid	CDR3 rearrangement	Productive Frequency
TEN-3	Vβ21.3	TRAV25	TRAV25-01*01	TRAJ43-01*01	13167	CANNDMRF	CAGCTCCCTGCACATCACAGCCACCCAGACTACAGATGTAGGAACCTACTTCTGTGCCAACAATGACATGCGCTTTGGAGCA	87.6
		TRAV25	TRAV25-01*01	TRAJ43-01*01	1309	CANNDMRF	CAGTTCCCTGCACATCACAGCCACCCAGACTACAGATGTAGGAACCTACTTCTGTGCCAACAATGACATGCGCTTTGGAGCA	8.5
		TRAV19	TRAV19-01*01	TRAJ17-01*01	55	CALSEAHPRGKAAGNKLTF	GGACTCAGCAGTATACTTCTGTGCTCTGAGTGAGGCGCATCCCCGTGGTAAAGCTGCAGGCAACAAGCTAACTTTTGGAGGA	0.4
		TRAV39	TRAV39-01*01	TRAJ27-01*01	32	CAVVRPNAGKSTF	CACAGCTGCCGTGCATGACCTCTCTGCCACCTACTTCTGTGCCGTGGTCAGACCCAATGCAGGCAAATCAACCTTTGGGGAT	0.1
		TRAV20	TRAV20-01	TRAJ42-01*01	10	CAVQVLYGGSQGNLIF	TAAACCTGAAGACTCAGCCACTTATCTCTGTGCTGTGCAGGTCTTATATGGAGGAAGCCAAGGAAATCTCATCTTTGGAAAA	0.07
TEN-7	Vβ13.2	TRAV13	TRAV13-01	TRAJ39-01*01	284	CADNAGNMLTF	GCACATCACAGAGACCCAACCTGAAGACTCGGCTGTCTACTTCTGTGCCGATAATGCAGGCAACATGCTCACCTTTGGAGGG	86.95
		TRAV14	TRAV14-01	TRAJ53-01*01	24	CAMREGQSGGSNYKLTF	ACTGGGGGACTCAGCAATGTATTTCTGTGCAATGAGAGAGGGCCAGAGTGGAGGTAGCAACTATAAACTGACATTTGGAAAA	7.52
		TRAV29	TRAV29-01	TRAJ52-01*01	4	CAANSGYGKLTF	CATTGTGCCCTCCCAGCCTGGAGACTCTGCAGTGTACTTCTGTGCAGCAAATAGCGGCTATGGAAAGCTGACATTTGGACAA	1.62
		TRAV39	TRAV39-01*01	TRAJ45-01*01	2	CAVPPGGGADGLTF	AGCTGCCGTGCATGACCTCTCTGCCACCTACTTCTGTGCCGTCCCCCCAGGAGGAGGTGCTGACGGACTCACCTTTGGCAAA	0.93
		TRAV12	TRAV12-01	TRAJ50-01*01	2	CVVLYDKVIF	CCTGCTCATCAGAGACTCCAAGCTCAGTGATTCAGCCACCTACCTCTGTGTGGTTCTCTACGACAAGGTGATATTTGGGCCA	0.66
TEN-10	Vβ3	TRAV27	TRAV27-01*01	TRAJ26-01*01	170295	CAGDDNYGQNFVF	CACTGCAGCCCAGCCTGGTGATACAGGCCTCTACCTCTGTGCAGGGGACGATAACTATGGTCAGAATTTTGTCTTTGGTCCC	89.84
		TRAV21	TRAV21-01	TRAJ20-01*01	817	CAVRPNDYKLSF	CATTGCAGCTTCTCAGCCTGGTGACTCAGCCACCTACCTCTGTGCTGTGAGGCCAAACGACTACAAGCTCAGCTTTGGAGCC	0.43
		TRDV02	TRDV02-01	TRDJ01-01*01	752	CACDPVSWETGDQTDKLIF	AGATGAAGGGTCTTACTACTGTGCCTGTGACCCTGTTTCCTGGGAGACTGGGGATCAAACCGATAAACTCATCTTTGGAAAA	0.39
		TRAV21	TRAV21-01	TRAJ47-01*01	544	CAVKELEYGNKLVF	AGCTTCTCAGCCTGGTGACTCAGCCACCTACCTCTGTGCTGTGAAGGAATTGGAATATGGAAACAAACTGGTCTTTGGCGCA	0.29
		TRAV21	TRAV21-01	TRAJ49-01*01	436	CAVRPTFTGNQFYF	AGCTTCTCAGCCTGGTGACTCAGCCACCTACCTCTGTGCTGTGAGGCCAACTTTCACCGGTAACCAGTTCTATTTTGGGACA	0.22
TEN-15	Vβ14	TRAV39	TRAV39-01*01	TRAJ40-01*01	2732	CAVDIVGYKYIF	CATCACAGCTGCCGTGCATGACCTCTCTGCCACCTACTTCTGTGCCGTGGACATTGTTGGCTACAAATACATCTTTGGAACA	96.08
		TRAV24	TRAV24-01*01	TRAJ22-01*01	32	CASLSGSARQLTF	CAAAGGATCCCAGCCTGAAGACTCAGCCACATACCTCTGTGCCTCTCTTTCTGGTTCTGCAAGGCAACTGACCTTTGGATCT	1.23
		TRAV12	TRAV12-01	TRAJ41-01*01	11	CVVRDSGYALNF	CATCAGAGACTCCAAGCTCAGTGATTCAGCCACCTACCTCTGTGTGGTGAGGGATTCCGGGTATGCACTCAACTTCGGCAAA	0.42
		TRAV26	TRAV26-01	TRAJ37-01	9	CIGGFRVCTQL	GATCCTGCCCCACGCTACGCTGAGAGACACTGCTGTGTACTATTGCATCGGCGGATTCCGGGTATGCACTCAACTTCGGCAA	0.38
		TRAV19	TRAV19-01*01	TRAJ18-01*01	6	CAPDRGSTLGRLYF	AGCCTCACAAGTCGTGGACTCAGCAGTATACTTCTGTGCTCCCGACAGAGGCTCAACCCTGGGGAGGCTATACTTTGGAAGA	0.19

Table S10: Identification of the main TCR-α chains expressed by dominant TCR-Vβ+ cells.

Dominant CD8+TCR-Vβ+ cells were FACS sorted from the blister or PBMC samples of patients TEN-3, -7, -10 and -15. Genomic DNA was extracted and used for survey level deep sequencing of the TCRα-chain, using ImmunoSEQ™ platform. Information for Vα family, TRAV, TRAJ, template number, amino acid and CDR3 rearrangement and productive frequency are provided, as described in Table S5.

Patient	Sorted Vβ	V family	TRAV	TRAJ	Templates	CDR3 rearrangement	Unproductive TCRα chain Frequency
TEN-3	Vβ21.3	TRAV23	TRAV23-01	TRAJ37-01	14520	AAGCAGTTCTCATTGCATATCATGGATTCCCAGCCTGGAGACTCAGCCACCTAGGAACACAGGCAAACTAATCTTTGGGCAA	47
		TRAV23	TRAV23-01	TRAJ37-01	164	AAGCACCTCTCATTGCATATCATGGATTCCCAGCCTGGAGACTCAGCCACCTAGGAACACAGGCAAACTAATCTTTGGGCAA	0.6
TEN-7	Vβ13.2	TRAV35	TRAV35-01	TRAJ27-01*01	3	TTCCTGAATATCTCAGCATCCATACCTAGTGATGTAGGCATCTACTTCTGTGCTGGGCAGGGCCAAATCAACCTTTGGGGAT	1.2
		TRAV01	TRAV01-01	TRAJ30-01*01	3	TTCTACAGGAGCTCCAGATGAAAGACTCTGCCTCTTACTTCTGCGCTGTGAGAGGGGGCCGGGCAAGATCATCTTTGGAAAA	1.1
TEN-10	Vβ3	TRAV36	TRAV36-01	TRAJ40-01*01	94386	ACAGCCACCCAGACCGGAGACTCGGCCGTCTACCTCTGTGCTGTGGAGTGCCTCAGGAACCTACAAATACATCTTTGGAACA	31.5
		TRAV25	TRAV25-01*01	TRAJ30-01*01	672	ACAGCTCCCTGCACATCACAGCCACCCAGACTACAGATGTAGGAACCTCCTGAACAGAGATGACAAGATCATCTTTGGAAAA	0.2
TEN-15	Vβ14	TRAV27	TRAV27-01*01	TRAJ26-01*01	170295	CACTGCAGCCCAGCCTGGTGATACAGGCCTCTACCTCTGTGCAGGGGACGATAACTATGGTCAGAATTTTGTCTTTGGTCCC	50.2
		TRDV02	TRDV02-01	TRDJ01-01*01	752	AGATGAAGGGTCTTACTACTGTGCCTGTGACCCTGTTTCCTGGGAGACTGGGGATCAAACCGATAAACTCATCTTTGGAAAA	0.4

Table S11. Main unproductive TCR-α chains found in dominant TCR-Vβ+ cells.

Dominant CD8+TCR-Vβ+ cells from the blister or PBMC samples of patients TEN-3, -7, -10 and -15 were FACS sorted and analysed as described in Table S10. Information for nature and frequency of unproductive TCR-α chains are provided.

	Dominant clonotypes obtained through Vβ sorting						TCR transfectant ID
	Target clone	Chains	V family	TCR(A/B)V	TCR(A/B)J	Rearrangement	
**TEN-3**	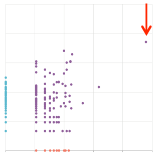	β	TRBV11	TRBV11-02*02	TRBJ02-01*01	CAGCCTGCAAAGCTTGAGGACTCGGCCGTGTATCTCTGTGCCAGCAGCCCCTTCCGTGACTCCTACAATGAGCAGTTCTTCGGGCCA	C1
		α	TRAV25	TRAV25-01*01	TRAJ43-01*01	CAGCTCCCTGCACATCACAGCCACCCAGACTACAGATGTAGGAACCTACTTCTGTGCCAACAATGACATGCGCTTTGGAGCA	
**TEN-7**	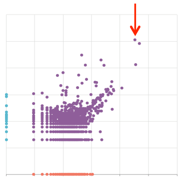	β	TRBV06	TRBV06	TRBJ02-01*01	GAGTCGGCTGCTCCCTCCCAAACATCTGTGTACTTCTGTGCCAGCAGCCCGACTAGCGGGAGTAGTAATGAGCAGTTCTTCGGGCCA	C2
		α	TRAV13	TRAV13-01	TRAJ39-01*01	GCACATCACAGAGACCCAACCTGAAGACTCGGCTGTCTACTTCTGTGCCGATAATGCAGGCAACATGCTCACCTTTGGAGGG	
**TEN-10**	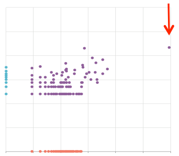	β	TRBV28	TRBV28-01*01	TRBJ01-04*01	CTGGAGTCCGCCAGCACCAACCAGACATCTATGTACCTCTGTGCCAGCAGTTTCGGGGGGCCTAATGAAAAACTGTTTTTTGGCAGT	C3
		α	TRAV27	TRAV27-01*01	TRAJ26-01*01	AGCCTCACAAGTCGTGGACTCAGCAGTATACTTCTGTGCTCTGAGTGAGGCGTGGGGAAACACACCTCTTGTCTTTGGAAAG	
**TEN-15**	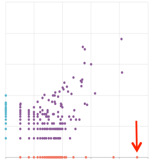	β	TRBV27	TRBV27-01*01	TRBJ02-01*01	CTGGAGTCGCCCAGCCCCAACCAGACCTCTCTGTACTTCTGTGCCAGCAGTCTAGCGGGGGGGCTGGGTGAGCAGTTCTTCGGGCCA	C4
		α	TRAV39	TRAV39-01*01	TRAJ40-01*01	CATCACAGCTGCCGTGCATGACCTCTCTGCCACCTACTTCTGTGCCGTGGACATTGTTGGCTACAAATACATCTTTGGAACA	

Table S12. Paired TCRα and TCRβ sequences used to generate Skw3 transductants.

Table shows minimal information (Vβ/Vα family, TRB/AV, TRB/AD, TRB/AJ, CDR3 rearrangement) for the TCRα and TCRβ chain rearrangement CDR3 sequences that were transduced in Skw3 cells to test drug-specificity of dominant TCR clonotypes from patients TEN-3, -7, -10 and -15. The targeted top clone in paired blister/PBMC heat map scatters is shown, as well as respective transfectant ID.

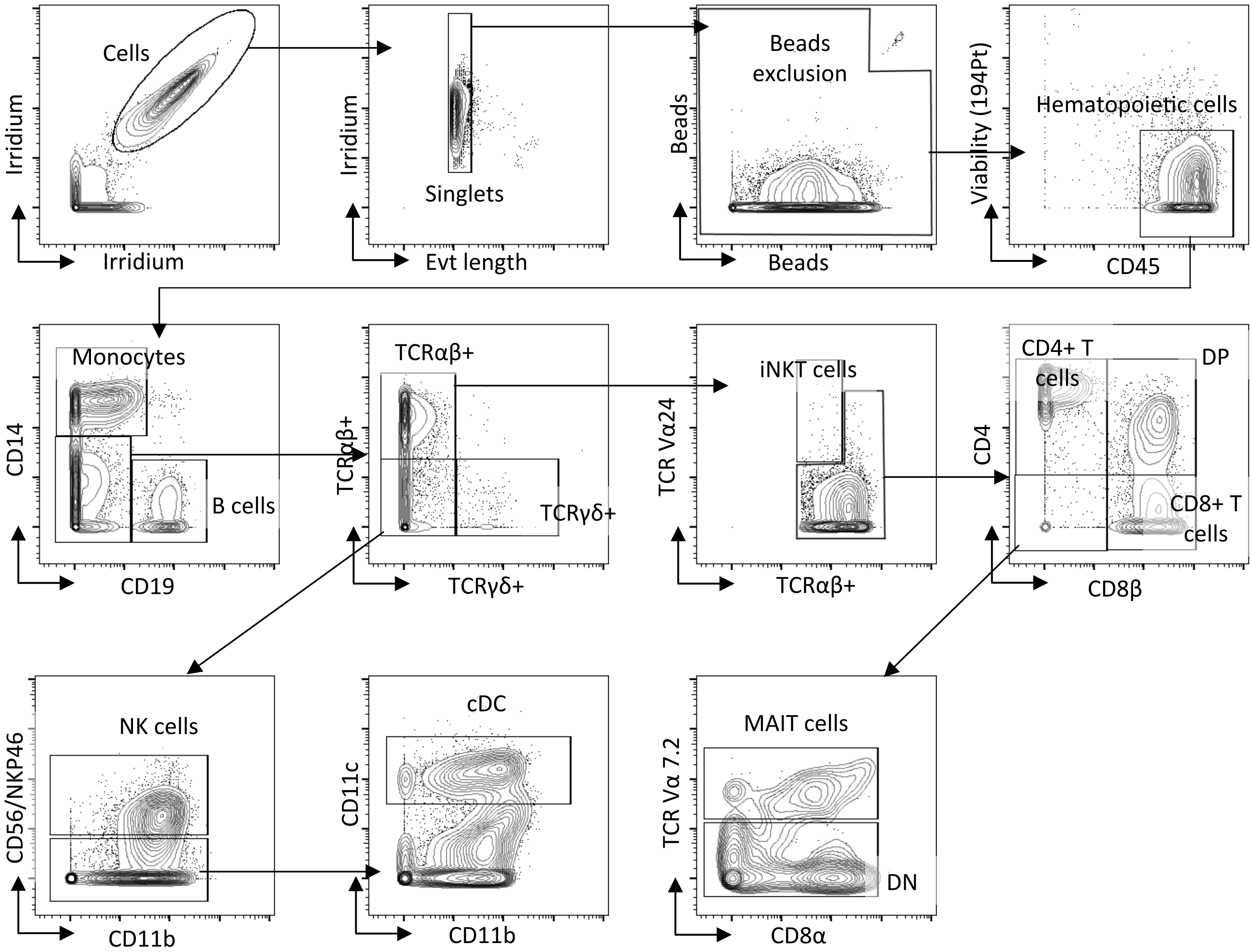
Cells
Irridium
Irridium
Singlets
Irridium
Evt length
Beads exclusion
Beads
Beads
Viability (194Pt)
Hematopoietic cells
CD45
Monocytes
CD14
B cells
CD19
TCRαβ+
TCRαβ+
TCRγδ+
TCRγδ+
iNKT cells
TCR Vα24
TCRαβ+
CD4+ T cells
DP
CD4
CD8+ T cells
CD8β
NK cells
CD56/NKP46
CD11b
cDC
CD11c
CD11b
MAIT cells
TCR Vα 7.2
DN
CD8α

Figure S1

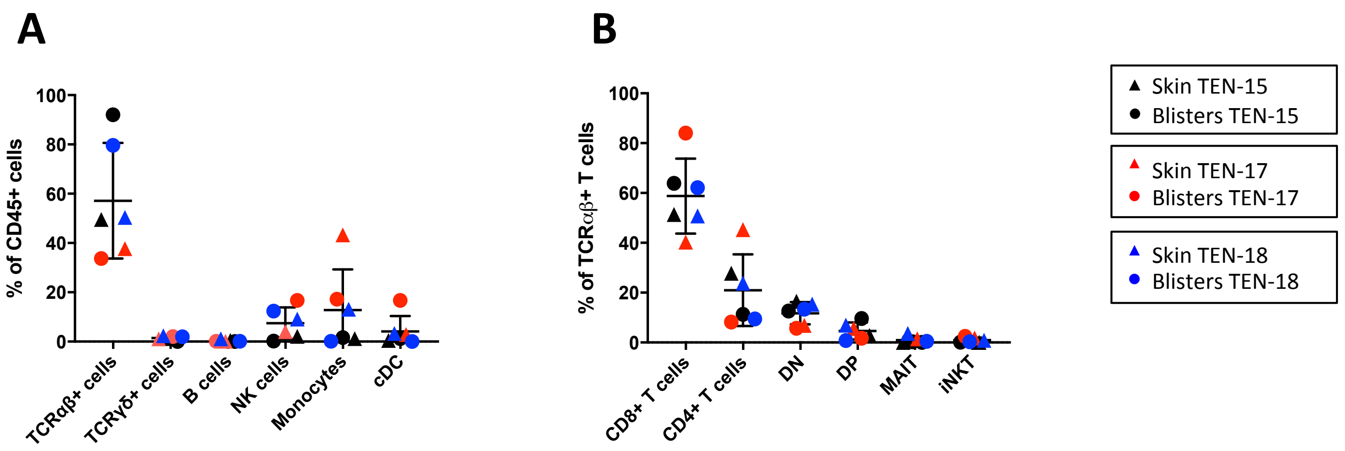
A
B
100
80
60
40
20
0
% of CD45+ cells
TCRαβ+ cells
TCRγδ+ cells
B cells
NK cells
Monocytes
cDC
% of TCRαβ+ T cells
CD8+ T cells
CD4+ T cells
DN
DP
MAIT
iNKT
Skin TEN-15
Blisters TEN-15
Skin TEN-17
Blisters TEN-17
Skin TEN-18
Blisters TEN-18

Figure S2

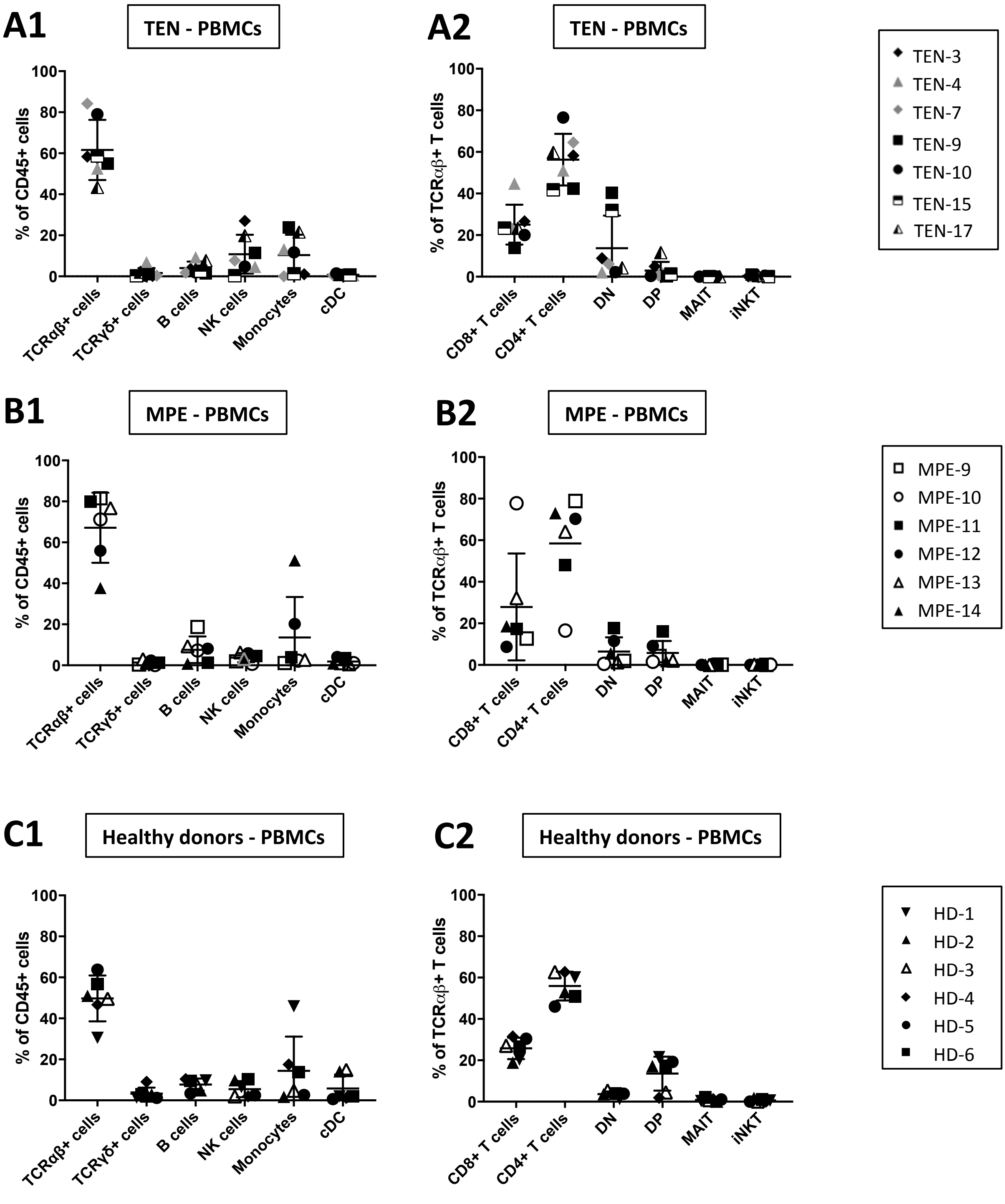
A1
TEN - PBMCs
% of CD45+ cells
TCRαβ+ cells
TCRγδ+ cells
B cells
NK cells
Monocytes
cDC
A2
TEN - PBMCs
% of TCRαβ+ T cells
CD8+ T cells
CD4+ T cells
DN
DP
MAIT
iNKT
TEN-3
TEN-4
TEN-7
TEN-9
TEN-10
TEN-15
TEN-17
B1
MPE - PBMCs
B2
MPE - PBMCs
MPE-9
MPE-10
MPE-11
MPE-12
MPE-13
MPE-14
C1
Healthy donors - PBMCs
C2
Healthy donors - PBMCs
HD-1
HD-2
HD-3
HD-4
HD-5
HD-6

Figure S3

Concatenated CD8 + T cells
TEN-MPE-Healthy donors
Skin/Blisters and PBMCs

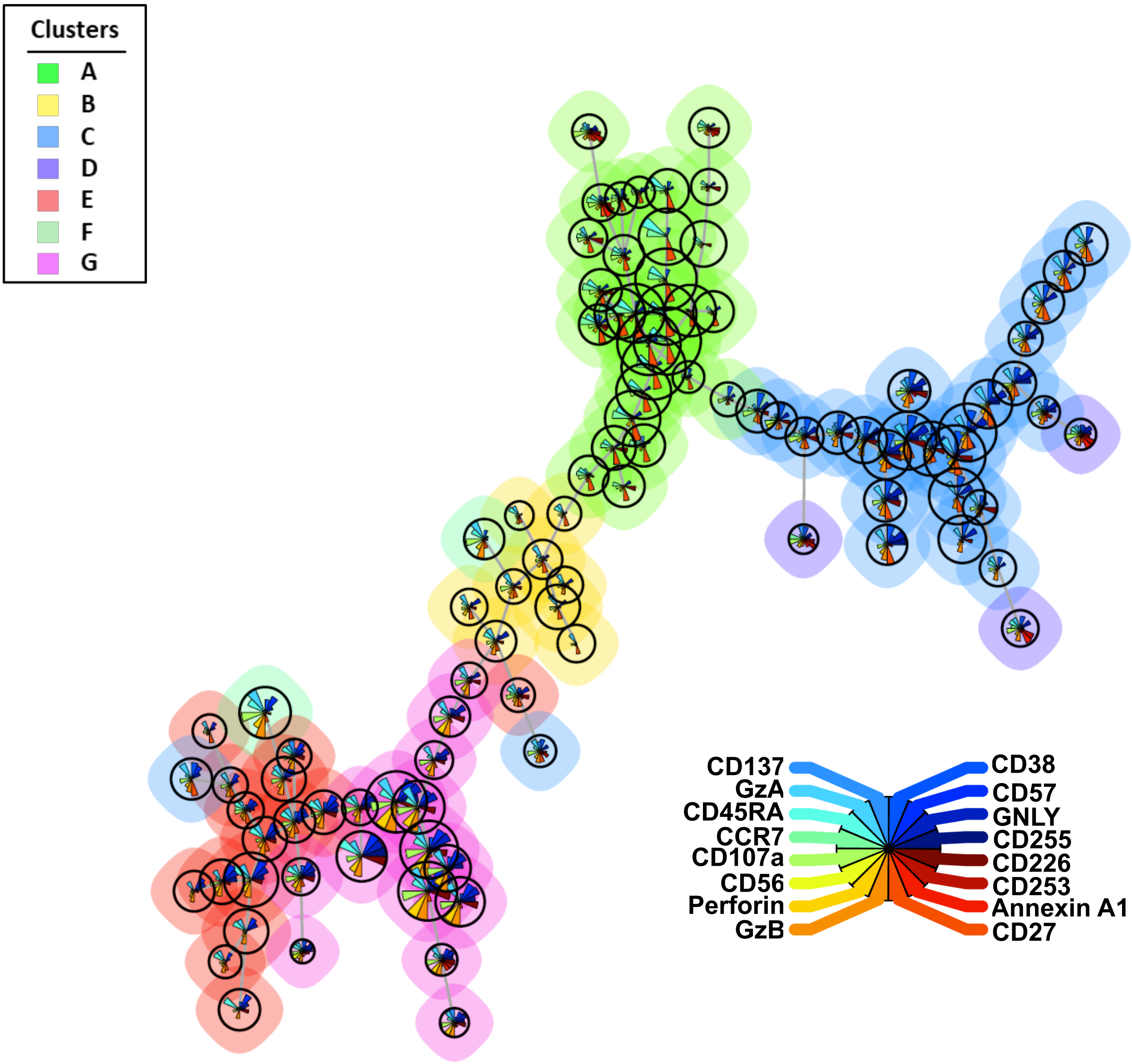

Figure S4

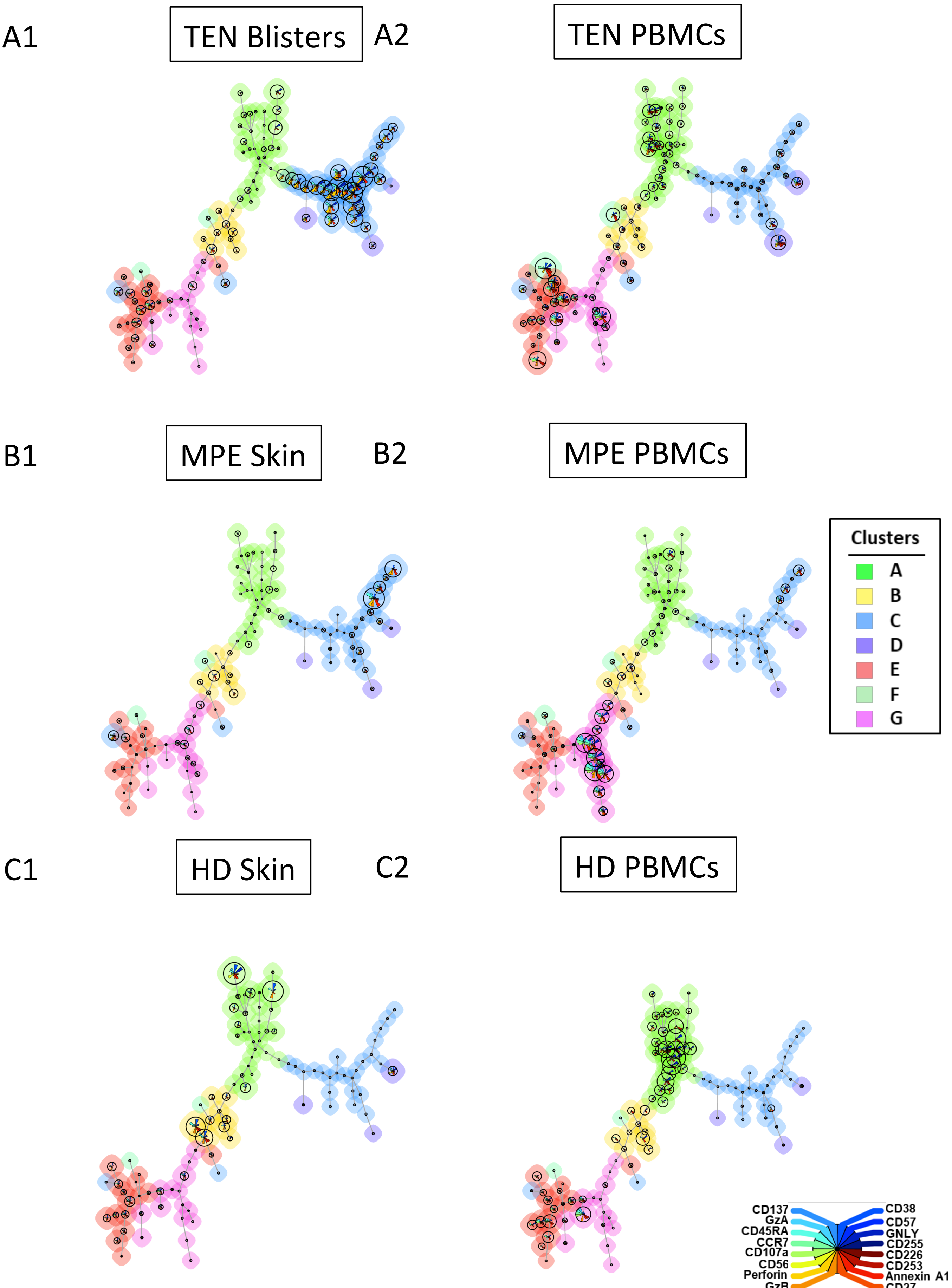
A1
TEN Blisters
A2
TEN PBMCs
B1
MPE Skin
B2
MPE PBMCs
Clusters
A
B
C
D
E
F
G
C1
HD Skin
C2
HD PBMCs
CD137
GzA
CD45RA
CCR7
CD107a
CD56
Perforin
GzB
CD38
CD57
GNLY
CD255
CD226
CD253
Annexin A1
CD27

Figure S5

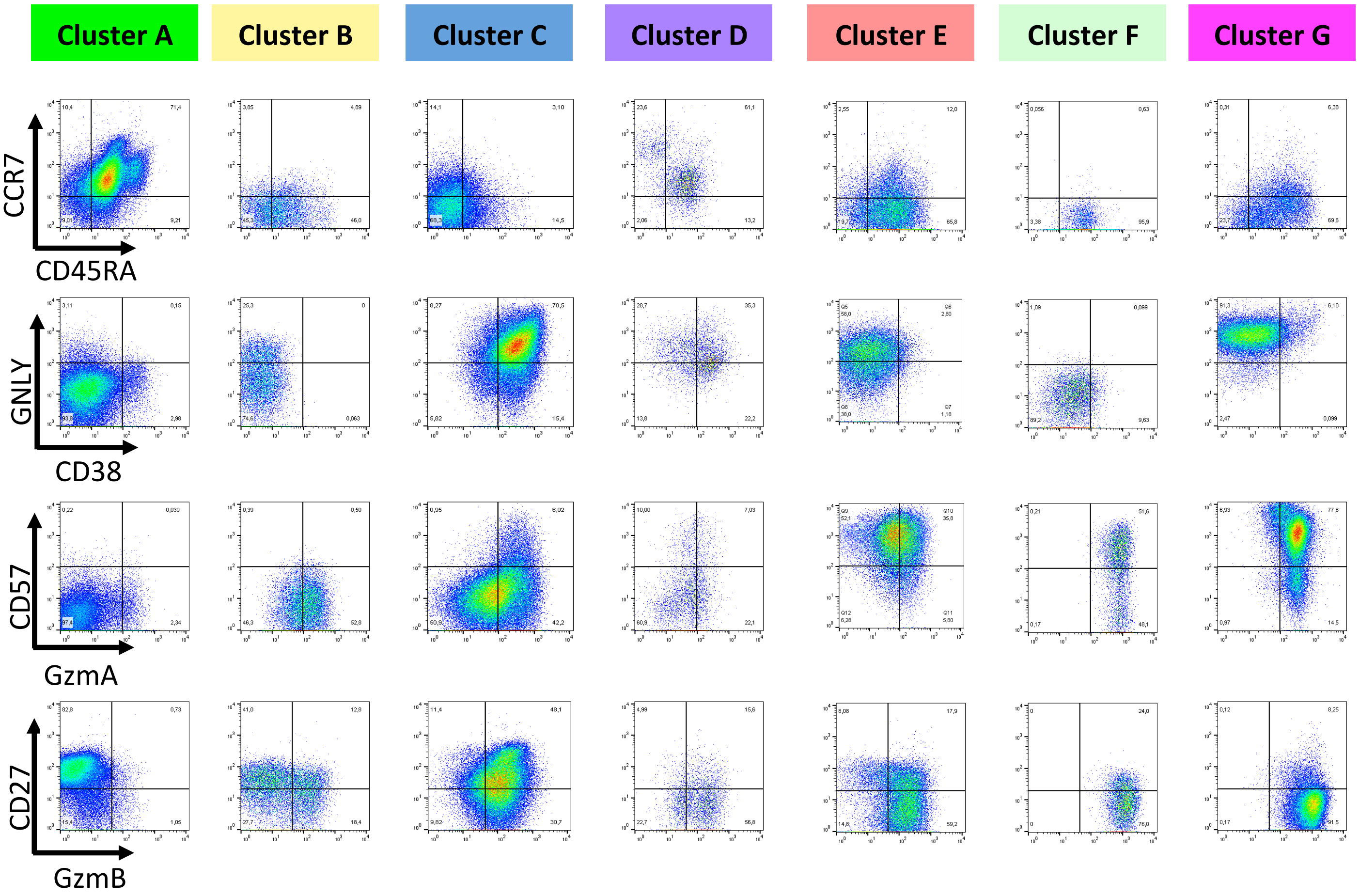
Cluster A
Cluster B
Cluster C
Cluster D
Cluster E
Cluster F
Cluster G
CCR7
CD45RA
GNLY
CD38
CD57
GzmA
CD27
GzmB

Figure S6

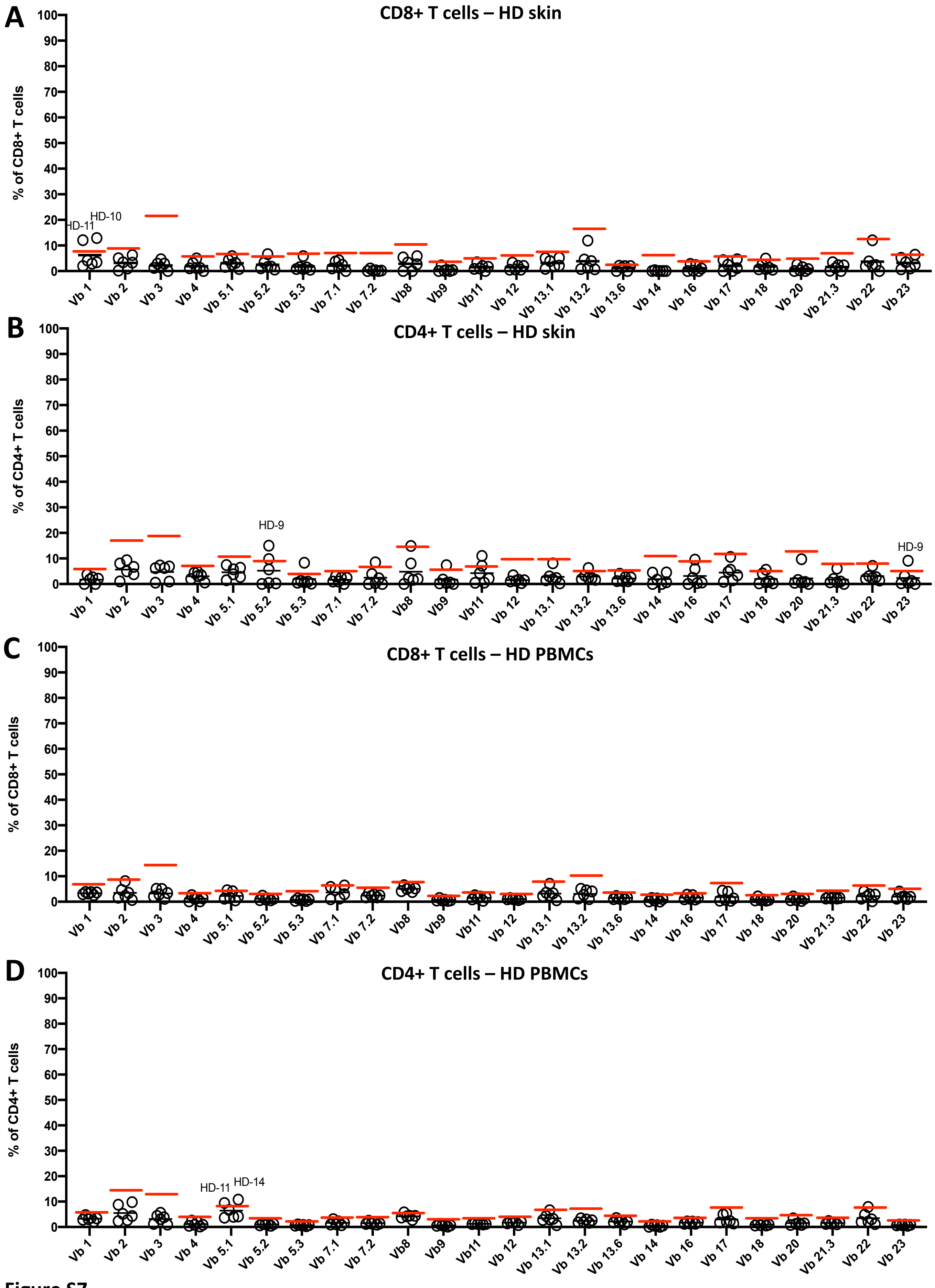
A
CD8+ T cells – HD skin
% of CD8+ T cells
HD-11
HD-10
B
CD4+ T cells – HD skin
% of CD4+ T cells
HD-9
HD-9
C
CD8+ T cells – HD PBMCs
% of CD8+ T cells
D
CD4+ T cells – HD PBMCs
% of CD4+ T cells
HD-11
HD-14
0
10
20
30
40
50
60
70
80
90
100
Vb 1
Vb 2
Vb 3
Vb 4
Vb 5.1
Vb 5.2
Vb 5.3
Vb 7.1
Vb 7.2
Vb8
Vb9
Vb11
Vb 12
Vb 13.1
Vb 13.2
Vb 13.6
Vb 14
Vb 16
Vb 17
Vb 18
Vb 20
Vb 21.3
Vb 22
Vb 23

Figure S7

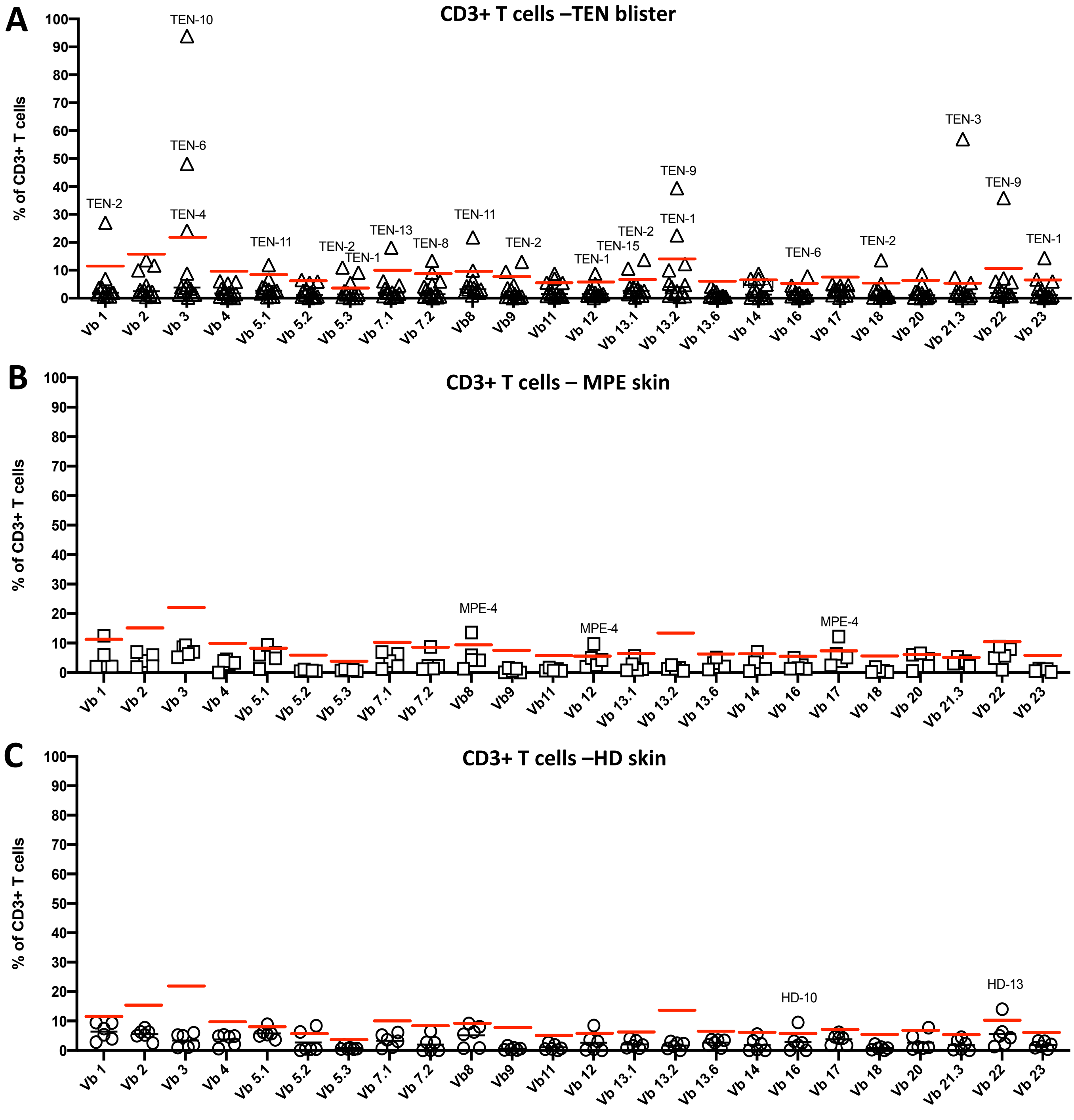
A
CD3+ T cells –TEN blister
% of CD3+ T cells
TEN-10
TEN-6
TEN-4
TEN-2
TEN-11
TEN-2
TEN-1
TEN-13
TEN-8
TEN-11
TEN-2
TEN-1
TEN-15
TEN-2
TEN-9
TEN-1
TEN-6
TEN-2
TEN-3
TEN-9
TEN-1
Vb 1
Vb 2
Vb 3
Vb 4
Vb 5.1
Vb 5.2
Vb 5.3
Vb 7.1
Vb 7.2
Vb8
Vb9
Vb11
Vb 12
Vb 13.1
Vb 13.2
Vb 13.6
Vb 14
Vb 16
Vb 17
Vb 18
Vb 20
Vb 21.3
Vb 22
Vb 23
B
CD3+ T cells – MPE skin
% of CD3+ T cells
MPE-4
MPE-4
MPE-4
C
CD3+ T cells –HD skin
% of CD3+ T cells
HD-10
HD-13

Figure S8

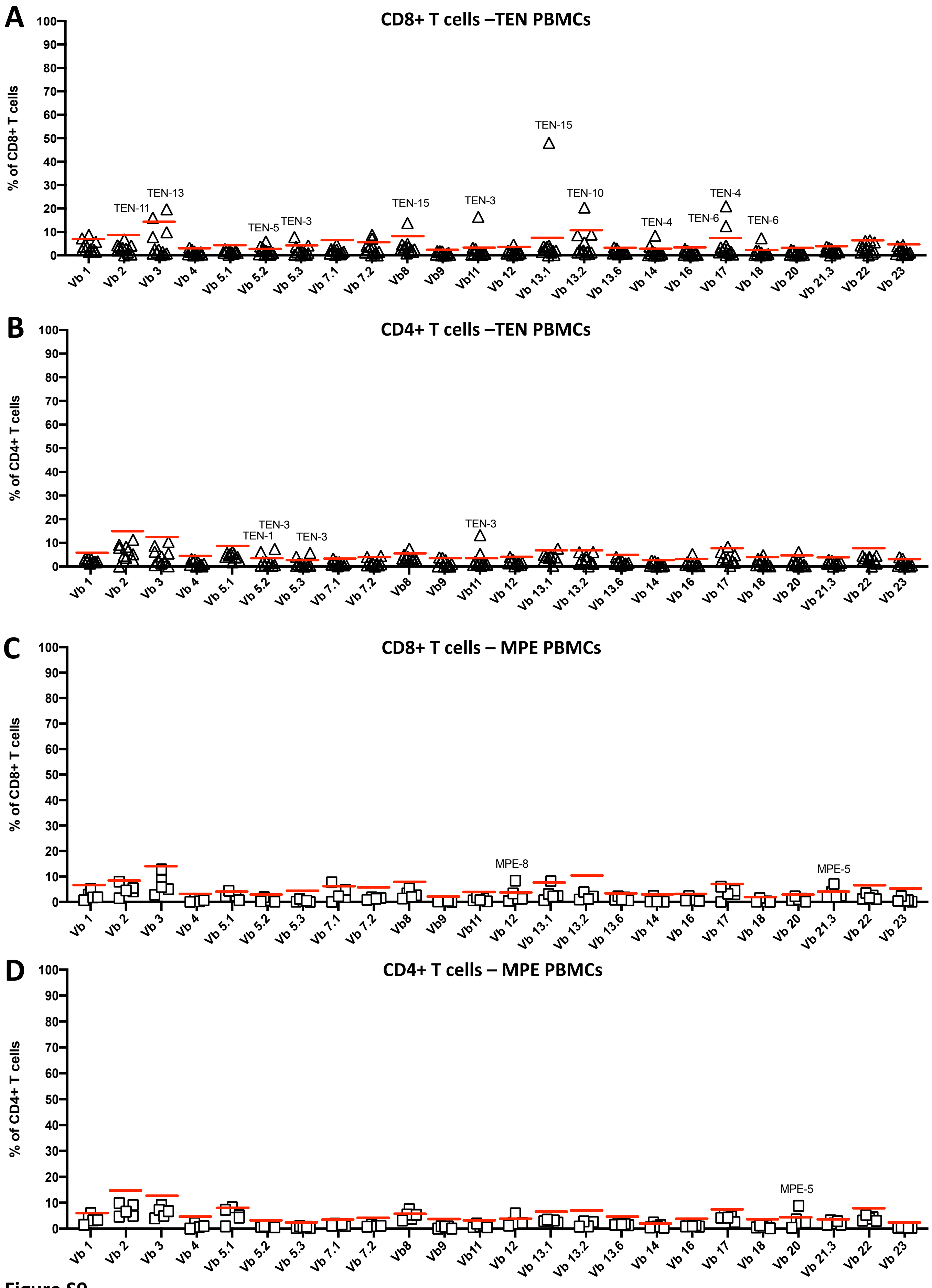
A
CD8+ T cells –TEN PBMCs
% of CD8+ T cells
TEN-15
TEN-13
TEN-11
TEN-5
TEN-3
TEN-15
TEN-3
TEN-10
TEN-4
TEN-4
TEN-6
TEN-6
Vb 1
Vb 2
Vb 3
Vb 4
Vb 5.1
Vb 5.2
Vb 5.3
Vb 7.1
Vb 7.2
Vb8
Vb9
Vb11
Vb 12
Vb 13.1
Vb 13.2
Vb 13.6
Vb 14
Vb 16
Vb 17
Vb 18
Vb 20
Vb 21.3
Vb 22
Vb 23
B
CD4+ T cells –TEN PBMCs
% of CD4+ T cells
TEN-3
TEN-1
TEN-3
TEN-3
C
CD8+ T cells – MPE PBMCs
% of CD8+ T cells
MPE-8
MPE-5
D
CD4+ T cells – MPE PBMCs
% of CD4+ T cells
MPE-5

Figure S9

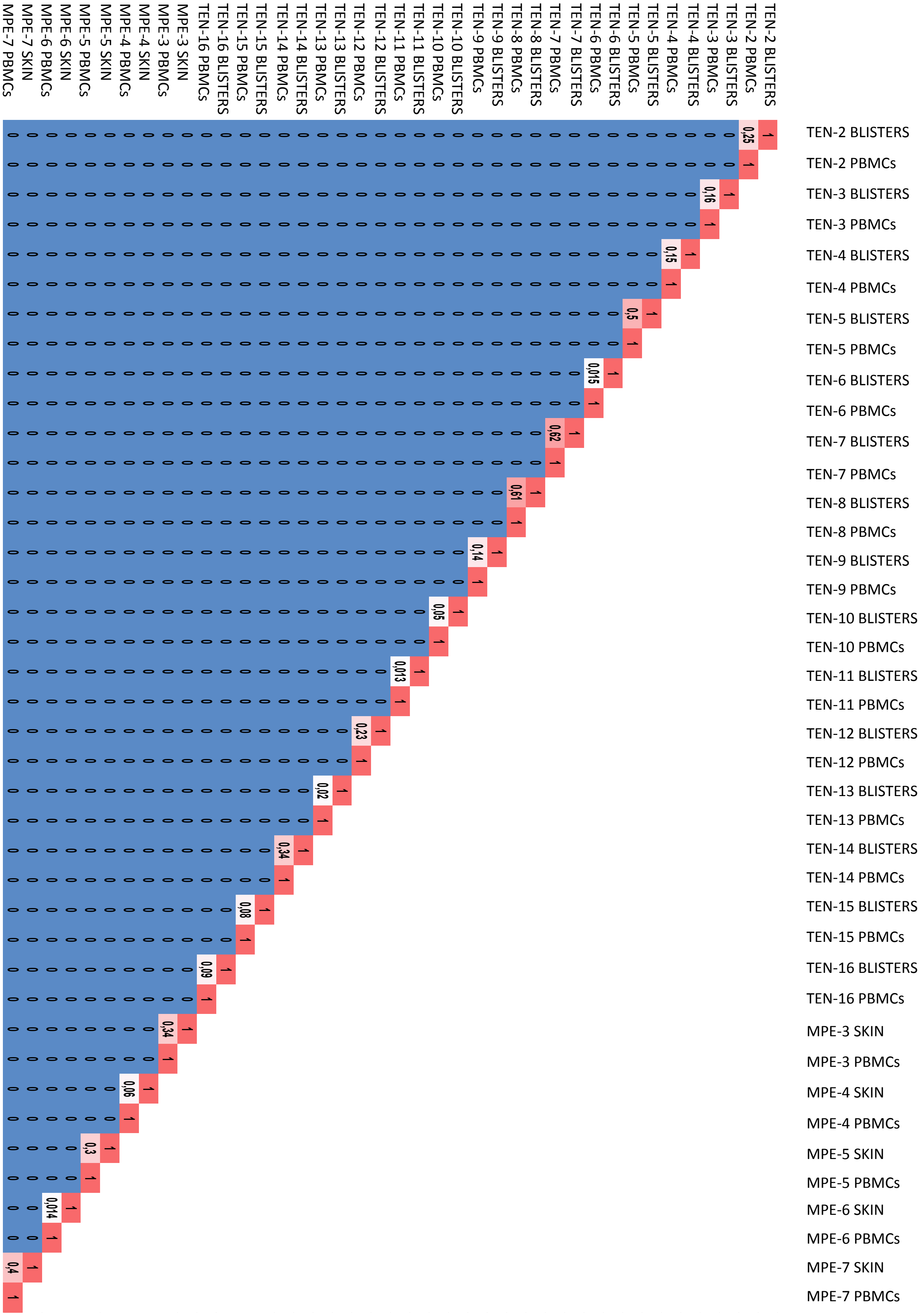

TEN-2 BLISTERS
TEN-2 PBMCs
TEN-3 BLISTERS
TEN-3 PBMCs
TEN-4 BLISTERS
TEN-4 PBMCs
TEN-5 BLISTERS
TEN-5 PBMCs
TEN-6 BLISTERS
TEN-6 PBMCs
TEN-7 BLISTERS
TEN-7 PBMCs
TEN-8 BLISTERS
TEN-8 PBMCs
TEN-9 BLISTERS
TEN-9 PBMCs
TEN-10 BLISTERS
TEN-10 PBMCs
TEN-11 BLISTERS
TEN-11 PBMCs
TEN-12 BLISTERS
TEN-12 PBMCs
TEN-13 BLISTERS
TEN-13 PBMCs
TEN-14 BLISTERS
TEN-14 PBMCs
TEN-15 BLISTERS
TEN-15 PBMCs
TEN-16 BLISTERS
TEN-16 PBMCs
MPE-3 SKIN
MPE-3 PBMCs
MPE-4 SKIN
MPE-4 PBMCs
MPE-5 SKIN
MPE-5 PBMCs
MPE-6 SKIN
MPE-6 PBMCs
MPE-7 SKIN
MPE-7 PBMCs
0,25
0,16
0,15
0,5
0,015
0,62
0,61
0,14
0,05
0,013
0,23
0,02
0,34
0,08
0,09
0,34
0,06
0,3
0,014
0,4

Figure S10

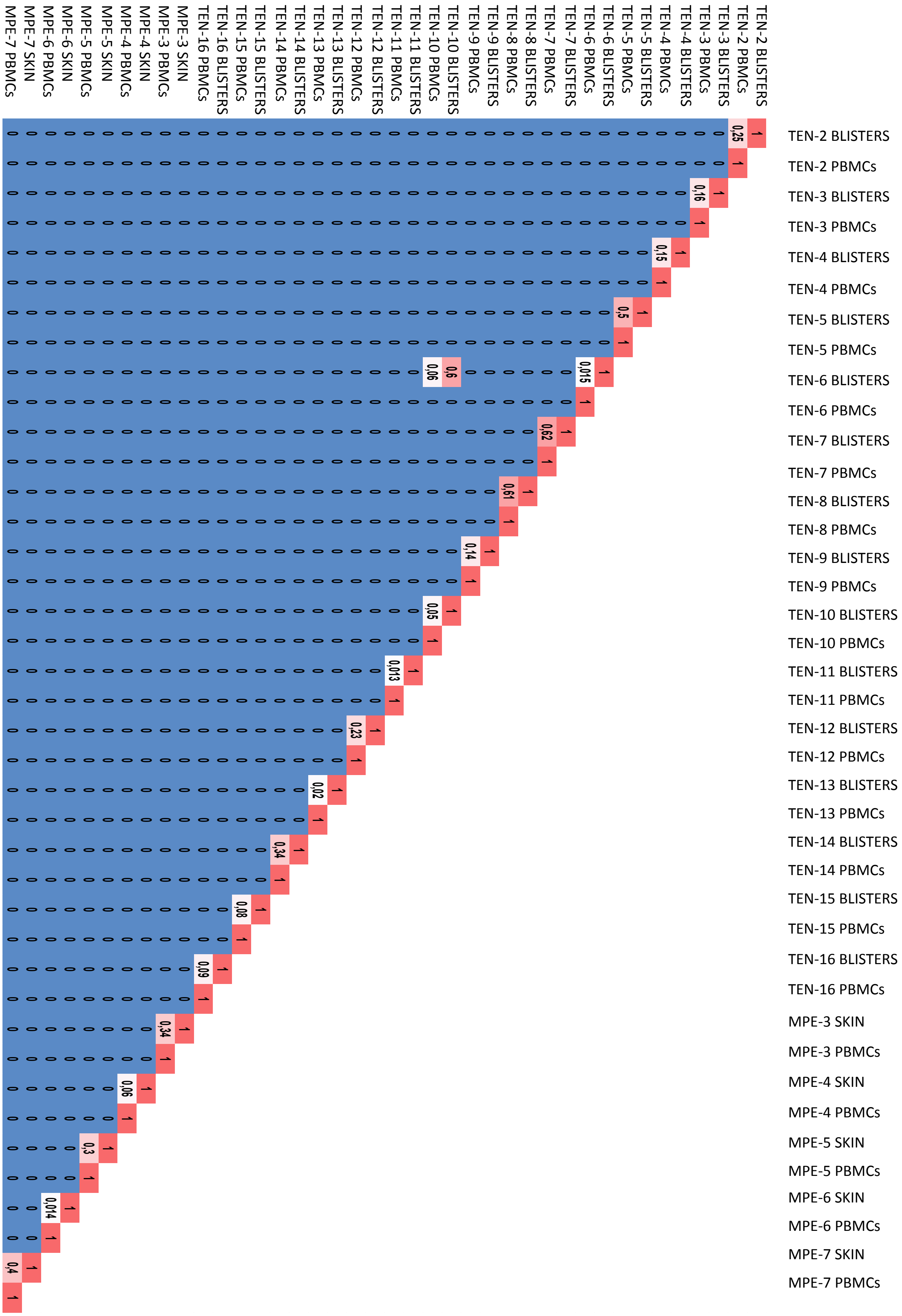
TEN-2 BLISTERS
TEN-2 PBMCs
TEN-3 BLISTERS
TEN-3 PBMCs
TEN-4 BLISTERS
TEN-4 PBMCs
TEN-5 BLISTERS
TEN-5 PBMCs
TEN-6 BLISTERS
TEN-6 PBMCs
TEN-7 BLISTERS
TEN-7 PBMCs
TEN-8 BLISTERS
TEN-8 PBMCs
TEN-9 BLISTERS
TEN-9 PBMCs
TEN-10 BLISTERS
TEN-10 PBMCs
TEN-11 BLISTERS
TEN-11 PBMCs
TEN-12 BLISTERS
TEN-12 PBMCs
TEN-13 BLISTERS
TEN-13 PBMCs
TEN-14 BLISTERS
TEN-14 PBMCs
TEN-15 BLISTERS
TEN-15 PBMCs
TEN-16 BLISTERS
TEN-16 PBMCs
MPE-3 SKIN
MPE-3 PBMCs
MPE-4 SKIN
MPE-4 PBMCs
MPE-5 SKIN
MPE-5 PBMCs
MPE-6 SKIN
MPE-6 PBMCs
MPE-7 SKIN
MPE-7 PBMCs
0,25
0,16
0,15
0,5
0,06
0,6
0,015
0,62
0,61
0,14
0,05
0,013
0,23
0,02
0,34
0,08
0,09
0,34
0,06
0,3
0,014
0,4
1
0

Figure S11

TEN	MPE

TEN-2

TRBV	Vβ	Frequency
TRBV06	Vβ 13,6	0.65
TRBV09	Vβ 1	0.43
TRBV06	Vβ 13,1	0.35
TRBV05	Vβ 5,1	0.29
TRBV06	Vβ 13,1	0.28

TEN-3

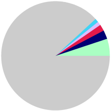

TRBV	Vβ	Frequency
TRBV11	Vβ 21,3	5.07
TRBV27	Vβ 14	2.57
TRBV09	Vβ 1	1.95
TRBV28	Vβ 3	1.13
TRBV07	na	1.08

TEN-4

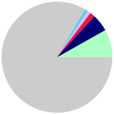

TRBV	Vβ	Frequency
TRBV19	Vβ 17	8.11
TRBV27	Vβ 14	4.99
TRBV07	na	1.56
TRBV07	na	1.04
TRBV06	Vβ 13,1	0.73

TEN-5

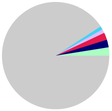

TRBV	Vβ	Frequency
TRBV13	Vβ 23	2.51
TRBV15	na	2.28
TRBV28	Vβ 3	2.19
TRBV06	Vβ 13,1	1.18
TRBV27	Vβ 14	1.00

TEN-6

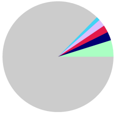

TRBV	Vβ	Frequency
TRBV07	na	4.83
TRBV18	Vβ 18	2.50
TRBV19	Vβ 17	2.13
TRBV30	Vβ 20	2.08
TRBV27	Vβ 14	1.10

TEN-7

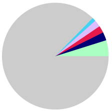

TRBV	Vβ	Frequency
TRBV06	Vβ 13,1/2/6	11.20
TRBV02	Vβ 22	8.25
TRBV20	na	3.04
TRBV06	na	1.99
TRBV05	Vβ 5,3	1.29

TEN-8

TRBV	Vβ	Frequency
TRBV11	na	0.55
TRBV27	Vβ 14	0.31
TRBV04	Vβ 7,2	0.18
TRBV27	Vβ 14	0.17
TRBV04	Vβ 7,2	0.13

TEN-9

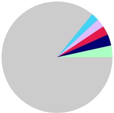

TRBV	Vβ	Frequency
TRBV29	Vβ 4	3.40
TRBV21	na	3.25
TRBV06	na	2.66
TRBV24	na	2.51
TRBV02	Vβ 22	2.22

TEN-10

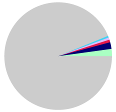

TRBV	Vβ	Frequency
TRBV28	Vβ 3	2.16
TRBV06	na	2.01
TRBV27	Vβ 14	0.80
TRBV04	Vβ 7,2	0.67
TRBV24	na	0.50

TEN-11

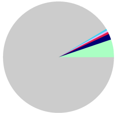

TRBV	Vβ	Frequency
TRBV28	Vβ 3	5.18
TRBV07	na	1.67
TRBV27	Vβ 14	0.65
TRBV10	Vβ 12	0.51
TRBV19	Vβ 17	0.48

TEN-12

TRBV	Vβ	Frequency
TRBV27	Vβ 14	0.86
TRBV03	na	0.21
TRBV14	Vβ 16	0.19
TRBV05	na	0.18
TRBV07	na	0.17

TEN-13

TRBV	Vβ	Frequency
TRBV28	Vβ 3	0.81
TRBV11	na	0.44
TRBV06	na	0.25
TRBV05	Vβ 5,1	0.21
TRBV06	Vβ 13,1	0.18

TEN-14

TRBV	Vβ	Frequency
TRBV06	Vβ 13,1	0.30
TRBV07	na	0.27
TRBV07	na	0.24
TRBV05	Vβ 5.2	0.15
TRBV29	Vβ 4	0.14

TEN-15

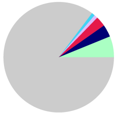

TRBV	Vβ	Frequency
TRBV06	Vβ 13.1	6.17
TRBV21	na	3.60
TRBV12	Vβ 8	2.96
TRBV07	na	1.02
TRBV12	Vβ 8	0.96

TEN-16

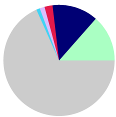

TRBV	Vβ	Frequency
TRBV19	Vβ 17	13.50
TRBV12	Vβ 8	13.38
TRBV10	Vβ 12	2.35
TRBV02	Vβ 22	1.43
TRBV07	na	1.02

MPE-3

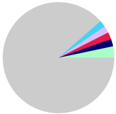

TRBV	Vβ	Frequency
TRBV05	na	3.3
TRBV05	Vβ 5,2	2.1
TRBV04	Vβ 7,2	2.1
TRBV15	na	2.0
TRBV11	Vβ 21,3	1.9

MPE-4

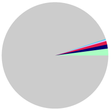

TRBV	Vβ	Frequency
TRBV07	na	2.1
TRBV06	na	1.2
TRBV07	na	1.1
TRBV05	na	0.4
TRBV07	na	0.3

MPE-5

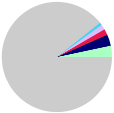

TRBV	Vβ	Frequency
TRBV04	Vβ 7,1	3.3
TRBV10	Vβ 12	3.3
TRBV20	na	1.8
TRBV07	na	1.5
TRBV07	na	0.8

MPE-6

TRBV	Vβ	Frequency
TRBV03	na	0.7
TRBV28	Vβ 3	0.6
TRBV07	na	0.1
TRBV28	Vβ 3	0.1
TRBV05	Vβ 5,1	0.1

MPE-7

TRBV	Vβ	Frequency
TRBV30	Vβ 20	1.0
TRBV03	na	0.6
TRBV09	Vβ 1	0.5
TRBV12	na	0.5
TRBV15	na	0.4

Figure S12

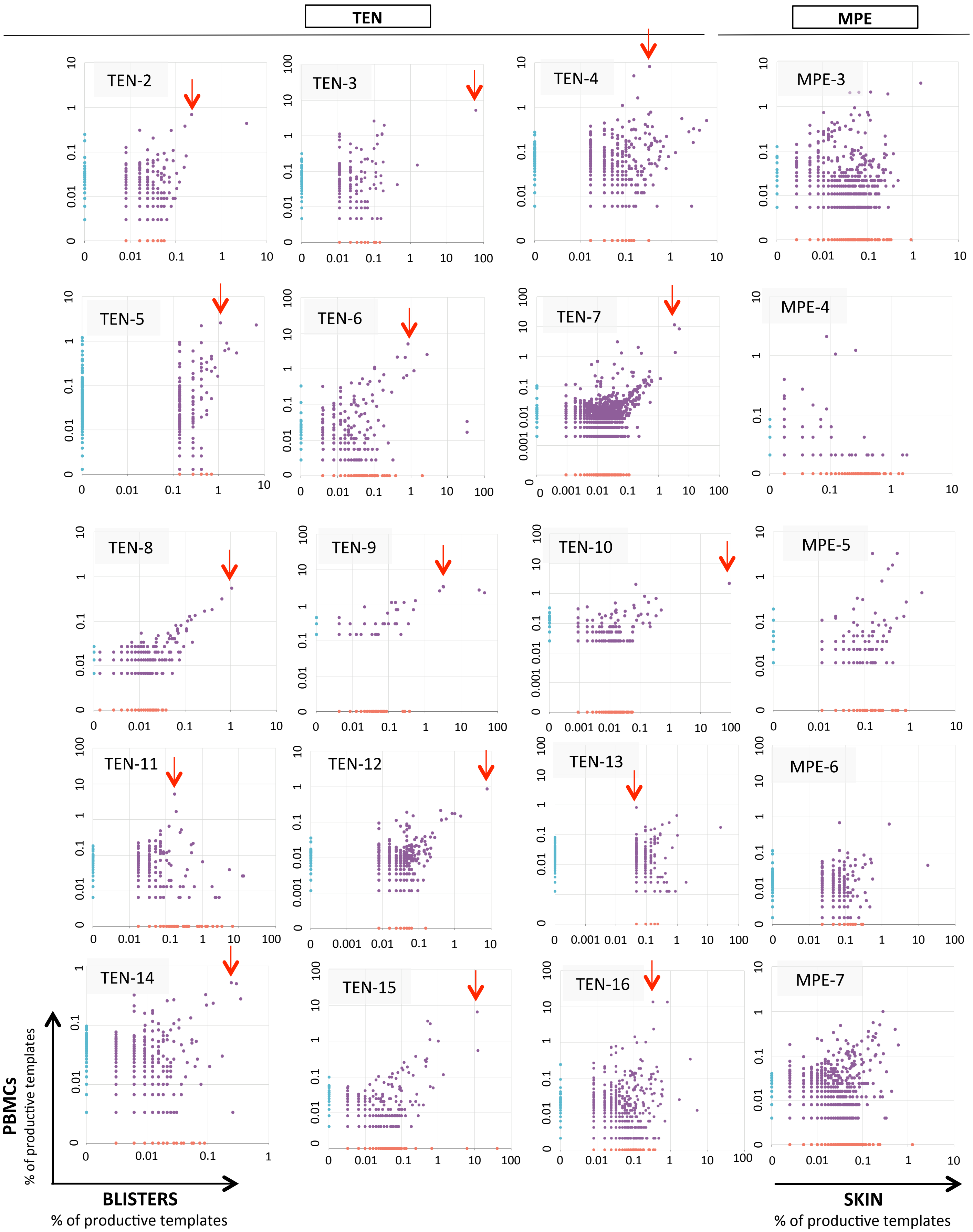

TEN
MPE
TEN-2
TEN-3
TEN-4
MPE-3
TEN-5
TEN-6
TEN-7
MPE-4
TEN-8
TEN-9
TEN-10
MPE-5
TEN-11
TEN-12
TEN-13
MPE-6
TEN-14
TEN-15
TEN-16
MPE-7
PBMCs
% of productive templates
BLISTERS
% of productive templates
SKIN
% of productive templates

Figure S13

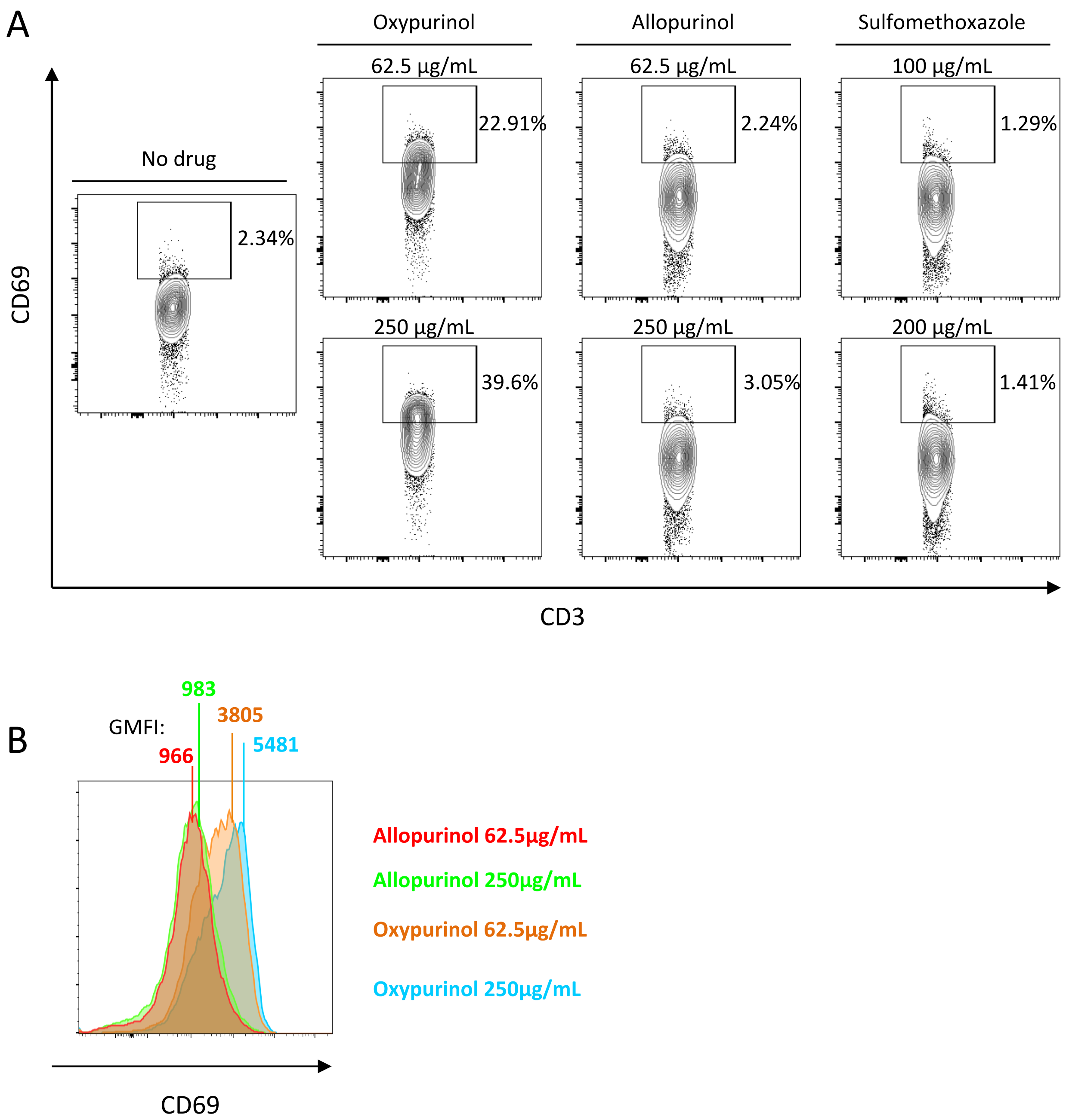
A
Oxypurinol
Allopurinol
Sulfomethoxazole
62.5 µg/mL
62.5 µg/mL
100 µg/mL
No drug
22.91%
2.24%
1.29%
2.34%
CD69
250 µg/mL
250 µg/mL
200 µg/mL
39.6%
3.05%
1.41%
CD3
B
GMFI:
983
3805
966
5481
Allopurinol 62.5µg/mL
Allopurinol 250µg/mL
Oxypurinol 62.5µg/mL
Oxypurinol 250µg/mL
CD69

Figure S14

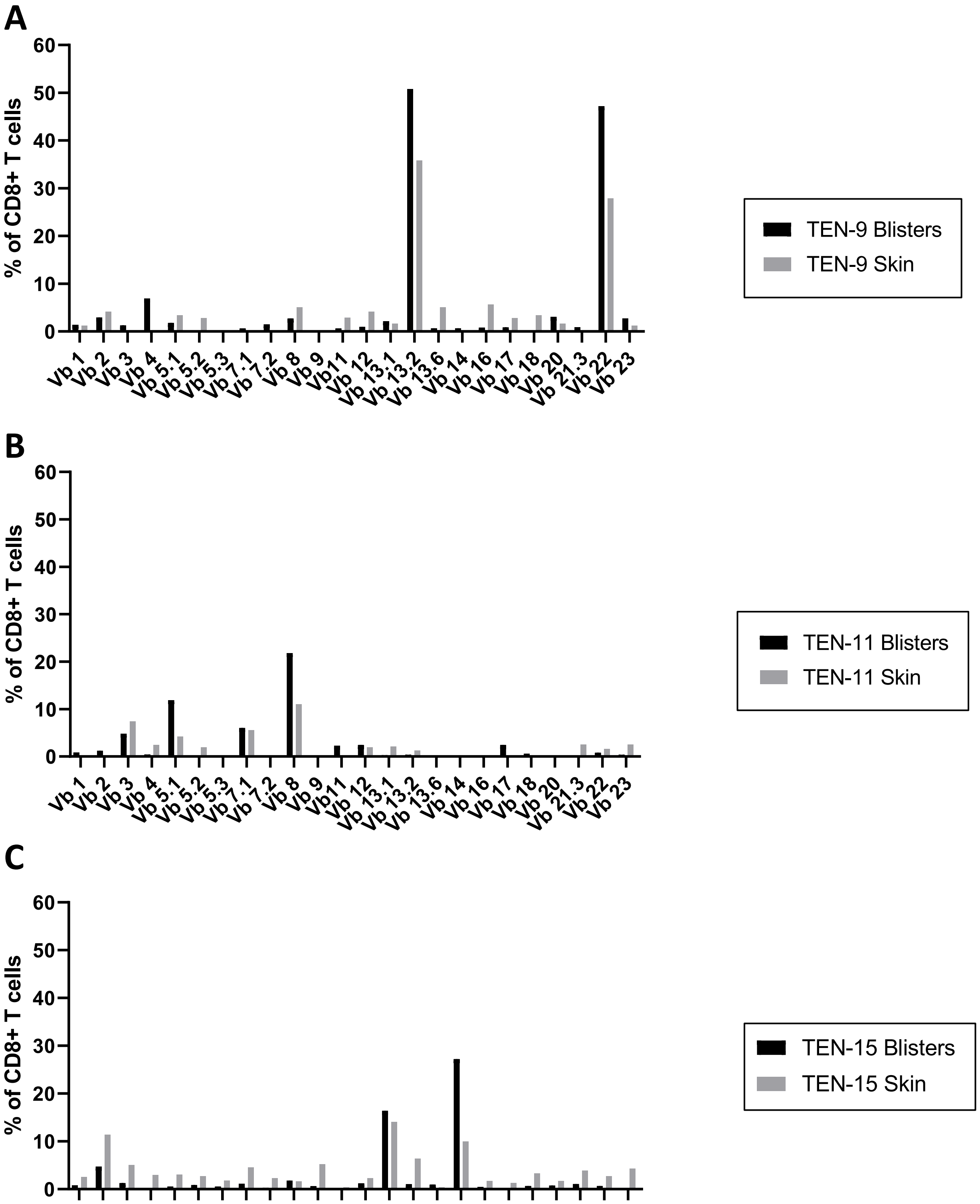
A
B
C
% of CD8+ T cells
TEN-9 Blisters
TEN-9 Skin
TEN-11 Blisters
TEN-11 Skin
TEN-15 Blisters
TEN-15 Skin

Figure S15

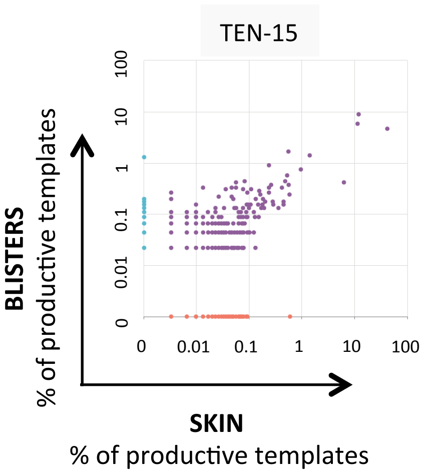

TEN-15
100
10
1
0.1
0.01
0
0
0.01
0.1
1
10
100
BLISTERS
% of productive templates
SKIN
% of productive templates

Figure S16

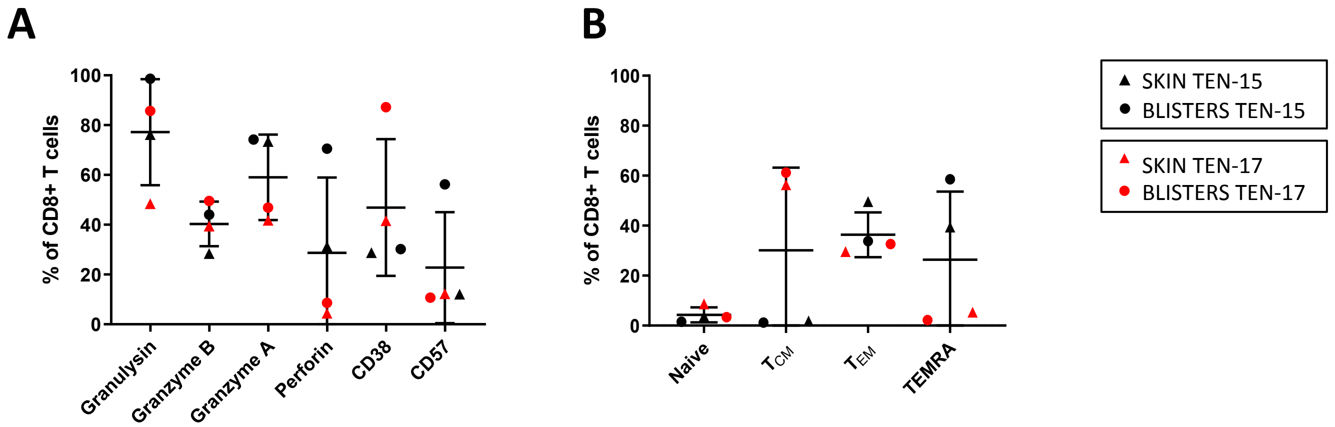
A
B
% of CD8+ T cells
100
80
60
40
20
0
Granulysin
Granzyme B
Granzyme A
Perforin
CD38
CD57
Naive
TCM
TEM
TEMRA
SKIN TEN-15
BLISTERS TEN-15
SKIN TEN-17
BLISTERS TEN-17

Figure S17

Supplementary Figure legends

Figure S1. Lineage gating strategy used for supervised analysis of mass cytometry data. Representative example of the gating strategy used to identify leucocyte lineages and T cell subpopulations in the blister, skin and PBMC samples from TEN, MPE and healthy donors. Cells were detected using iridium staining, and doublets and beads were excluded. Live (cisplatin negative (194 Pt)) CD45+ hematopoietic cells were then progressively subcategorized into different subpopulations: monocytes (CD14+), B cells (CD19+), conventional T cells (TCRαβ+), gamma delta T cells (TCRγδ+), NK cells (TCRαβ-TCRγδ-CD56+), conventional dendritic cells (cDC, CD11c+TCRαβ-TCRγδ-CD56-), invariant natural killer cells (iNKT, TCRαβ^{int} TCRVα24+) (*53*), CD4+ T cells (TCRαβ+CD4+), CD8+ T cells (TCRαβ+CD8β+), double positives (DP, TCRαβ+CD4+CD8β+), double negatives (DN, TCRαβ+CD4-CD8β-TCRVα7.2-) and MAIT cells (TCRαβ+CD4-CD8β-CD8α±TCRVα7.2+) (*54*).

Figure S2. Immunophenotyping of leucocytes present in TEN blisters and in adjacent skin samples. The leucocytes isolated from different bullae or adjacent non-bullous inflammatory skin from 3 subjects with TEN (black, red or blue symbols) were analyzed by mass cytometry. Scatter plots depict percentages of conventional TCRαβ+ lymphocytes, gamma delta T cells, B lymphocytes, NK cells, monocytes or conventional dendritic cells in hematopoietic CD45+ cells (**A**) and percentages of CD8+, CD4+, double negative and double positive T cell subsets, as well as iNKT and MAIT cells in gated TCRαβ+ population (**B**). Mean frequencies +/- SD are also indicated.

Figure S3: Immunophenotyping of PBMCs from TEN, MPE and healthy donors. The PBMCs from 7 TEN patients (**A**), 6 MPE patients (**B**) and 6 healthy donors (**C**) were analyzed by mass cytometry. Scatter plots depict percentages of conventional TCRαβ+ lymphocytes, gamma delta T cells, B lymphocytes, NK cells, monocytes or conventional dendritic cells in hematopoietic CD45+ cells (**A1-C1**), and percentages of CD8+, CD4+, double negative and double positive T cell subsets, as well as iNKT and MAIT cells in gated TCRαβ+ population (**A2-C2**). Mean frequencies +/- SD are also shown. The frequencies of each subset in TEN and MPE samples compared to healthy donor samples were not statistically different.

Figure S4: Minimal spanning tree magnification. All samples - Skin and PBMC of TEN, MEP and healthy donors. High-dimensional cell analysis using FlowSOM was conducted, as in Figure 2, on concatenated CD8+ T cell data (300 cells/sample) obtained from both blister, skin and PBMC samples from TEN and MPE patients and healthy donors (as reported in **Table S1)**. Minimal spanning tree

(with 100 nodes) and automatically subcategorized clusters (clusters A, B, C, D, E, F, G) are depicted. Each node includes phenotypically similar cells and the size of the node indicates the number of cell events. At the center of each node is represented a star chart. Each coloured star branch corresponds to the specific markers (CD45RA, CCR7, granzyme B (GzB), gA (GzA), granulysin (GNLY), perforin, CD27, CD38, CD56, CD57, CD107a, CD137, CD226, CD253, CD255, annexin A1) used for phenotypic comparison. The height of each star branch indicates the mean intensity: if the part reaches the border of the circle, the cells have a high expression for the marker.

Figure S5: Minimal spanning trees obtained after FlowSOM analysis of concatenated CD8+ T cell data from TEN, MPE and healthy donor samples. High-dimensional cell analysis using FlowSOM was conducted on concatenated CD8+ T cell data (300 cells/sample) from either blister, skin or PBMC samples from subjects with TEN (**A**), with MPE (**B**) and from healthy donors (**C**), as reported in **Table S1**.

Figure S6. FACS illustrations of the seven FlowSOM CD8+ T cell clusters. The 7 FlowSOM clusters (A-G) identified by high-dimensional analysis in Figure 2 were displayed for standard FACS plots visualization, using CCR7, CD45RA, granulysin (GNLY), CD38, CD57, granzyme A (GzA), CD27 and granzyme B (GzB) markers.

Figure S7: TCR Vβ repertoire usage in CD8+ and CD4+ T cell subsets isolated from the skin and PBMCs of healthy donors. The leucocytes isolated from the skin (**A & B**) and PBMCs (**C & D**) of 12 (HD-9 to HD-20) healthy donors were analysed by flow cytometry. Histograms depict percentages of the 24 TCR Vβ chains in gated CD8+ (**A & C**) and CD4+ (**B & D**) T cell subsets, using the IOTest® Beta Mark TCR Vβ

Repertoire Kit. Each round symbol represents a different subject. The red bar illustrates the threshold value from which TCR Vβ chains were considered as highly expanded (using Tukey's rule for the detection of outliers, i.e. Q3 + 1.5 x IQR).

Figure S8: TCR Vβ repertoire usage in CD3+ T cells isolated from the skin of TEN, MPE and healthy individuals. The leucocytes isolated from the blister fluids of 13 subjects with TEN (**A**), the lesional skin of 5 subjects with MPE (**B**) and the healthy skin of 6 donors **(C)** were analysed by flow cytometry, as in **Figure 3**. Each symbol (triangles for TEN, squares for MPE, rounds for healthy donors) represents a different subject. The red bar illustrates the threshold value from which TCR Vβ chains

were considered as highly expanded (using Tukey's rule for the detection of outliers, i.e. Q3 + 1.5 x IQR).

Figure S9: TCR Vβ repertoire usage in CD8+ and CD4+ T cell subsets isolated from PBMCs of TEN and MPE patients. PBMCs from 13 subjects with TEN (**A & B**) and 5 subjects with MPE **(C & D)** were analysed by flow cytometry. Histograms depict percentages of 24 TCR Vβ chains in gated CD8+ (**A & C**) and CD4+ (**B & D**) T cell subsets, using the IOTest® Beta Mark TCR Vβ Repertoire Kit. Each symbol (triangles for TEN, squares for MPE) represents a different subject. The red bar illustrates the threshold value from which TCR Vβ chains were considered as highly expanded (using Tukey's rule for the detection of outliers, i.e. Q3 + 1.5 x IQR).

TEN-2 (both CD8 and CD4+ T cells; not done) and TEN-15 (CD4+ T cells; technical issue) data are not depicted in the scatter plots.

Figure S10: Clonotype nucleotide sequence overlap between blister and PBMC samples from TEN and MPE patients. Morisita-Horn similarity index heatmap depicts overlap metrics (provided by ImmunoSEQ Analyzer V.3.0) for each possible pair-wise percent sharing between all pairs of TEN and MPE samples. This was computed by averaging across the two ratios of shared sequencing reads for each sample. Blue squares illustrate low (<1%) or no reads sharing between samples, white being intermediate and red being the highest level of sharing. Figures at the center of each square indicate percent sharing.

Figure S11: Clonotype amino acid overlap between blister and PBMC samples from TEN and MPE patients. Morisita-Horn similarity index heatmap depicts overlap metrics (provided by ImmunoSEQ Analyzer V.3.0) for each possible pair-wise percent sharing between all pairs of TEN and MPE samples. This was computed by averaging across the two ratios of shared sequencing reads for each sample. Blue squares illustrate low (<1%) or no reads sharing between samples, white being intermediate and red being the highest level of sharing. Figures at the center of each square indicate percent sharing.

Figure S12: Frequency and TRBV usage of the highly expanded TCRβ clonotypes in PBMC samples. The leucocytes isolated from the PBMCs of 15 subjects with TEN and 5 subjects with MPE were evaluated using HTS of the TCR. Pie charts illustrate frequencies of the 5 most expanded TCRβ clonotypes (measured as % of unique CDR3 sequence among all productive rearrangements within a sample). Colours indicate the respective TRBV usage of each TCRβ clonotypes. Grey indicates the

remaining clonotypes found in the same sample. TCRβ chain amino acid sequences are also provided. Cross-reference for the corresponding anti-Vβ mAb nomenclature is also provided.

Figure S13: Frequency of the most expanded TCRβ clonotypes in the blood and the blisters or skin of TEN and MPE patients. Comparison of TCRβ-chain CDR3 sequences in paired blister, skin and PBMC samples. Each dot of the heat map scatters represent one clone and the percentage of total productive templates of this given clone in skin and PBMC samples of TEN or MPE patients. Clones uniquely found in skin samples are located on the x-axis in red, clones uniquely present in PBMCs are located on the y-axis in blue; and clones found in both samples are in violet. Most expanded clones in the blood are shown with an arrow (such clones were used for correlation calculations in Figure 6).

Figure S14: **FACS plot illustration of drug-induced activation of TCRαβ Skw3 transductants.** Skw3 cell lines engineered for the expression of TCRs bearing Vα and Vβ chains from the top clone found in patient TEN-3 were stimulated *in vitro* with EBV-transformed B cells in presence of graded doses of different drugs (oxypurinol, allopurinol and sulfamethoxazole), or left unpulsed (no drug). Contour plots represent the percentage of CD69 expression in CD3+Skw3 transductants (**A**). Histograms represent the geometric mean fluorescence intensity (GMFI) of CD69 marker. Each color represents a different stimulatory condition. The GMFI values are listed above each histogram (**B**).

Figure S15: TCR Vβ repertoire usage in T cell subsets isolated from blister and adjacent skin samples of patients TEN-9, -11 and -15. The leucocytes isolated from the blister and adjacent skin samples of 3 subjects with TEN (TEN-9, TEN-11 and TEN-15) were analyzed by flow cytometry. Histograms depict percentages of 24 TCR Vβ chains in gated CD8+ T cell subsets, using the IOTest® Beta Mark TCR Vβ Repertoire Kit.

Figure S16: Frequency of the most expanded TCRβ clonotypes in paired blister and adjacent skin samples of patient TEN-15. Comparison of TCRβ-chain CDR3 sequences in paired blister and adkacent skin samples. Each dot of the heat map scatters represents one clone and the percentage of total productive templates of this given clone in respective sample. Clones uniquely found in adjacent skin sample are located on the x-axis in red, clones uniquely present in blister sample are located on the y-axis in blue; and clones found in both samples are in violet.

Figure S17: Immunophenotyping of leucocytes present in TEN blisters and adjacent skin samples.

The leucocytes isolated from the blister (dots) and adjacent skin (triangles) samples of 2 subjects with TEN (TEN-15 in black and TEN-17 in red) were analyzed by mass cytometry. Scatter plots depict percentages ± SD of Granulysin, Granzyme B, Granzyme A, Perforin, CD38 and CD57 markers in total CD8+ T cells (**A**). Frequencies ± SD of naïve (CD45RA+CCR7+), central memory T cells (T_{CM}; CD45RA-CCR7+), effector memory T cells (T_{EM}; CD45RA-CCR7-) and effector memory T cells expressing CD45RA (T_{EMRA}; CD45RA+CCR7-) in total CD8+ T cells are also shown (**B**).
